# Transcribed-ultra conserved region expression is associated with outcome in high-risk neuroblastoma

**DOI:** 10.1186/1471-2407-9-441

**Published:** 2009-12-15

**Authors:** Paola Scaruffi, Sara Stigliani, Stefano Moretti, Simona Coco, Carla De Vecchi, Francesca Valdora, Alberto Garaventa, Stefano Bonassi, Gian Paolo Tonini

**Affiliations:** 1 Translational Paediatric Oncology, National Cancer Research Institute (IST), Largo R. Benzi 10, Genoa, 16132, Italy; 2 Molecular Epidemiology, National Cancer Research Institute (IST), Largo R. Benzi 10, Genoa, 16132, Italy; 3 Department of Oncology and Genetics (DOBIG), University of Genoa, Largo R. Benzi 10, Genoa, 16132, Italy; 4 Department of Hematology-Oncology, Gaslini Institute, Largo G. Gaslini 5, Genoa, 16148, Italy; 5 Clinical and Molecular Epidemiology, IRCCS San Raffaele Pisana, Via della Pisana 235, Roma, 00163, Italy

## Abstract

**Background:**

Neuroblastoma is the most common, pediatric, extra-cranial, malignant solid tumor. Despite multimodal therapeutic protocols, outcome for children with a high-risk clinical phenotype remains poor, with long-term survival still less than 40%. Hereby, we evaluated the potential of non-coding RNA expression to predict outcome in high-risk, stage 4 neuroblastoma.

**Methods:**

We analyzed expression of 481 Ultra Conserved Regions (UCRs) by reverse transcription-quantitative real-time PCR and of 723 microRNAs by microarrays in 34 high-risk, stage 4 neuroblastoma patients.

**Results:**

First, the comparison of 8 short- versus 12 long-term survivors showed that 54 UCRs were significantly (*P *< 0.0491) over-expressed in the former group. For 48 Ultra Conserved Region (UCRs) the expression levels above the cut-off values defined by ROC curves were strongly associated with good-outcome (OS: 0.0001 <*P *< 0.0185, EFS: 0.0001 <*P *< 0.0491). Then we tested the Transcribed-UCR (T-UCR) threshold risk-prediction model on an independent cohort of 14 patients. The expression profile of 28 T-UCRs was significantly associated to prognosis and at least 15 up-regulated T-UCRs are needed to discriminate (*P *< 0.0001) short- from long-survivors at the highest sensitivity and specificity (94.12%). We also identified a signature of 13 microRNAs differently expressed between long- and short-surviving patients. The comparative analysis of the two classes of non-coding RNAs disclosed that 9 T-UCRs display their expression level that are inversely correlated with expression of 5 complementary microRNAs of the signature, indicating a negative regulation of T-UCRs by direct interaction with microRNAs. Moreover, 4 microRNAs down-regulated in tumors of long-survivors target 3 genes implicated in neuronal differentiation, that are known to be over-expressed in low-risk tumors.

**Conclusions:**

Our pilot study suggests that a deregulation of the microRNA/T-UCR network may play an important role in the pathogenesis of neuroblastoma. After further validation on a larger independent set of samples, such findings may be applied as the first T-UCR prognostic signature for high-risk neuroblastoma patients.

## Background

Neuroblastoma (NB) is a pediatric tumor of the sympathetic nervous system characterized by a remarkable heterogeneous clinical behavior [1]. Patients with localized NB have a favorable outcome, and in infants with disseminated stage 4 tumor the progression of disease is generally halted by good response to therapy. Conversely, only 20-30% of children older than 12 months of age - or 18 months, according to a more recent classification [2] - with a stage 4 tumor show progression free- and overall-survival longer than 60 months, despite multimodal therapeutic protocols [3]. In recent years several prognostic signatures derived from gene expression profiles, DNA abnormalities, and microRNAs [4-9] have been proposed as sensitive indicators of tumor progression in NB patients. Yet, the major challenges remain to validate each gene classifier on independent patient cohorts, and to identify additional tumor-specific prognostic markers for improved risk estimation at the time of diagnosis, especially in high-risk NB patients.

Recently, Calin et al. [10] have demonstrated that a newly discovered class of Ultra Conserved Regions (UCRs) [11] is significantly altered at both DNA and RNA levels in adult chronic lymphocytic leukemias, colorectal and hepatocellular carcinomas. Moreover, they found that tumor-associated Transcribed-UCRs (T-UCRs) in leukemias are regulated by microRNAs, leading to the hypothesis that both coding and noncoding RNAs may define signatures associated with diagnosis, prognosis, and response to treatment. So far, there are no reports about the expression of T-UCRs in NB and there is presently only one published study on microRNA deregulation and NB prognosis [9]. Here we profiled both T-UCRs and microRNAs in a cohort of 34 high-risk NBs, with the aim to investigate their putative role as sensitive markers of outcome prediction in children with stage 4 NB, a major challenge to clinicians and researchers. Our pilot study suggests for the first time that a signature based on T-UCR expression is associated with good outcome in non infant patients diagnosed with metastatic NB, and lets us assume putative complex regulatory interplays between T-UCRs and microRNAs in neuroblastoma. The potential to use this information must be tested in a larger, independent set of samples.

## Methods

### Tumor samples

Tumor specimens were collected at the onset of disease from 34 patients who were diagnosed with a primary NB between 1990 and 2006, and referred to the Gaslini Children Hospital, Genoa, Italy. The study was approved by Ethics committee of the Gaslini Children Hospital, and informed consent was obtained by all children's legal guardians. All patients were classified as stage 4 according to the International Neuroblastoma Staging System [12], and they were older than 1 year of age at time of diagnosis (average age: 39.2 months). Regarding the clinical course, patients were categorized into two subgroups: "short-survivors" (dead of disease within 36 months from diagnosis. Deaths due to toxicity were censored) and "long-survivors" (alive with an overall survival time > 36 months). Long- and short-surviving patients did not differ by year of diagnosis. Patient and tumor characteristics are summarized in additional file 1: Table S1.

The study was initially conducted on a cohort of 20 patients: 8 short-survivors (median follow-up time: 18 months, range 6-34 months; median event-free surviving time: 14 months, range 5-29 months) and 12 long-survivors (median follow-up time: 86 months, range 39-164 months; median event-free surviving time: 72 months, range 22-164 months). None but 2 long-surviving patients (#1558 and #2056) had relapse or progression of disease. Case #1558 had a local relapse after 22 months from diagnosis and is in complete remission with a follow-up time of 112 months. Patient #2056 had a progression of disease after 70 months from diagnosis and at the time of analysis is alive with stable disease (follow-up time: 76 months).

Then, the T-UCR threshold risk-prediction model obtained from this group of patients was tested in an independent cohort of 14 patients. Within this set, 9 patients were included in the short-survivor group (median follow-up time: 18 months, range 1-35 months; event-free surviving time: 13 months, range 1-22 months) and 5 in the long-survivor one (median follow-up time: 53 months, range 40-67 months). The latter group of patients had neither relapse nor progression of disease.

All tumor samples were classified as Schwannian stroma-poor neuroblastoma according to the International Neuroblastoma Pathology Committee [13], with at least 80% of neuroblasts. Amplification of the *MYCN *oncogene was present in tumors of 5 short- and 2 long-surviving patients.

### Reverse transcription quantitative real-time PCR (RT-qPCR)

Total RNA was extracted from frozen tumors by PerfectPure™ RNA Tissue Kit (5Prime, Hamburg, Germany), including RNase-free DNase I treatment. Total and small RNAs were quantified and quality control was assessed by RNA 6000 Nano^® ^and Small RNA^® ^assays, respectively, on the 2100 Bioanalyzer (Agilent Technologies, Santa Clara, CA). Only samples with RNA integrity number ≥ 6.0 were included in the study. RNA samples have been validated as DNA-free by a no-reverse transcription assay for Eukaryotic 18S rRNA gene (VIC-labeled TaqMan Gene Expression assay, Applied Biosystems, Foster City, CA). RNA was amplified and reverse transcribed by WT-Ovation™ RNA Amplification System kit (NuGEN Technologies, San Carlos, CA), and UCR transcripts were quantified by Transcribed Ultra Conserved Regions real-time PCR assays (PrimerDesign Ltd, Hants, UK) using SYBR^® ^green detection chemistry. Reactions were setup in 96-white-well Twin.tec^® ^real-time plates (Eppendorf, Hamburg, Germany) by EpMotion 5070 Liquid Handling Workstation (Eppendorf) and performed in duplicate on RealPlex^4 ^system (Eppendorf). Specificity of qPCR reactions has been assessed by melting curve analysis. According to the Minimum Information for Publication of Quantitative Real-Time PCR Experiments (MIQE) [14,15], raw qPCR data and a checklist including technical details are submitted as Supplemental data (see additional file 2: Raw Cq data; additional file 3: MIQE checklist). The relative amount of each T-UCR to 18S rRNA was determined using the equation 2^-*dCq*^, where dCq = (Cq_T-UCR _- Cq_18S rRNA_), as previously described [10].

### Statistical analyses

Student's *t*-test was used to compare mean T-UCR expression levels between long- and short-surviving individuals after logarithmic transformation of original measures. All significance tests were two tailed. To identify those values providing the best separation between long- and short-survivors, T-UCR expression values were dichotomized according to Receiver Operating Characteristic (ROC) curves [16]. The threshold values identified with this procedure were used in subsequent survival analyses. Overall survival (OS) was defined as the time elapsed from diagnosis to cancer-related death or to the date of the last follow-up if the patient survived. Patients who survived were censored at the last date they were reported to be alive. Event free survival (EFS) was calculated from diagnosis to the date of tumor progression or relapse or to the date of the last follow-up if no event occurred. Survival curves were computed according to the Kaplan-Meier method. The Cox proportional-hazards regression model was used to study in a multivariate setting the effect of *MYCN *status (amplified, not amplified) as potential confounder. A backward selection approach was used and only variables with *P *< 0.05 were retained in the model. The MedCalc^® ^software (Mariakerke, Belgium) was used for statistical analyses.

### Oligonucleotide array CGH

Array CGH was performed on frozen tumors as previously described [17] using 44K microarrays (Agilent Technologies). Array CGH data have been deposited in Gene Expression Omnibus (GEO, http://www.ncbi.nlm.nih.gov/geo/) and they are accessible through GEO Series accession number GSE14109.

### microRNA expression profiling

We performed microRNA expression profiling starting from 100 ng of total RNA and following the microRNA Microarray System protocol v.1.5 (Agilent Technologies). In dephosphorylation and ligation steps, 17 Units of Calf Intestine Alkaline Phosphatase (Takara Bio Inc., Shiga, Japan) and 20 Units of T4 RNA ligase (Takara Bio Inc.) have been used, respectively. Each sample was hybridized on Human microRNA Microarray v.2 (Agilent Technologies) containing probes for 723 human microRNAs. Slides were scanned by Agilent G2565BA scanner and images were processed by Feature Extraction software v.9.5.3.1 (Agilent Technologies). Tab-delimited text file were analyzed in R v.2.7.2 software environment http://www.r-project.org using the *limma *package v.2.14.16 of Bioconductor http://www.bioconductor.org. Only spots with signal minus background flagged as positive and significant were used in the following analysis as 'detected' spot. Probes with less than 50% of detected spots across all arrays, and arrays with a number of detected spots smaller than 50% of all spots on the array were removed. Background corrected intensities of replicated spots on each array were averaged. Data were then log2-transformed and normalized for between-array comparison using *quantile *normalization [18]. Differential expression analysis between "long-survivors" and "short-survivors" category was carried out using Student's *t*-test (unpaired, two-tailed, unequal variance). MicroRNAs with *P*-values < 0.05 were selected for further analysis. Given the explorative nature of this study, no correction for multiple testing was applied in the screening procedure aimed at selecting multiple sets of microRNAs for subsequent hierarchical clustering analyses. The agglomerative hierarchical clusters, used to detect similarity relationships in microRNA log2-transformed expressions, were computed by the Euclidean distance between single vectors and the Ward method [19]. Microarray data have been deposited in GEO (Series accession number GSE16444).

### Functionally prediction of T-UCR signature

T-UCR::microRNA sequence comparison was performed by using the Basic Local Alignment Search Tool available in the UCbase & miRfunc database http://microrna.osu.edu/UCbase4/[20]. Putative targets of microRNAs were determined by the miRBase Targets v.5 http://microrna.sanger.ac.uk/targets/v5/ prediction algorithm.

## Results and Discussion

### T-UCR expression profiling of NB tumors

We investigated the expression of 481 T-UCRs by RT-qPCR in two independent study groups of high-risk NB patients. Overall, 34 NBs were investigated, including 17 tumors of patients who died of disease and 17 samples of long-survivors. About 96% of T-UCRs were expressed in NB samples at variable levels, with the exception of 15 transcripts (uc.82, uc.105, uc.160, uc.204, uc.213, uc.284, uc.299, uc.314, uc.327, uc.344, uc.347, uc.357, uc.363, uc.398, uc.407) that were undetectable in all samples. Six T-UCRs (uc.88, uc.95, uc.157, uc.162, uc.361, uc.394) showed a Cq mean value above 35 and were therefore considered as not expressed. The majority of T-UCRs found in NB samples are nonexonic (53%), whereas exonic and possibly exonic T-UCRs are transcribed at a lower frequency (23.3% and 23.6%, respectively).

### T-UCR signature of NBs

T-UCR transcription levels were initially quantified in a set of 20 metastatic NBs of 8 short- and 12 long-survivors. Fifty-four of the 460 detectable T-UCRs (11.7%) showed a differential expression between the two groups (0.0004 <*P *< 0.0491) (see additional file 4: Table S2; additional file 5: Figure S1). The predictive value of the 54 differentially expressed on survival was analyzed in univariate model utilizing the thresholds defined by the ROC curves (see additional file 4: Table S2; additional file 6: Figure S2). The Kaplan-Meier curves showed that T-UCR expression levels above the cut-off value were significantly associated with a more favorable outcome in all 54 T-UCR for OS (0.0001 <*P *< 0.0369), and only in 48 T-UCRs for EFS (0.0001 <*P *< 0.0491) (see additional file 4: Table S2; additional file 7: Figure S3).

Then, the T-UCR threshold risk-prediction model was tested in an independent cohort of 14 advanced stage NBs of 9 short- and 5 long-survivors. Kaplan-Meier survival analysis confirmed the significant association between higher expression level and better outcome for 28 T-UCRs (OS: 0.0007 <*P *< 0.0399, EFS: 0.0001 <*P *< 0.0399) (see additional file 4: Table S2; additional file 8: Figure S4).

**Figure 1:**
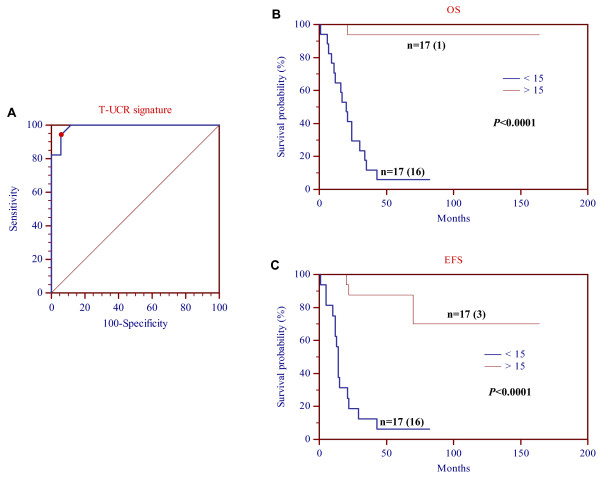
**(A) ROC curve**. Sensitivity and specificity in predicting survival based on different numbers of T-UCRs whose expression levels are above the thresholds defined by each respective ROC curve. The point marked with the red dot represents the highest average of sensitivity and specificity at the cut-off value of 15 (AUC = 0.988, Z statistic = 25.078, *P *= 0.0001). **Kaplan-Meier analysis for (B) overall and (C) event-free survival of all 34 NBs**. Comparison of survival probabilities of patients with ≥15 or <15 up-regulated T-UCRs. In each graph are reported the number of patients in predicted subgroups and the number of patients with event (between brackets).

Next we wondered whether accuracy in survival prediction could be improved by a combination of T-UCRs. In this respect, the significant 28 T-UCRs were categorized as up- or down-expressed in each NB sample according to their respective cut-off values identified by ROC curves. ROC and Kaplan-Meier survival analyses (Figure 1) showed that at least 15 up-regulated T-UCRs are needed to discriminate (*P *< 0.0001) long- from short-survivors at the highest sensitivity (94.12%) and specificity (94.12%). *MYCN *gene status was tested by Cox regression analysis and it was removed during the backward selection, showing that the multi-T-UCR expression signature is a *MYCN*-independent predictor for OS and EFS in our cohort. These findings endorse the identification of a novel candidate prognostic molecular signature based on the expression profile of the T-UCRs in high-risk NB patients.

### T-UCR signature and genomic regions involved in NB

On the basis of tumor genomic DNA availability, 29 (from 14 short- and 15 long-survivors) out of the 34 NBs were analyzed by array CGH (see additional file 1: Table S1; additional file 9: Figure S5). Tumors from the group of long-survivors showed 55 numerical (average 3.7 per subject) and 78 structural (average 5.2 per subject) chromosomal aberrations, mostly represented by 17q gain (60%) and 11q loss (40%). All tumors of short-survivors had at least one structural alteration (average 8.5 per subject). The most frequent changes were 17q gain (100%), 1p loss (57%), 2p gain (57%), 11q loss (43%), 1q gain (43%), and 3p loss (29%). Our results confirm that severe clinical courses are characterized by DNA changes at chromosomes 1, 2, 3, 7, 11, and 17 [5,6].

In order to investigate T-UCR alterations at genomic level, we associated the location of the 28 T-UCRs with the DNA imbalances identified in our cohort of samples. Fisher's test disclosed a significant *P*-value for UCRs mapping at 3q13.31 (*P *= 0.04), demonstrating that such UCRs (namely, uc.121 and uc.122) are more specifically associated with the corresponding genome location than expected by chance alone. According to this observation, one unfavorable NB (#1506) shows 3q11.2-q29 loss, whereas two tumors of long-survivors (#1889 and #2056) harbor 3q12.2-qter and 3q13.2-q23 gain, respectively. In this respect, we did not identify any significant T-UCR-enrichment within the more frequently observed regions of imbalance (i.e. 1p, 11q, 3p, 17q). In conclusion we hypothesize that the deregulation of T-UCR expression is not consistently associated with alterations at DNA level in the pathogenesis and progression of high-risk NB.

### Prediction of functional characterization of NB-associated T-UCRs

The presence of a positive correlation between expression levels of exonic UCRs and host genes would suggest changes in primary transcription rates. It is noteworthy that five UCRs (uc.322, uc.323, uc.421, uc.423, and uc.452) up-regulated in long-survivors are located within three genes (*SOX6*, *ZNF521*, *TSHZ3*) that we found significantly (at a false discovery rate of 0.01) over-expressed in long-surviving subjects of an unrelated cohort of 76 high-risk NBs (personal microarray data, manuscript in preparation). According to these results, we speculate a positive enhancer activity on mRNA transcription for some T-UCRs in NB, in agreement with previous observations [21,22].

**Figure 2:**
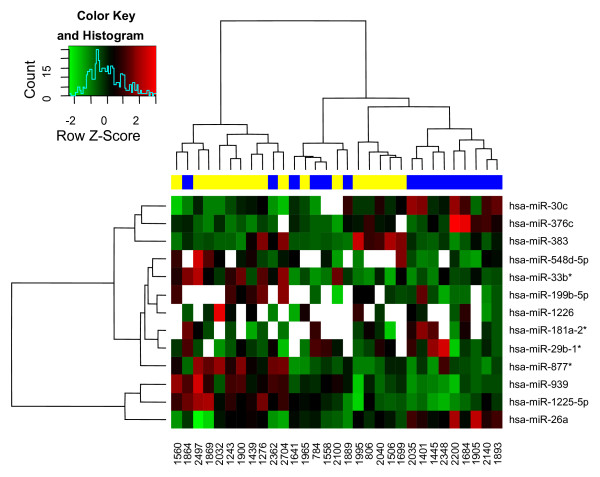
**Hierarchically clustered heat map of differentially expressed microRNAs (n = 13) between the two tumors conditions**. Non-detected signals are indicated in white. On top bar, short-survivors tumors (yellow) and long-survivor tumors (blue) are shown.

In order to investigate the post-transcriptional regulation of T-UCRs, we performed a genome-wide microRNA expression analysis in 32 out of 34 samples investigated for T-UCR levels. The expression dataset, after filtering, condensing by replicates average and control spots removal, contained 319 microRNAs and 31 eligible samples. Thirteen of the 319 (4.1%) microRNAs showed differential expression between the two categories of tumors (Figure 2; additional file 10: Figure S6). Interestingly, *ALK *tyrosine kinase receptor precursor mRNA is a putative target of hsa-miR-376c that is up-regulated in tumors of long-survivors. Although the targeting of the *ALK *3'-untranslated region by this microRNA remains to be experimentally validated, if true, this would suggest the importance for hsa-miR-376c to be induced in order to negate the effects of *ALK *amplification in advanced NBs [23].

Intriguingly, 4 microRNAs down-regulated in tumors of long-survivors (hsa-miR-383, hsa-miR-548d-5p, hsa-miR-939, hsa-miR-877*) target 3 genes implicated in neuronal differentiation (*CHD5*, *FYN *and *NTRK1*), that were recently reported to be over-expressed in low-risk tumors [7].

Since Calin et al. [10] demonstrated a negative regulation of T-UCRs by direct interaction with microRNAs, we argue that down-expression of the T-UCR signature in short-survivors may be due to the up-regulation of their own putative complementary microRNAs. Intriguingly, microRNA expression data confirmed our hypothesis: 5 out of the 13 microRNAs from the signature display their expression level that are negatively correlated with expression of 9 complementary UCRs able to differentiate between the two NB prognosis groups (Table 1). Sequence homologies give rise to 10 possible pairs, in agreement with previous reports on complex redundancy in regulatory mechanisms between microRNAs and T-UCRs [10].

**Table 1:** T-UCRs whose expression inversely correlates with complementary microRNAs differentially expressed in NB patients.

T-UCR	T-UCR expression in long- versus short-survivors	Complementary mature microRNA	microRNA expression in long- versus short-survivors
uc.209	up-regulated	hsa-miR-877*	down-regulated
uc.271	up-regulated	hsa-miR-383	down-regulated
uc.312	up-regulated	hsa-miR-877* hsa-miR-548d-5p	down-regulated
uc.330	up-regulated	hsa-miR-548d-5p	down-regulated
uc.371	up-regulated	hsa-miR-877*	down-regulated
uc.411	up-regulated	hsa-miR-33b*	down-regulated
uc.421	up-regulated	hsa-miR-877*	down-regulated
uc.435	up-regulated	hsa-miR-939	down-regulated
uc.452	up-regulated	hsa-miR-383	down-regulated

Moreover, we speculate that a further plausible explanation for the directionality of expression of T-UCRs in long- versus short-term survivors may be due to the contribution of an epigenetic regulation of T-UCR expression. This is supported by the fact that a CpG island methylator phenotype is present specifically in neuroblastoma with poor prognosis [24,25], and that epigenetic mechanisms not only regulate coding genes but also non-coding RNAs, such as miRNAs [26]. According to these information, we retrieved the promoter sequences of T-UCR-host genes from DataBase of Transcriptional Start Sites (DBTSS, release 7.0, http://dbtss.hgc.jp/), where each promoter sequence contains 1 kb upstream sequence and 200 bp sequence downstream from each transcription start site described. Then, we used the criteria and algorithm searcher at http://www.uscnorris.com/cpgislands2/cpg.aspx[27] to obtain the CpG-island information. We found that 29 out the 37 T-UCRs of the signature (78%) mapping within coding genes are associated with CpG islands in the promoter region of their own host genes (see additional file 11: Table S3). Therefore, like CpG island hypermethylation-mediated silencing of miRNAs with tumor suppressor features contribute to human cancer [28], we can argue that a global DNA hypermethylation events in unfavorable neuroblastomas may affect also T-UCR-host genes, and thus silence T-UCRs with a potential oncogenic role in metastatic NB tumors. Experimental validations will be performed in order to investigate the role of methylation in the regulation of T-UCR expression.

## Conclusions

Our findings strongly suggest that a deregulation of the microRNA/T-UCR network may play an important role in NB. Functional studies are mandatory to clarify coding and noncoding RNA interactions in the pathogenesis of this tumor, and could therefore identify potential therapeutic targets.

The potential to use this information as the first T-UCR prognostic signature for high-risk neuroblastoma patients must be tested in a larger, independent set of samples. Since immediate advantages of the qPCR strategy for T-UCR signature evaluation are speed, cost-effectiveness, minimal required amounts of tumor RNA, and applicability as routine test, this pilot study should put the basis for future analyses in order to investigate i.e., whether the T-UCR signature described here is a sensitive and specific genomic feature also in low- and intermediate-risk tumors.

## Abbreviations

(T-UCRs): Transcribed-Ultra Conserved Regions; PCR (RT-qPCR): reverse transcription-quantitative real-time; (NB): neuroblastoma; (Cq): quantification cycle; (MIQE): Minimum Information for Publication of Quantitative Real-Time PCR Experiments; (ROC): Receiver Operating Characteristic; (OS): Overall survival; (EFS): Event free survival; (CGH): Comparative Genomic Hybridization; (GEO): Gene Expression Omnibus; (DBTSS): DataBase of Transcriptional Start Sites.

## Competing interests

The authors declare that they have no competing interests.

## Authors' contributions

PS developed the study design; supervised the preparation and quality assessment of RNAs, and the microarray experiments; participated in performing RT-qPCR reactions and data analysis; drafted the manuscript. SS carried out the preparation of RNAs, the quantification and assessment of transcript integrity, and the microarray experiments; participated in performing the RT-qPCR reactions. SC, FV, CDV performed the T-UCR qPCR reactions. SM performed microarray analysis. AG provided patients' clinical data. SB participated in data analysis and critical revision of the manuscript. GPT participated in study conception and critical revision of the manuscript. All authors read and approved the final manuscript.

## Pre-publication history

The pre-publication history for this paper can be accessed here:

http://www.biomedcentral.com/1471-2407/9/441/prepub

## Supplementary Material

Additional file 1 **Table S1**. Clinical and biological characteristic of 34 NBs enrolled in the study. Click here for file

Additional file 2 **Raw Cq data**. Raw Cq values for each qPCR reaction, according to the Real-time PCR Data Markup Language (RDML). Click here for file

Additional file 3 **MIQE checklist**. Checklist according to the Minimum Information for Publication of Quantitative Real-Time PCR Experiments. Click here for file

Additional file 4 **Table S2**. T-UCRs differentially expressed between short- and long-survivors (P < 0.05). The table shows also results of ROC and survival analysis based on expression values of the 54 T-UCRs. Click here for file

Additional file 5 **Figure S1**. Box-and-Whisker plots of the comparison among values of T-UCR expression by qPCR (normalized to 18S rRNA, and after logarithmic transformation of original measures) between long- and short-survivors. Each box represents the values from the 25^th ^to 75^th ^percentile, the middle line represents the median, and a line extends from the minimum to the maximum value, excluding outliers which are displayed as blue dots. Nearby each outlier value is indicated the ID of the corresponding NB sample. Relative expression data were multiplied by 10^4^. Click here for file

Additional file 6 **Figure S2**. ROC curves based on qPCR results of the 54 T-UCRs differently expressed between long- and short-survivors. In each graph the point indicated with a red dot is the value corresponding to the highest average of sensitivity and specificity. Click here for file

Additional file 7 **Figure S3**. Kaplan-Meier overall and event-free survival curves of the first set of 20 NB patients categorized according to T-UCR expression values (above or below the threshold defined by the respective ROC curve). In each graph are reported the number of patients in predicted subgroups and the number of patients with event (between brackets). Click here for file

Additional file 8 **Figure S4**. Kaplan-Meier overall and event-free survival curves of the second set of 14 NB patients categorized according to T-UCR expression values (above or below the threshold defined in the first set of 20 NB samples by the respective ROC curve). In each graph are reported the number of patients in predicted subgroups and the number of patients with event (between brackets). Click here for file

Additional file 9 **Figure S5**. Structural chromosomal aberrations. The Figure shows percentages of gains (**A**) and losses (**B**) for each of the autosomal chromosomes detected by array CGH in tumors of 14 short- and 15 long-survivors. Click here for file

Additional file 10 **Figure S6**. Box plots of log2-transformed expression measures of the 13 microRNA differently expressed between long- and short-survivors. Each box represents the distribution of expression measured for "long-survivors" (blue, labeled by 0) and "short-survivors" (red, labeled by 1) tumor-category. Click here for file

Additional file 11 **Table S3**. T-UCRs associated with CpG islands in the promoter region of their own host genes. Click here for file
