# Supplementary material for: Transcribed-ultra conserved region expression is associated with outcome in high-risk neuroblastoma
Source: BMC Cancer. 2009 Dec 15;9:441. doi: 10.1186/1471-2407-9-441 (PMC2804711; doi:10.1186/1471-2407-9-441)
Supplement: Additional file 1 — Table S1. Clinical and biological characteristic of 34 NBs enrolled in the study. [file 1471-2407-9-441-S1.PDF]

Table S1. Clinical and biological characteristic of 34 NBs enrolled in the study.

Clinical data							Biological data		
**ID**	**Year of diagnosis**	**Age at diagnosis (months)**	**Type of relapse**	**Follow-up**	**OS * (months)**	**EFS** (months)**	***MYCN* status [‡]**	**gains [††]**	**losses [††]**
789	1990	21	---	CR [§]	164	164	not amplified	19	Xq21.1-qter
806	1990	34	disseminated	DWD[†]	6	5	not amplified	1q21.2-qter; 3p14.3-p14.1; 7p12.3-pter; 7q11.21-qter; 12q23.2-qter; 17q; 19; 22	1p36.21-pter; 3p14.3-pter; 4p; 11q13.4-qter; 16p12.3-pter
**1243**	**1995**	**47**	**local and disseminated**	**DWD**	**20**	**15**	**not amplified**	**1q21.1-qter; 11q12.3-q13.4; 12; 16; 17q21.31-qter**	**1p12-pter; 3p14.3-pter; 4q35.1-qter; 8; 11q13.4-qter; 15; 17q21.31-pter; 18; 19p13.11-p13.3; X**
1276	1996	68	disseminated	DWD	24	14	amplified	2p24.2-p24.3; 12q24.31; 14q22.2-qter; 17q11.2-qter; 18q21.2-qter	1p34.3-pter; 10q21.3-qter; 19
**1401**	**1997**	**14**	**---**	**CR**	**67**	**67**	**not amplified**	**1q21.1-q42.2; 2p16.4-pter; 17q12-qter; 22**	**9p21.3; 14q24.3-qter; 19p13.12-pter**
**1439**	**1997**	**38**	**disseminated**	**DWD**	**9**	**5**	**gain**	**1q21.1-qter; 2p16.3-pter; 7; 11q13.4-pter; 12q23.1-qter; 17q21.31-qter**	**3p24.1-pter; 11q13.4-qter; Y**
1445	1998	28	---	CR	121	121	not amplified	17	15q11.2
**1506**	**1998**	**50**	**disseminated**	**DWD**	**30**	**22**	**not amplified**	**1q21.2-q42.2; 2p24.3-pter; 7; 8p23.2-pter; 11q13.4-pter; 12q24.11-qter; 17q12-qter; 19p13.3-pter, Xp21.3-pter**	**3q11.2-q29; 11q13.4-qter; 15q25.2-qter; 19q13.32-qter**
1558	1998	50	local	CR	112	22	amplified	2p14-pter; 17q21.32-qter; 19; 22	1p22.2-pter; 10q11.21-qter
1560	1998	26	progression	DWD	12	12	amplified	2p24.3; 17q21.2-qter; 19	
1641	1999	33	disseminated	CR	104	37	gain	2p16.3-pter; 2q22.2-qter; 10q26.13-qter; 12q21.1-qter; 17q11.2-qter	1p36.12-pter; 3p14.3-pter; 8q24.23-qter; 11q14.3-qter
1684	1999	15	---	CR	101	101	not amplified	2; 2p24.3; 4; 6; 7; 8; 12; 13q12.11-q14.11; 17; 18; 20; 22	
1699	1999	45	disseminated	DWD	16	13	amplified	2p16.3-pter; 5q31.1-qter; 17q21.31-qter	1p34.2-pter; 3p21.31-pter; 11q22.3-qter; 14q23.3-qter; 17q21.31-pter
1768	2000	35	disseminated	DWD	17	14	not amplified	17q12-qter; 19	
1864	2000	48	---	CR	43	43	not amplified	Yq11.21-q11.223	19
**1869**	**2001**	**48**	**progression**	**DWD**	**1**	**1**	**amplified**	**2p14-pter; 17q21.32-qter; 19; 22**	**1p22.2-pter; 10q11.21-qter**
1889	2001	36	---	CR	82	82	gain	1q25.2-qter; 2p24.2-pter; 3q12.2-qter; 7; 17q12-qter; 18	2q31.1-q33.2; 3q12.2-pter; 4p12-pter; 8q23.3-qter; 11q13.4-qter; 15q11.2-q25.1; Xq21.1-q21.31; Y
**1893**	**2001**	**39**	**---**	**CR**	**45**	**45**	**not amplified**	**1q21.2-qter; 7; 11q13.4-14.1; 12; 17q12-qter**	**3p21.1-pter; 4p13-pter; 8q12.2-q21.13; 10p13-qter; 11q14.1-qter**
**1900**	**2001**	**37**	**disseminated**	**DWD**	**24**	**21**	**not amplified**	**6; 7; 11q13.4-pter; 14q32.33; 16q22.1-qter; 17; 17q21.31-qter; 18; 19; 20p11.23-qter; 21q22.3**	**11q13.4-qter; 20p11.23-pter**
1905	2001	15	---	CR	80	80	gain	2p22.3-pter; 12q24.21-qter; 16p13.11-pter; 17q21.32-qter; 22q11.21-q11.22	1p31.3-pter; 3p21.31-pter; 4p12-pter; 14q22.1-qter; 19p13.13
1965	2001	83	disseminated	DWD	34	29	not amplified	5; 6, 17q11.2-qter; 22	3; 4p15.31-pter; 9p13.3-pter; 11, 14q22.1-qter; 15, 16q22.3-qter; 19q13.2-qter; X
1995	2001	22	disseminated	DWD	21	14	not amplified	1p34.3-qter; 2; 5p15.33; 5q33.1; 5q35.1-qter; 6, 7; 8; 12; 13; 14q32.2-qter; 17q11.2-qter; 20; 21; 22	1p34.3-pter; 11p13-p14.3; 17q11.2-pter
**2032**	**2002**	**41**	**local and disseminated**	**DWD**	**17**	**12**	**gain**	**n.d.**	**n.d.**
**2035**	**2002**	**17**	**---**	**CR**	**66**	**66**	**not amplified**	**7; 12; 17; 17q; 18**	**3; 4; 9; 11q14.1-qter; 19; 21; 22; X, Y**
**2040**	**2002**	**35**	**local**	**DWD**	**21**	**20**	**not amplified**	**n.d.**	**n.d.**
2056	2002	46	---	AWSD [‖]	76	70	gain	1p31.1-p32.3; 1p22.2-q42.2; 2p16.1-pter; 3p26.1-pter; 3q13.2-q23; 6p21.2-pter; 7; 8; 9; 11q14.1-pter; 12; 13; 14; 17; 18; 20; 22q11.21-q11.22	21q21.2-q21.3
2100	2002	27	disseminated	DWD	11	10	gain	1q31.1-qter; 2p16.1-pter; 7q11.21-qter; 17q12-qter	1p22.1-pter; 4p12-pter; 10p12.1-pter; 19p13.13-pter; 21q22.3-qter; 22q12.1-qter; Y
2140	2002	55	---	CR	62	62	gain	1q21.1-qter; 2p24.3-pter; 7; 12q21.1-qter; 17; 17q12-qter	3p14.1-pter; 4p13-p16.1; 4q34.1-qter; 10q26.2-qter; 11q14.2-qter; Y
**2200**	**2003**	**125**	**---**	**CR**	**48**	**48**	**not amplified**	**n.d.**	**n.d.**
**2348**	**2004**	**40**	**---**	**CR**	**40**	**40**	**not amplified**	**n.d.**	**n.d.**
2362	2004	23	---	CR	43	43	not amplified	2; 6; 7; 8; 13; 17	3; 9; 10; 11q14.1-qter; 14q23.1-qter; 21q23; X
2425	2004	32	---	CR	39	39	amplified	2p24.3; 17q21.2-qter	1p21.3-pter; 6q27; 8q12.1-q13.1
**2497**	**2004**	**48**	**local and disseminated**	**DWD**	**35**	**14**	**gain**	**n.d.**	**n.d.**
**2704**	**2006**	**13**	**local**	**DWD**	**7**	**4**	**amplified**	**2p24.3; 2p11.3-p14; 17q11.2-qter; 20p12.3-pter; 22q12.3-qter**	**1p13.3-pter; 9; 10; 14q13.2-qter, 21**

Bold font: NBs included in the 2nd set of 14 samples. *OS: Overall survival; **EFS: Event-Free Survival; [§] CR: complete remission; [†] DWD: Death with disease; [‖]AWSD: alive with stable disease; [‡] *MYCN* status by Fluorescence In Situ Hybridization (FISH) analysis; [††] Array CGH results; n.d.: not done
