# Supplementary material for: Transcribed-ultra conserved region expression is associated with outcome in high-risk neuroblastoma
Source: BMC Cancer. 2009 Dec 15;9:441. doi: 10.1186/1471-2407-9-441 (PMC2804711; doi:10.1186/1471-2407-9-441)
Supplement: Additional file 3 — MIQE checklist. Checklist according to the Minimum Information for Publication of Quantitative Real-Time PCR Experiments. [file 1471-2407-9-441-S3.PDF]

Item to check	Importance	
**Experimental design**		
Definition of experimental and control groups	E	EXPERIMENTAL SAMPLES: RNAs from 34 high-risk, stage 4 neuroblastoma patients (789, 806, 1243, 1276, 1401, 1439, 1445, 1506, 1558, 1560, 1641, 1684, 1699, 1768, 1864, 1869, 1889, 1893, 1900, 1905, 1965, 1995, 2032, 2035, 2040, 2056, 2100, 2140, 2200, 2348, 2362, 2425, 2497, 2704)
Number within each group	E	A total of 34 experimental samples have been enrolled in the study
Assay carried out by core lab or investigator's lab?	D	Assays were carried out by investigator's lab
Acknowledgement of authors' contributions	D	
**Sample**		
Description	E	Neuroblastoma tumors have been collected at the onset of disease. All tumor samples were classified as Schwannian stroma-poor neuroblastoma according to the International Neuroblastoma Pathology Committee (Shimada H et al. Terminology and morphologic criteria of neuroblastic tumors: recommendations by the International Neuroblastoma Pathology Committee. Cancer. 1999;86:349-363) with at least 80% of neuroblasts.
Volume/mass of sample processed	D	
Microdissection or macrodissection	E	Macrodissected
Processing procedure	E	Neuroblastoma tumors have been collected at the onset of disease. Each surgically resected tumor was processed by pathologists and immediately stored at –80°C.
If frozen - how and how quickly?	E	Neuroblastoma tumors have been collected at the onset of disease. Each surgically resected tumor was processed by pathologists and immediately stored at –80°C.
If fixed - with what, how quickly?	E	Not fixed
Sample storage conditions and duration (especially for FFPE samples)	E	Samples were stored at –80°C
**Nucleic acid extraction**		
Procedure and/or instrumentation	E	Total RNA was extracted by using the PerfectPure™ RNA Tissue Kit (5Prime, Hamburg, Germany), following manufacture's protocol. Homogenization of samples was performed by using the Tissue Lyser (Qiagen GmbH, Germany) according to manufacture's instructions. Briefly, after adding Lysis solution to the cell pellet, the pellet was dislodged from the bottom of the tube by using a pipet tip. Sample was vortex vigorously to resuspend the pellet until there were no visible cell clumps in the lysate. Centrifugation steps were performed by using Centrifuge 5424 (Eppendorf AG, Hamburg, Germany).<br>PRINCIPLE: Total RNA is purified by first adding cultured cells to a detergent/salt solution (a chaotropic guanidium isothiocyanate solution) to lyse the cells and eliminate endogenous RNase activity. Lysis and homogenization disrupts the cell membranes releasing RNA into the lysing solution, and shears the genomic DNA, decreasing the viscosity of the lysate. Next, the lysates are applied to the purification column to bind the RNA and wash away proteins, DNA, and other contaminants. Residual DNA is removed by on-column DNase tratment, and the DNA fragments and DNase are removed by subsequent washing steps. Finally, the purified RNA is eluted with DEPC-treated water.
Name of kit and details of any modifications	E	PerfectPure™ RNA Tissue Kit (5Prime, Hamburg, Germany). We exactly followed manufacture's protocol. The only modification was in the Lysis step: the incubation time was extended for 30 minutes.
Source of additional reagents used	D	2-mercaptoethanol 98% (Acros Organics, NJ, USA); Absolute ethanol (Carlo Erba Reactifs, France); RNase-free Dnase I (Ambion, TX, USA)
Details of DNase or RNase treatment	E	On column RNase-free DNase I treatment: after Wash 1, 50 µl of DNase solution (25 Units of DNase in 10X DNase I Buffer) were added to the Purification Column and incubated at room temperature for 20 minutes. Then 200 µl DNase Wash Solution (component of the PerfectPure kit) were added to the column. The column was centrifuged at 13,000 x g for 1 minute in a Centrifuge 5424 (Eppendorf AG, Hamburg, Germany). Additional 200 µl DNase Wash Solution were added and the colum was centrifuged at 13,000 x g for 2 minutes. The column was transfer to a new collection tube and we proceed with Wash 2 and elutions as specified in the protocol.
Contamination assessment (DNA or RNA)	E	No-reverse transcription controls was used to assess absence of DNA for both RNA targets. With this purpose, amplification of 18S rRNA was performed in RNAs isolated from LAN-5 and GI-ME-N cells. We used VIC-labeled TaqMan Gene Expression assay (Applied Biosystems, Foster City, CA) in a total volume of 10 µl, containing 10 ng of RNA, 2.5x RealMaster Mix Probe (5Prime, Hamburg, Germany) and 20x primer/probe mix. Reactions were setup in Real-time white tubes (Eppendorf, Hamburg, Germany) and they were run on the Mastercycler® epRealPlex4 S system (Eppendorf). Cycling conditions were as follows: 95°C for 2 minutes, 40 cycles at 95°C for 15 seconds and at 60°C for 1 minutes. The signal of the amplification plot was very late (Cq>40).
Nucleic acid quantification	E	RNA quantification was assessed by using microfluidic analysis (Agilent Technologies' Bioanalyzer).
Instrument and method	E	Total RNA and small RNAs were quantified by RNA 6000 Nano® and Small RNA® assays, respectively, on the 2100 Bioanalyzer (Agilent Technologies, Santa Clara, CA), following manufacture's protocols.
Purity (A260/A280)	D	
Yield	D	
RNA integrity method/instrument	E	2100 Bioanalyzer (Agilent Technologies, Santa Clara, CA)
RIN/RQI or Cq of 3' and 5' transcripts	E	789: RIN=7.8; 806: RIN=8.0; 1243: RIN=6.5; 1276: Ratio [28S/18S]=1.8; 1401: RIN=7.8; 1439: RIN=6.5; 1445: RIN=6.5; 1506: RIN=6.5; 1558: RIN=6.9; 1560: RIN=6.5; 1641: RIN=7.4; 1684: RIN=6.5; 1699: RIN=6.5; 1768: RIN=6.5; 1864: RIN=6.6; 1869:Ratio [28S/18S]=1.8; 1889: Ratio [28S/18S]=1.8; 1893: RIN=7.7; 1900: RIN=6.5; 1905: RIN=6.5; 1965: RIN=6.8; 1995: RIN=8.7; 2032: RIN=6.4; 2035: RIN=8.1; 2040: Ratio [28S/18S]=1.8; 2056: Ratio [28S/18S]=1.8; 2100: RIN=6.7; 2140: Ratio [28S/18S]=1.7; 2200: RIN=8.3; 2348: RIN=8.4; 2362: RIN=6.0; 2425: RIN=6; 2497: RIN=7.0; 2704: RIN=7.2
Electrophoresis traces	D	Not performed
Inhibition testing (Cq dilutions, spike or other)	E	Not performed by using dilutions of samples or universal inhibition assays. The amplification product for the reference gene assay obtained in each cDNA target has been considered sufficient to rule out the presence of inhibitors of reverse-transcription activity or PCR, also taking into account the high quality of starting RNAs.
**Reverse transcription**		
Complete reaction conditions	E	RNA was amplified and reverse transcribed by the WT-Ovation™ RNA Amplification System kit (NuGEN Technologies, San Carlos, CA) following manufacture's protocol and using the Mastercycler® epGradient S (Eppendorf).
Amount of RNA and reaction volume	E	Amount of RNA: 50 ng; Reaction volume: 42 µl
Priming oligonucleotide (if using GSP) and concentration	E	
Reverse transcriptase and concentration	E	WT-Ovation™ RNA Amplification System kit (NuGEN Technologies, San Carlos, CA) components
Temperature and time	E	Primer annealing. 65°C for 5 minutes; First strand synthesis: 4°C for 1 minute, 25°C for 10 minutes, 42°C for 10 minutes, 70°C for 15 minutes; Second strand synthesis: 4°C for 1 minute, 25°C for 10 minutes, 50°C for 30 minutes, 70°C for 5 minutes; Post second strand enhancement: 4°C for 1 minute, 37°C for 15 minutes, 80°C for 20 minutes; SPIA™ amplification: 4°C for 1 minute, 47°C for 60 minutes, 95°C for 5 minutes.
Manufacturer of reagents and catalogue numbers	D	
Cqs with and without RT	D*	Samples have been validated as DNA free by performing a no–reverse transcription control when first extracting RNA.
Storage conditions of cDNA	D	-20°C
**qPCR target information**		
If multiplex, efficiency and LOD of each assay	E	Not applicable since we performed monoplex qPCRs
Sequence accession number	E	T-UCR assays (Catalogue no: 'T-UCR-all', Primer Design Ltd): see "UCR sequences" below. Data from: Bejerano G, Pheasant M, Makunin I, Stephen S, Kent WJ, Mattick JS, Haussler D. Ultraconserved Elements in the Human Genome. Science, 304(5675), pp. 1321-1325 (2004).<br>Eukaryotic 18S rRNA TaqMan® Gene Expression Assay (P/N 4319413E, Applied Biosystems): X03205.1
Location of amplicon	D	

Amplicon length	E	T-UCR assays (Catalogue no: 'T-UCR-all', Primer Design Ltd): not available due to intellectual property of Primer Design Ltd. Approximately 200 bases<br>Eukaryotic 18S rRNA TaqMan® Gene Expression Assay (P/N 4319413E, Applied Biosystems): 187 bases
In silico specificity screen (BLAST, etc)	E	Primers for specific detection of T-UCRs were supplied pre-optimized by PrimerDesign Ltd, UK. Each T-UCR assay was individually validated and shown to be 100% specific and close to 100% efficient.<br>Primers for specific detection of 18S rRNA were supplied pre-optimized by Applied Biosystems, Foster City, CA. All TaqMan® Gene Expression Assays have been designed through Applied Biosystems' validated bioinformatics pipeline.
Pseudogenes, retropseudogenes or other homologs?	D	
Sequence alignment	D	
Secondary structure analysis of amplicon	D	
Location of each primer by exon or intron (if applicable)	E	Not applicable for T-UCR assays<br>Eukaryotic 18S rRNA assay location: 609
What splice variants are targeted?	E	Not applicable
qPCR oligonucleotides		
Primer sequences	E	Not available due to intellectual property of Primer Design Ltd and Applied Biosystems
RTPrimerDB identification number	D	
Probe sequences	D**	Not applicable for T-UCR assays (SYBR® green detection chemistry)<br>18S rRNA: not available due to intellectual property of Applied Biosystems
Location and identity of any modifications	E	T-UCR assays: no modifications (SYBR® green detection chemistry)<br>18S rRNA: VIC™ dye-labeled TaqMan® MGB probe
Manufacturer of oligonucleotides	D	PrimerDesign Ltd, Hants, UK
Purification method	D	
qPCR protocol		
Complete reaction conditions	E	cDNA products were then diluted 1:25 in molecular biology grade water. T-UCRs were quantified by Transcribed Ultra Conserved Regions real-time PCR assays (PrimerDesign Ltd, Hants, UK) using SYBR® green detection chemistry. qPCR reactions were carried out in a total volume of 10 µl, containing 2 µl of diluted cDNA, 2.5x RealMaster Mix SYBR ROX (5Prime, Hamburg, Germany) and 150 nM of the specific T-UCR primer mix. Amplification of Eukaryotic 18S rRNA, used as reference gene, was performed by using VIC-labeled TaqMan Gene Expression assay (Applied Biosystems, Foster City, CA) in a total volume of 10 µl, containing 2 µl of diluted cDNA, 2.5x RealMaster Mix Probe (5Prime) and 20x primer/probe mix. Reactions were setup in 96-white-well Twin.tec® real-time plates (Eppendorf) by means of EpMotion 5070 Liquid Handling Workstation (Eppendorf, Hamburg, Germany). All reactions were performed in duplicate on the Mastercycler® epRealPlex4 S system (Eppendorf). Cycling conditions were as follows: 95°C for 2 minutes, 40 cycles at 95°C for 15 seconds and at 60°C for 1 minutes, followed by a melting curve (ramping from 60°C to 95°C in 20 minutes) to ensure the presence of the specific amplicon.
Reaction volume and amount of cDNA/DNA	E	Reaction volume: 10 µl; Amount of cDNA: 10 ng
Primer, (probe), Mg++ and dNTP concentrations	E	T-UCR primer mix: 150 nM/ 10 µl reaction; 18S rRNA primers: 900 nM each in the 20x pre-formulated mix; 18S rRNA hydrolysis probe: 250 nM in the 20x pre-formulated mix; 12.5 mM Magnesium acetate; 1.0 mM dNTPs with dUTP in the 2.5x masterMix
Polymerase identity and concentration	E	HotMaster Taq DNA Polymerase (5Prime, Hamburg, Germany). Polymerase concentration: 0.1 U/µl in the 2.5x MasterMix
Buffer/kit identity and manufacturer	E	2.5x RealMaster Mix SYBR ROX for T-UCR assays / 2.5x RealMaster Mix Probe for 18S rRNA assay (5Prime, Hamburg, Germany).
Exact chemical constitution of the buffer	D	
Additives (SYBR Green I, DMSO, etc.)	E	T-UCR assays: SYBR Green I (5Prime, Hamburg, Germany)
Manufacturer of plates/tubes and catalog number	D	96-white-well Twin.tec® real-time plates (Eppendorf, Hamburg, Germany, catalog number: 951022055) with heat sealing films (Eppendorf, Hamburg, Germany, catalog number: 951023060)
Complete thermocycling parameters	E	95°C for 2 minutes, 40 cycles at 95°C for 15 seconds and at 60°C for 1 minutes
Reaction setup (manual/robotic)	D	Reactions were setup by means of EpMotion 5070 Liquid Handling Workstation (Eppendorf, Hamburg, Germany)
Manufacturer of qPCR instrument	E	Mastercycler® epRealPlex4 S system (Eppendorf, Hamburg, Germany).
qPCR validation		
Evidence of optimisation (from gradients)	D	
Specificity (gel, sequence, melt, or digest)	E	T-UCR assays: melting curve analysis (ramping from 60°C to 95°C in 20 minutes. Fluorescence data are measured continously).<br>Predicted and measured melting temperature values are provided as Supplementary data.
For SYBR Green I, Cq of the NCT	E	The signal of the amplification plot was very late (Cq>35) and therefore there was a very high Cq value difference between the negative control and all the cDNA sample results.
Standard curves with slope and y-intercept	E	Primers for specific detection of T-UCRs were supplied pre-optimized by PrimerDesign Ltd, UK. Each T-UCR assay was individually validated and shown to be 100% specific and close to 100% efficient.<br>Primers/probe for specific detection of Eukaryotic 18S rRNA were supplied pre-optimized by Applied Biosystems, Foster City, CA. TaqMan® Gene Expression Assays were used as the Gold Standard in the MicroArray Quality Control (MAQC) Project, which compared data from seven microarray platforms (Nature Biotechnology, September 2006). TaqMan® Assays have the highest specificity, highest sensitivity and the largest dynamic range of any gene expression technology.
PCR efficiency calculated from slope	E	
Confidence interval for PCR efficiency or standard error	D	
r2 of standard curve	E	
Linear dynamic range	E	
Cq variation at lower limit	E	
Confidence intervals throughout range	D	
Evidence for limit of detection	E	
If multiplex, efficiency and LOD of each assay	E	Not applicable since we performed monoplex qPCRs
Data analysis		
qPCR analysis program (source, version)	E	RealPlex software v. 2.0 (Eppendorf, Hamburg, Germany)
Cq method determination	E	The threshold is used to specify Cq values of samples. The Cq value is the cycle in which the fluorescence signal intersects with the threshold. The threshold is determined using the Noiseband method: the threshold is specified so that it is significantly (10 times the standard deviation) above the noise of the baseline. The baseline is automatically calculated for every sample individually.
Outlier identification and disposition	E	Runs were performed in duplicate as instrument and liquid handling variations were shown to be minimal (mean SD for non-amplified RNA: GI-ME-N= 0.28, LAN-5= 0.26; mean SD for pre-amplified RNA:GI-ME-N= 0.12, LAN-5= 0.12). Single runs were excluded when the melting curve analysis revealed unintended amplification products: melting curves with more than one peak, or one single peak but with a melt temperature different from the expected one (calculated by PrimerDesign's design software, accounting salt conditions of the mastermix).
Results of NTCs	E	In each plate we excluded false positives and unintended amplification products (e.g., primer dimers) by performing melting curve analysis after each T-UCR amplification.
Justification of number and choice of reference genes	E	*18S* rRNA has been used as reference gene. This choice was based on:<br>i) Establishment of the optimal reference genes by the geNorm™ Housekeeping Gene Selection Kit and software (v. 3.4) (PrimerDesign Ltd, UK) using PerfectProbe™ detection chemistry. Briefly, we analyzed 10 stroma-poor neuroblastoma tumors to determine which control genes are most stable (among *GAPDH, EIF4A2, CYC1, YWHAZ, UBC, TOP1, SDHA, RPL13A, B2M, ATP5B, ACTB, 18S*). The output results showed that the *18S* is one of the 3 most stable genes for human neuroblastoma cell lines, together with *TOP1* and *CYC1*, with an average expression stability value M = 0.5.<br>Moreover, RT-qPCR tests performed in our laboratory confirmed that 18S rRNA level is invariant among a panel of neuroblastoma cell lines and neuroblastoma tumors. According to such selection, *18S* has been successfully used as reference genes in previous papers (i.e. Longo L. et al. Int. J. Oncology, 33: 985-91, 2008).<br>ii) The invariability of the *18S* rRNA assay among samples used in the present study is shown by the small difference of standard deviations for the Cq variance (≤0.12).
Description of normalisation method	E	Data normalization has been carried out against 18S rRNA as endogenous unregolated reference gene. For each cDNA, the duplicate T-UCR Cq values were averaged, and the normalized Cq was calculated by subtracting the mean Cq value for 18S rRNA from each T-UCR mean Cq value.
Number and concordance of biological replicates	D	
Number and stage (RT or qPCR) of technical replicates	E	qPCR reactions were performed in duplicate
Repeatability (intra-assay variation)	E	For each sample, standard deviation (SD) for the Cq variance between replicates has been used to express intra-assay variation. Instrument and liquid handling variations were shown to be minimal (SD <0.5).
Reproducibility (inter-assay variation, %CV)	D	
Power analysis	D	
Statistical methods for result significance	E	Student's t-test; Box-and-Whisker plots; Receiver Operating Characteristic (ROC) curves
Software (source, version)	E	RealPlex software v. 2.0 (Eppendorf, Hamburg, Germany); MedCalc® software v. 10.0.1.0 (Mariakerke, Belgium); Microsoft Office Excel 2003
Cq or raw data submission using RDML	D	Yes, in Supplementary data
