# Supplementary material for: Transcribed-ultra conserved region expression is associated with outcome in high-risk neuroblastoma
Source: BMC Cancer. 2009 Dec 15;9:441. doi: 10.1186/1471-2407-9-441 (PMC2804711; doi:10.1186/1471-2407-9-441)
Supplement: Additional file 4 — Table S2. T-UCRs differentially expressed between short- and long-survivors (P < 0.05). The table shows also results of ROC and survival analysis based on expression values of the 54 T-UCRs. [file 1471-2407-9-441-S4.PDF]

Table S2. T-UCRs differentially expressed between short- and long-survivors (P <0.05). The table shows also rsults of ROC and survival analysis based on expression values of the 54 T-UCRs

T-UCR	Type^	Band	Upstream gene†	Within gene†	Downstream gene†	*P* value‡	ROC analysis				Survival analysis*			Survival analysis**	
							P value	Sensitivity (%)	Specificity (%)	AUC§	Z statistic	OS‖ *P* value	EFS†† *P* value	OS‖ *P* value	EFS†† *P* value
uc.35	p	1p21.3	*PTBP2*	*DPYD*	*FLJ35409*	0.0323	0.0231	75.0	75.0	0.750	2.272	0.0280	0.1334	0.0072	0.0072
uc.51	n	2p16.1	*AB067499*	-	*VRK2*	0.0131	0.0012	100.0	58.3	0.812	3.237	0.0155	0.0229	0.0181	0.0357
uc.58	n	2p16.1	*FANCL*	-	*BCL11A*	0.0253	0.0001	100.0	83.3	0.896	5.491	0.0004	0.0004	0.4262	0.4262
uc.64	p	2p15	*TMEM17*	*NACSIN*	*OTX1*	0.0037	0.0001	100.0	66.7	0.885	5.093	0.0071	0.0222	0.0007	0.0007
uc.70	p	2q22.3	*KYNU*	*ARHGAP15*	*AK126774*	0.0111	0.0023	75.0	91.7	0.802	3.050	0.0018	0.0133	0.0049	< 0.0001
uc.81	n	2q22.3	*ZFHX1B*	-	*ACVR2*	0.0027	0.0001	75.0	91.7	0.880	4.913	0.0004	0.0040	0.0019	0.0004
uc.84	p	2q24.1	*NR4A2*	-	*GPD2*	0.0051	0.0006	75.0	91.7	0.823	3.437	0.0047	0.0133	0.5032	0.6580
uc.100	n	2q31.1	*RAPGEF4*	*ZAK*	*CDCA7*	0.0491	0.0006	87.5	83.3	0.823	3.437	0.0018	0.0029	0.4156	0.4156
uc.121	n	3q13.31	*ZBTB20*	-	*AF119886*	0.0004	0.0001	100.0	75.0	0.937	7.893	0.0021	0.0055	0.0007	0.0007
uc.122	n	3q13.31	*ZBTB20*	-	*AF119886*	0.0078	0.0001	87.5	75.0	0.844	3.889	0.0067	0.0125	0.0007	0.0007
uc.129	e	3q25.2	*TMEM14E*	*MBNL1*	*P2RY1*	0.0055	0.0012	75.0	91.7	0.812	3.237	0.0018	0.0133	0.0019	0.0004
uc.165	n	5q14.3	*TMEM161B*	-	*MEF2C*	0.0156	0.0067	62.5	91.7	0.781	2.711	0.0012	0.0005	0.0115	0.0070
uc.167	n	5q14.3	*MEF2C*	-	*CETN3*	0.0061	0.0001	62.5	100.0	0.865	4.428	< 0.0001	0.0001	0.0348	0.0348
uc.185	e	5q35.3	*COL23A1*	*CLK4*	*ZNF354A*	0.0060	0.0001	87.5	83.3	0.875	4.742	0.0018	0.0008	0.0021	0.0014
uc.189	e	6p21.31	*STK38*	*SFRS3*	*CDKN1A*	0.0054	0.0006	100.0	58.3	0.823	3.437	0.0185	0.0417	0.0007	0.0007
uc.192	n	6p12.3	*TFAP2B*	-	*PKHD1*	0.0022	0.0001	75.0	100.0	0.885	5.093	0.0001	0.0004	0.8038	0.8038
uc.196	n	6q16.1	*C6orf167*	-	*POU3F2*	0.0204	0.0023	100.0	58.3	0.802	3.050	0.0155	0.0275	0.7166	0.5731
uc.200	n	6q16.1	*C6orf167*	-	*POU3F2*	0.0288	0.0067	87.5	66.7	0.781	2.711	0.0083	0.0047	0.2032	0.1663
uc.209	e	7p15.3	*MGC27348*	*TRA2A*	*AB052759*	0.0030	0.0001	100.0	75.0	0.906	5.948	0.0021	0.0022	0.0039	0.0018
uc.210	p	7p15.2	*KIAA0087*	-	*SKAP2*	0.0164	0.0003	75.0	100.0	0.833	3.654	0.0001	0.0004	0.0008	0.0003
uc.211	n	7p15.2	*KIAA0087*	*SKAP2*	*HOXA1*	0.0113	0.0001	75.0	100.0	0.865	4.428	0.0086	0.0812	0.0181	0.0357
uc.215	n	7p14.1	*INHBA*	*GLI3*	*C7orf25*	0.0009	0.0001	100.0	83.3	0.917	6.481	0.0004	0.0008	0.0986	0.0237
uc.220	n	7q21.3	*SHFM1*	-	*DLX5*	0.0207	0.0159	62.5	91.7	0.760	2.411	0.0032	0.0093	0.7680	0.6544
uc.235	n	8p21.2	*EBF2*	*DOCK5*	*AK129682*	0.0014	0.0001	87.5	83.3	0.906	5.948	0.0018	0.0136	0.0816	0.1641
uc.236	n	8p12	*FKSG2*	-	*FLJ14299*	0.0115	0.0001	87.5	83.3	0.844	3.889	0.0005	0.0006	0.0898	0.2374
uc.254	n	9p21.3	*AK096011*	-	*ELAVL2*	0.0152	0.0012	100.0	75.0	0.812	3.237	0.0021	0.0002	0.1865	0.1585
uc.265	e	9q31.1	*ABCA1*	*SLC44A1*	*FSD1L*	0.0009	0.0001	87.5	83.3	0.906	5.948	0.0018	0.0209	0.0007	0.0007
uc.269	n	9q33.3	*CRB2*	*DENND1A*	*LHX2*	0.0118	0.0001	100.0	66.7	0.844	3.889	0.0033	0.0045	0.0162	0.0115
uc.271	p	9q33.3	*GAPVD1*	*MAPKAP1*	*LOC51145*	0.0095	0.0001	87.5	83.3	0.844	3.889	0.0034	0.0306	0.0297	0.0297
uc.290	e	10q22.3	*C10orf41*	*C10orf11*	*KCNMA1*	0.0264	0.0003	62.5	100.0	0.833	3.654	< 0.0001	0.0001	0.5032	0.6580
uc.291	p	10q22.3	*C10orf41*	*C10orf11*	*KCNMA1*	0.0225	0.0085	87.5	66.7	0.776	2.633	0.0369	0.0815	0.1051	0.0914
uc.307	p	10q24.32	*LBX1*	*BTRC*	*AF090931*	0.0061	0.0001	87.5	83.3	0.875	4.742	0.0013	0.0017	0.0008	0.0003
uc.312	p	10q26.11	*CASC2*	*C10orf84*	*PRLHR*	0.0057	0.0003	100.0	58.3	0.833	3.654	0.0155	0.0275	0.0021	0.0014
uc.317	n	10q26.3	*BC022565*	*MGMT*	*FLJ11370*	0.0040	0.0001	100.0	58.3	0.859	4.283	0.0185	0.0383	0.0208	0.0162
uc.321	n	11p15.2	*CALCB*	-	*SOX6*	0.0004	0.0001	100.0	75.0	0.937	7.893	0.0015	0.0193	0.4501	0.2636
uc.322	n	11p15.2	*CALCB*	*SOX6*	*AL136602*	0.0084	0.0001	100.0	90.9	0.966	11.029	< 0.0001	< 0.0001	0.0082	0.0082
uc.323	p	11p15.2	*AL136602*	*SOX6*	*MASP2*	0.0056	0.0001	100.0	66.7	0.865	4.428	0.0056	0.0701	0.0297	0.0297
uc.330	e	11q13.2	*CCS*	*RBM14*	*MGC15912*	0.0192	0.0012	100.0	66.7	0.812	3.237	0.0033	0.0045	0.7345	0.6585
uc.367	p	14q13.1	*AKAP6*	*NPAS3*	*EGLN3*	0.0237	0.0106	62.5	91.7	0.771	2.557	0.0021	0.0121	0.2653	0.0107
uc.371	n	14q13.2	*INSM2*	*GARNL1*	*GARNL1*	0.0144	0.0006	87.5	83.3	0.823	3.437	0.0012	0.0101	0.0208	0.0162
uc.374	p	14q13.3	*SLC25A21*	*MIPOL1*	*FOXA1*	0.0235	0.0001	75.0	83.3	0.854	4.146	0.0033	0.0073	0.9075	0.7723
uc.376	e	14q21.2	*FAM179B*	*PRPF39*	*FKBP3*	0.0241	0.0159	62.5	83.3	0.760	2.411	0.0014	0.0138	0.0019	0.0004
uc.391	e	15q23	*AF130086*	*MAP2K5*	*PIAS1*	0.0281	0.0106	87.5	66.7	0.771	2.557	0.0258	0.1880	0.0060	0.0010
uc.406	e	16q22.1	*CYB5B*	*NFAT5*	*NQO1*	0.0223	0.0001	87.5	83.3	0.927	7.116	0.0018	0.0008	0.0039	0.0018
uc.411	n	17q12	*LHX1*	*AATF*	*ACACA*	0.0470	0.0231	50.0	91.7	0.750	2.272	0.0042	0.0001	0.5856	0.5158
uc.421	n	18q11.2	*HRH4*	*ZNF521*	*SS18*	0.0367	0.0001	75.0	100.0	0.865	4.428	< 0.0001	< 0.0001	0.0297	0.0297
uc.423	n	18q11.2	*HRH4*	*ZNF521*	*SS18*	0.0169	0.0001	75.0	91.7	0.844	3.889	0.0018	0.0133	0.0399	0.0399
uc.435	n	18q21.2	*CCDC68*	*TCF4*	*LOC100132501*	0.0082	0.0001	87.5	75.0	0.844	3.889	0.0063	0.0491	0.0021	0.0014
uc.436	e	18q21.2	*CCDC68*	*TCF4*	*LOC100132501*	0.0327	0.0231	87.5	66.7	0.750	2.272	0.0122	0.0906	0.0661	0.0538
uc.444	n	19q12	*FKSG49*	*C19orf2*	*PRO1410*	0.0454	0.0003	87.5	75.0	0.833	3.654	0.0051	0.0098	0.0208	0.0162
uc.452	e	19q12	*AL137518*	*TSHZ3*	*ZNF507*	0.0318	0.0067	75.0	83.3	0.781	2.711	0.0080	0.0030	0.0208	0.0162
uc.467	n	14q13.1	*PCYT1B*	*POLA*	*ARX*	0.0119	0.0012	75.0	83.3	0.812	3.237	0.0033	0.0306	0.8497	0.6183
uc.469	p	Xp22.11	*POLA*	-	*ARX*	0.0380	0.0001	87.5	83.3	0.844	3.889	0.0018	0.0209	0.3486	0.5278
uc.477	e	Xq22.2	*MGC39655*	*PLP1*	*MGC39900*	0.0058	0.0001	100.0	66.7	0.875	4.742	0.0056	0.0066	0.4152	0.3247

^p=partial exonic, n=nonexonic, e=exonic; †Unigene ID; ‡Two-tailed Student's *t* -test; §AUC: Area Under Curve; * Data analysis of the 1st set of 20 samples; **Data analysis of the 2nd set of 14 samples; ‖OS: overall survival; ††EFS: event-free survival
