# Supplementary figures and images for: Transcribed-ultra conserved region expression is associated with outcome in high-risk neuroblastoma
Source: BMC Cancer. 2009 Dec 15;9:441. doi: 10.1186/1471-2407-9-441 (PMC2804711; doi:10.1186/1471-2407-9-441)

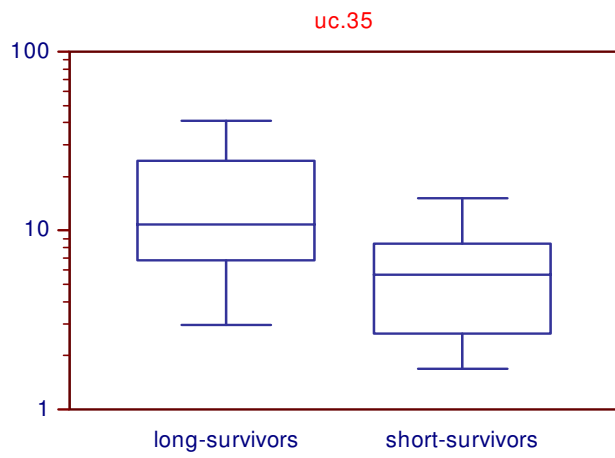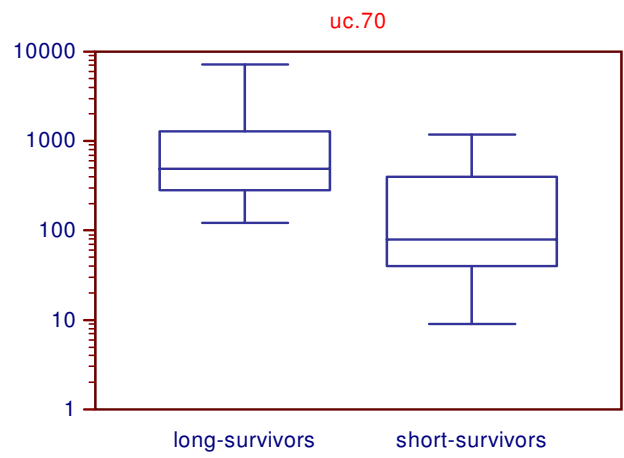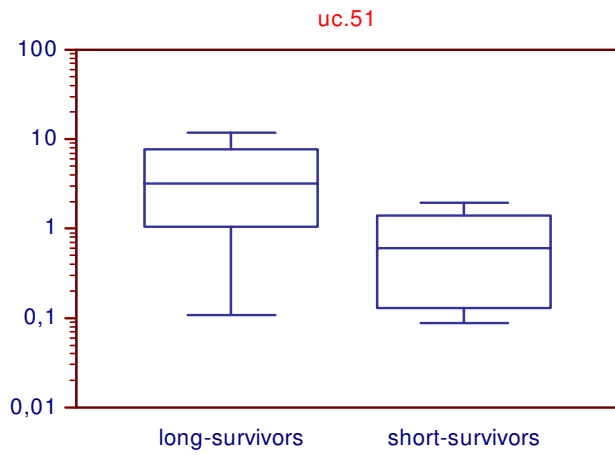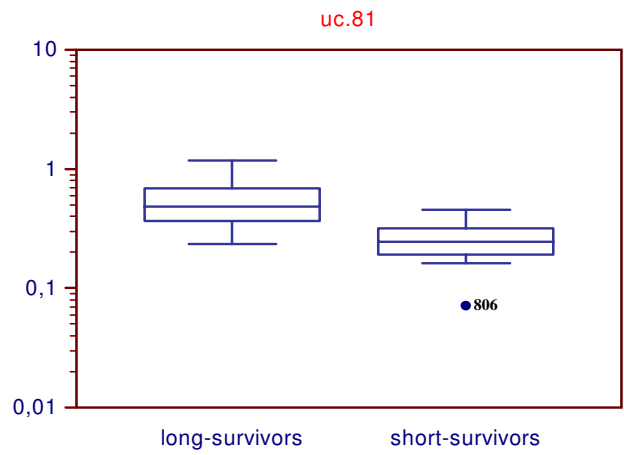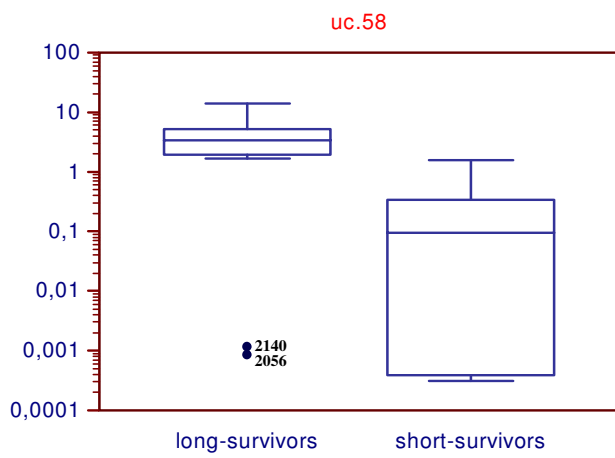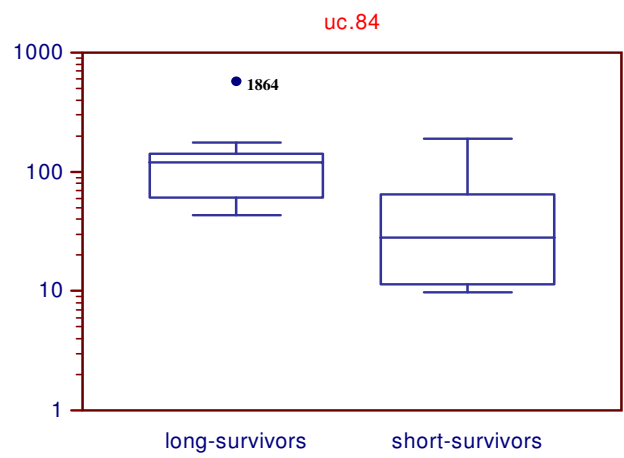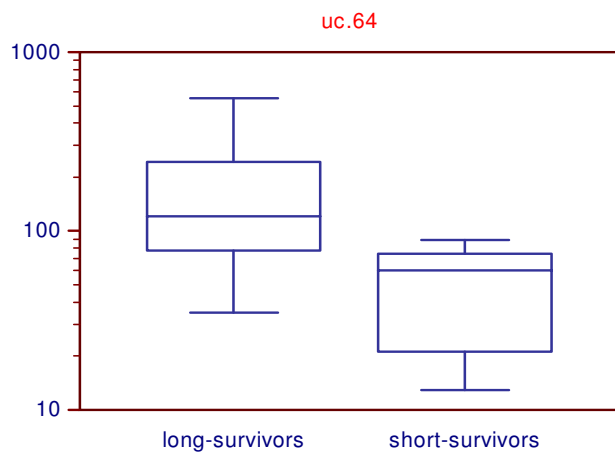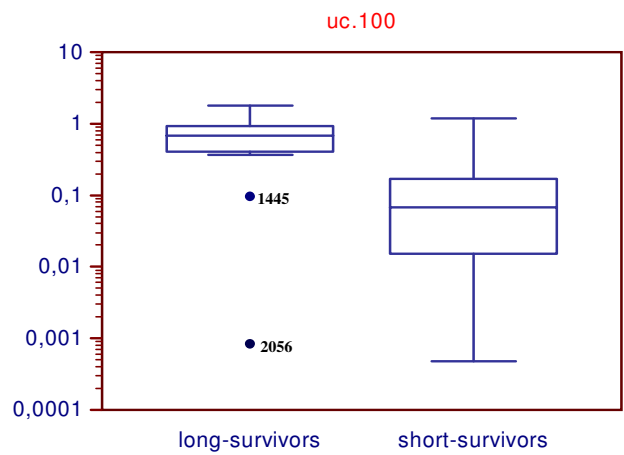

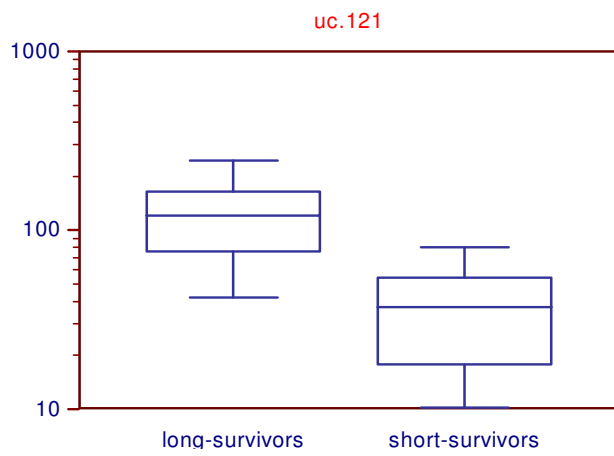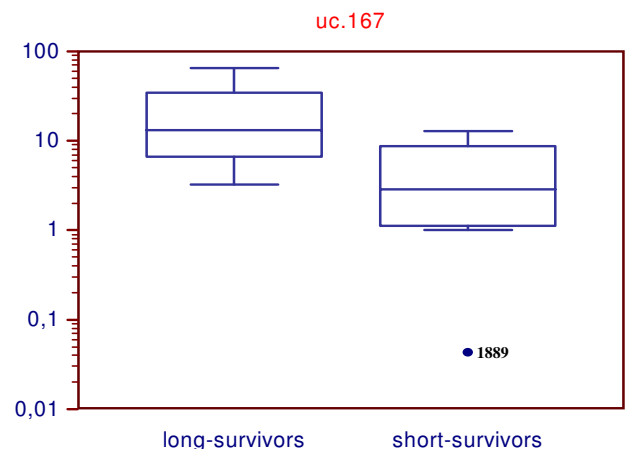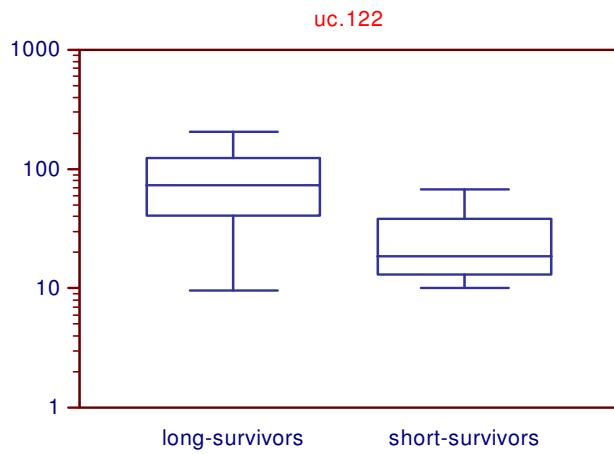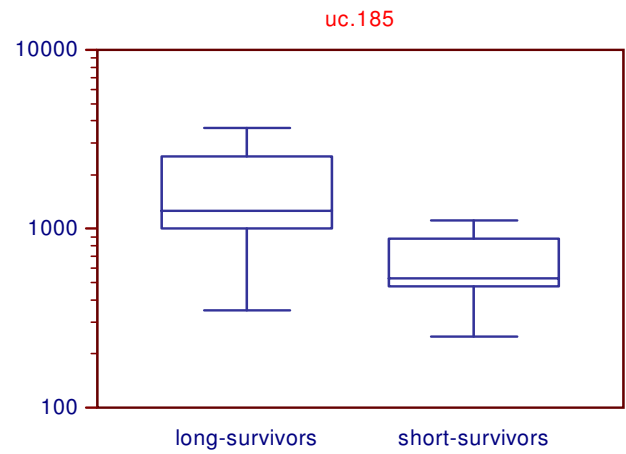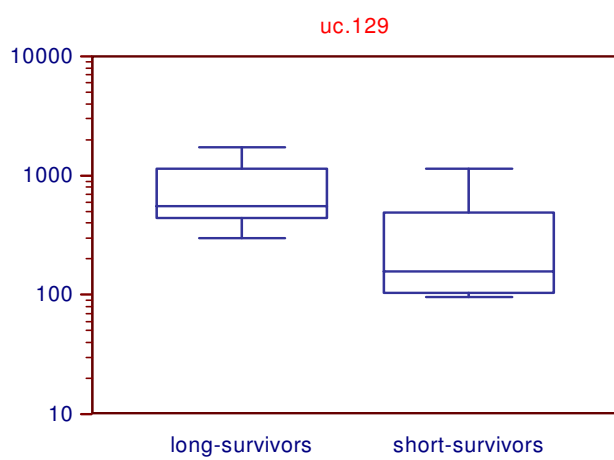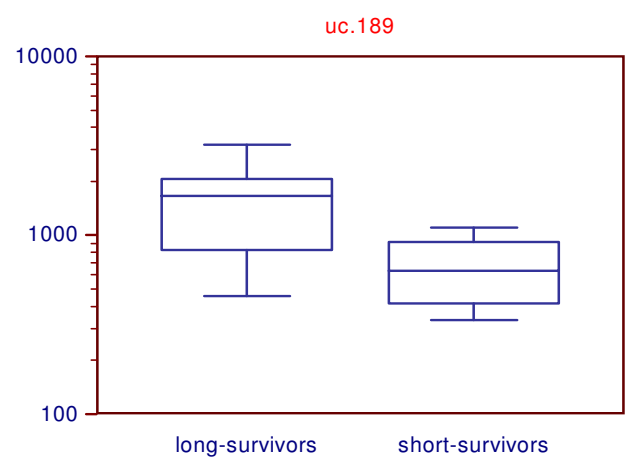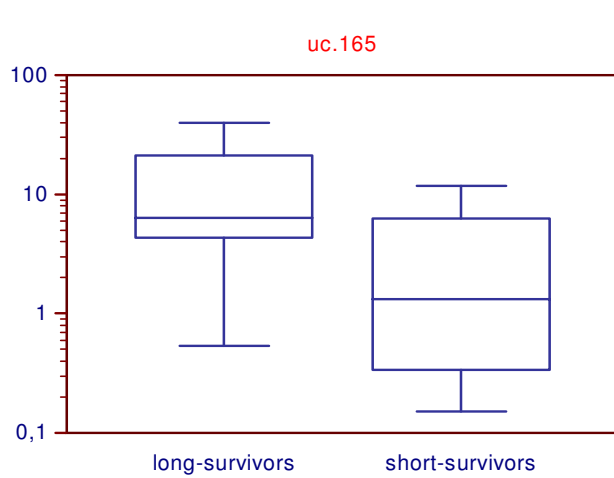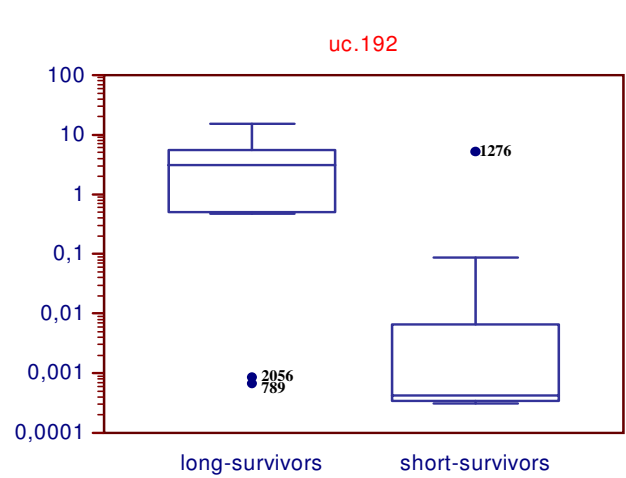

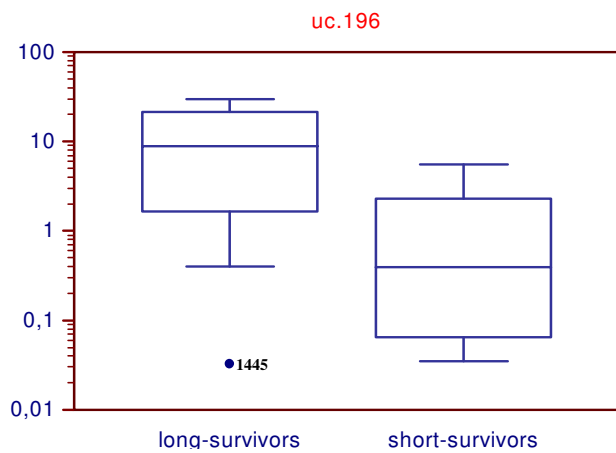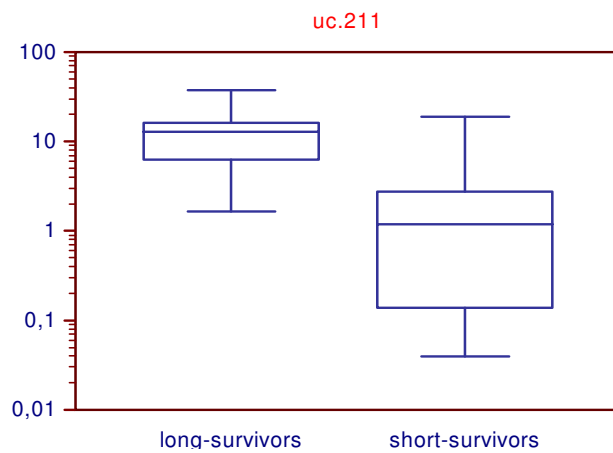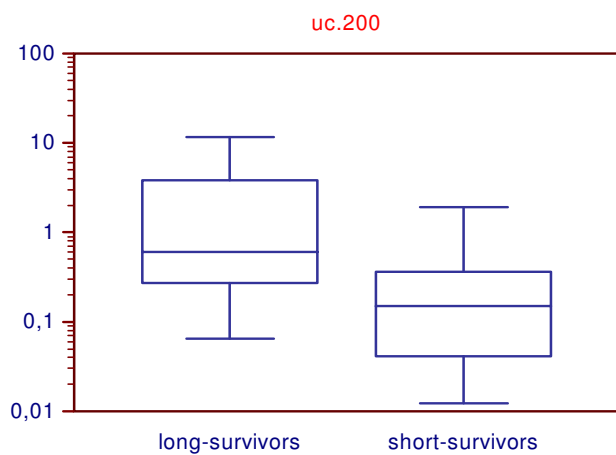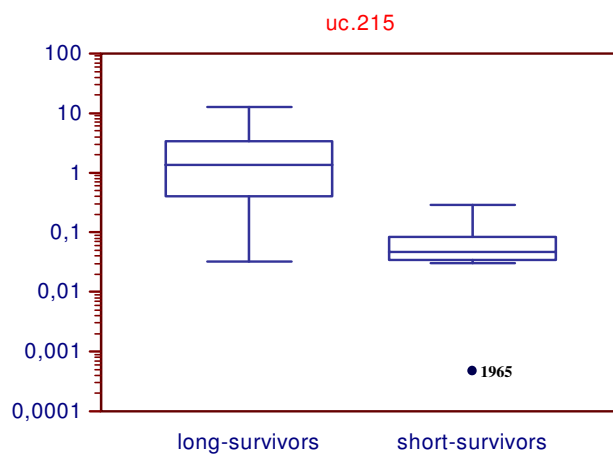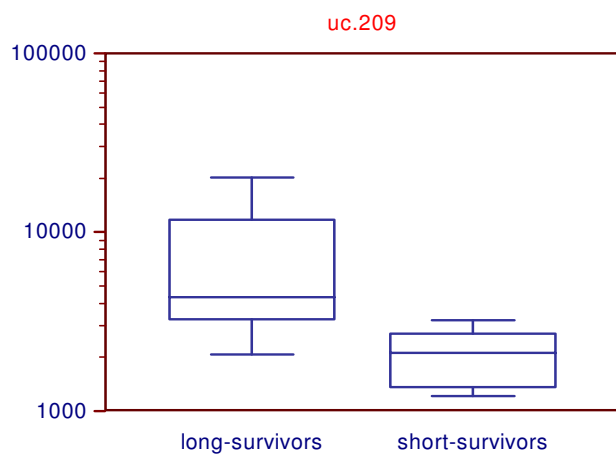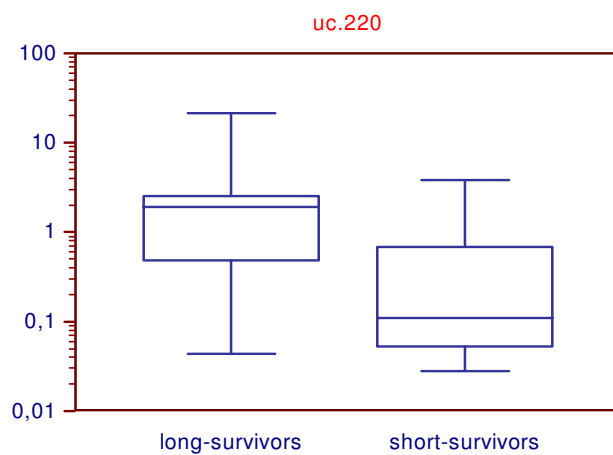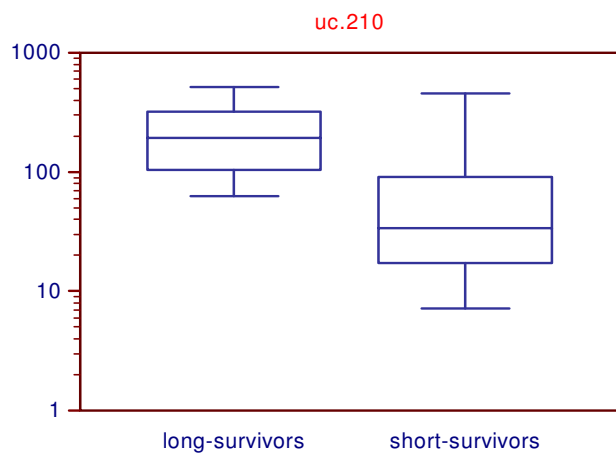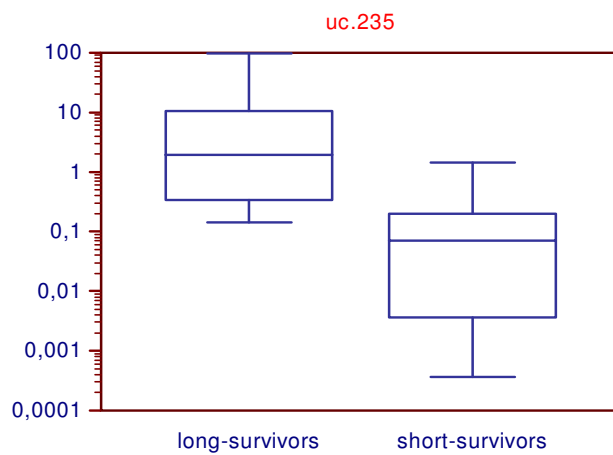

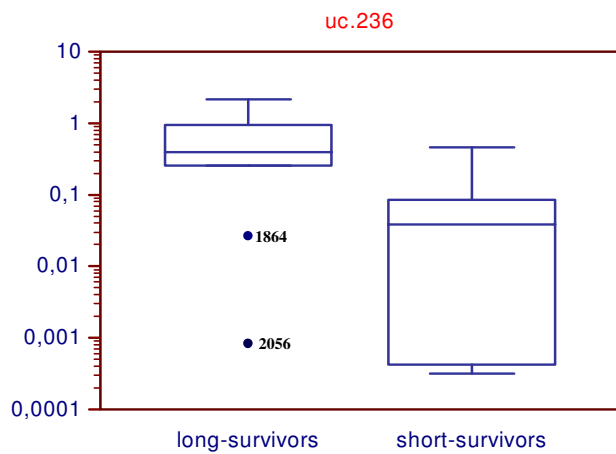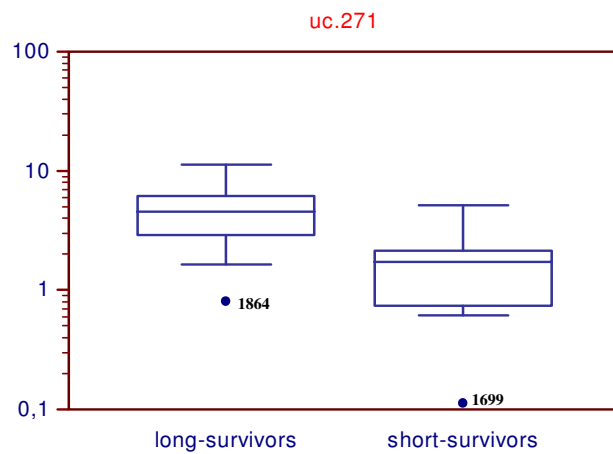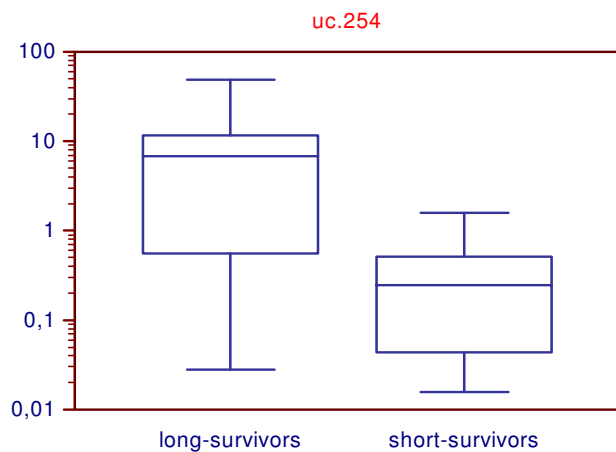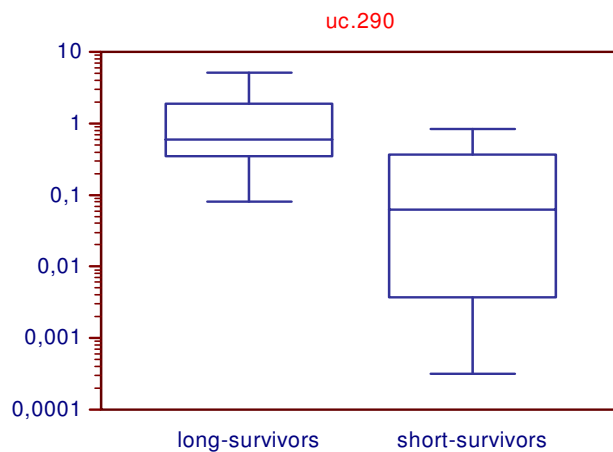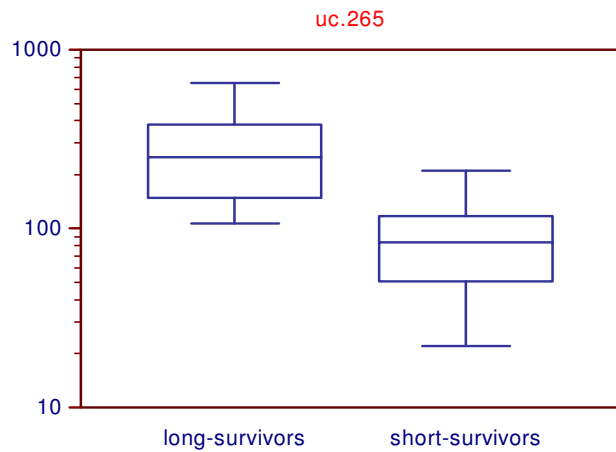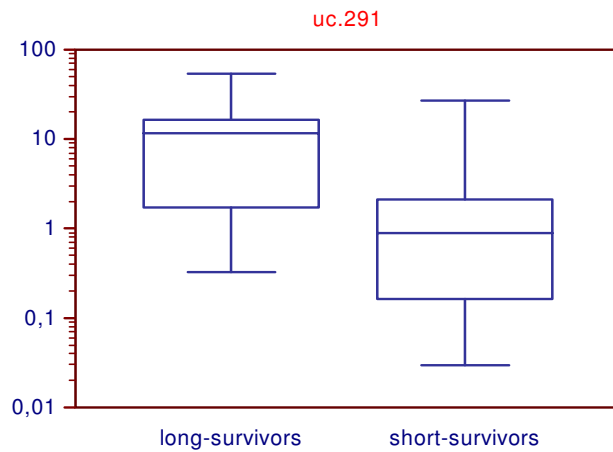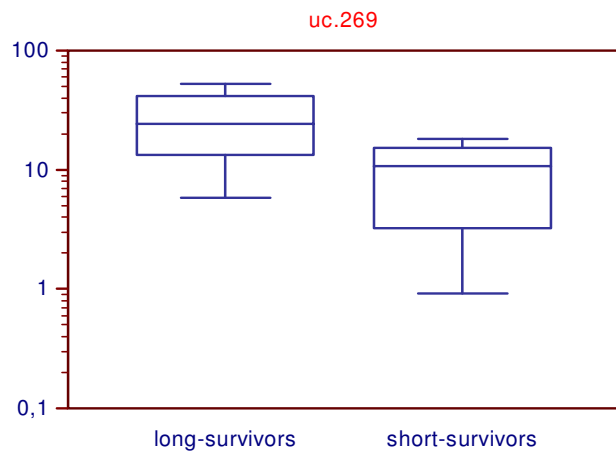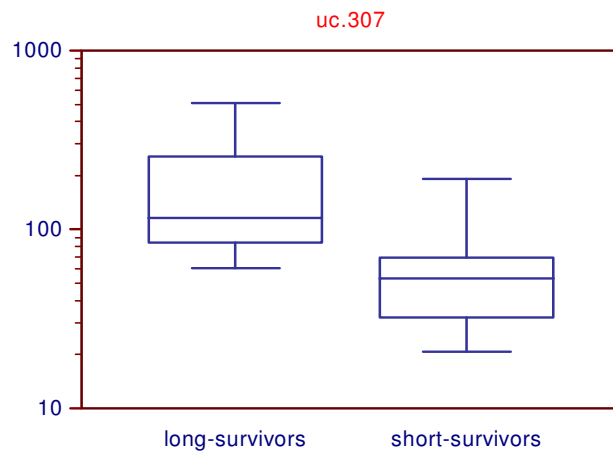

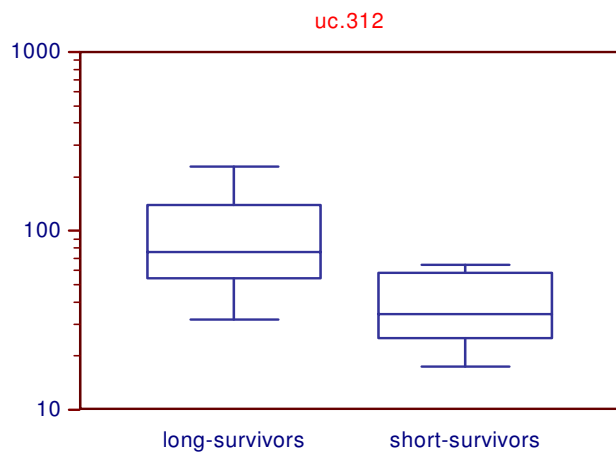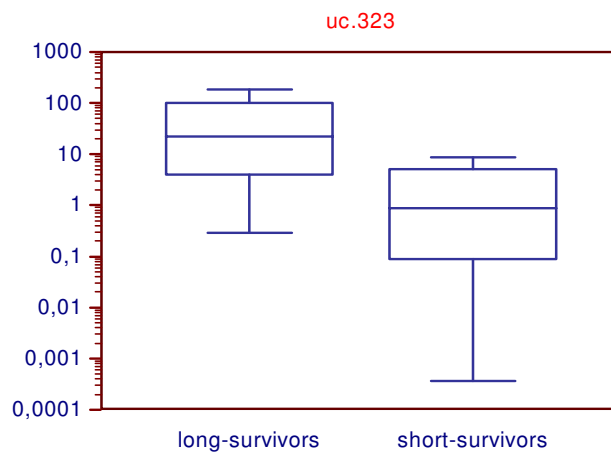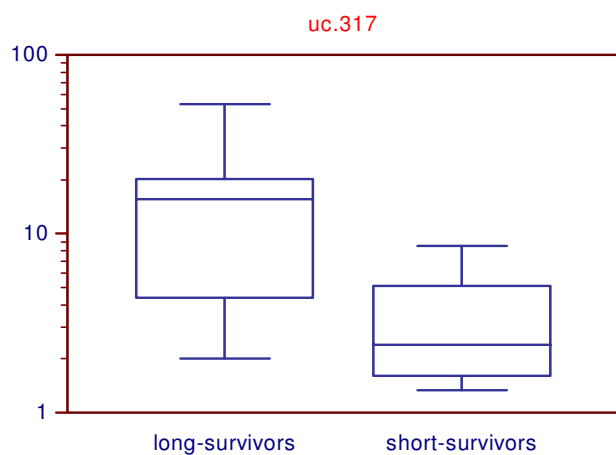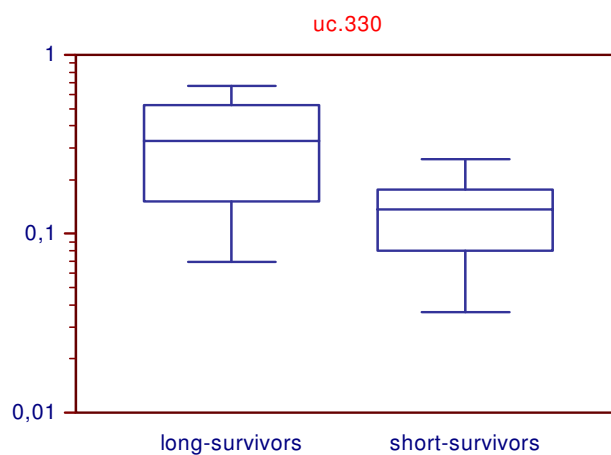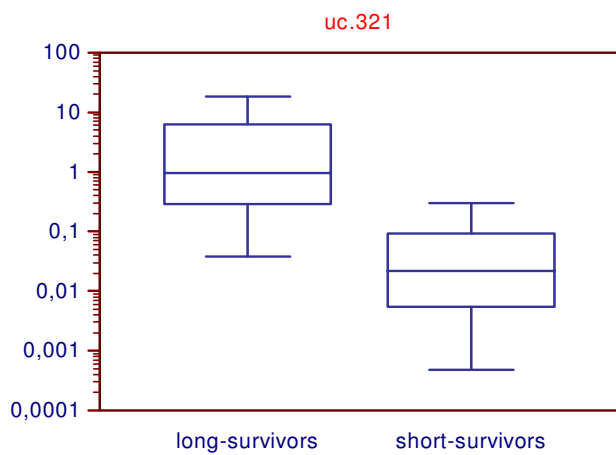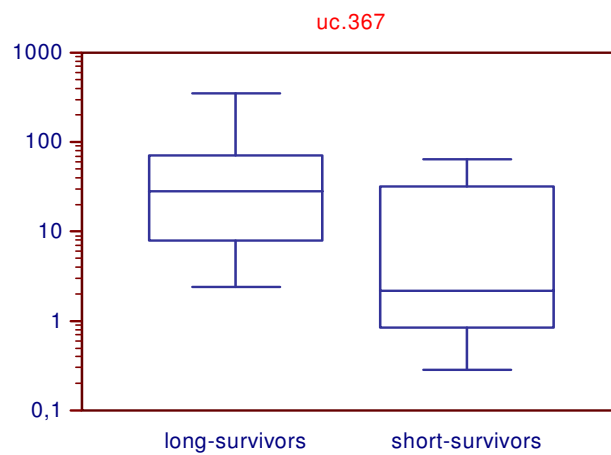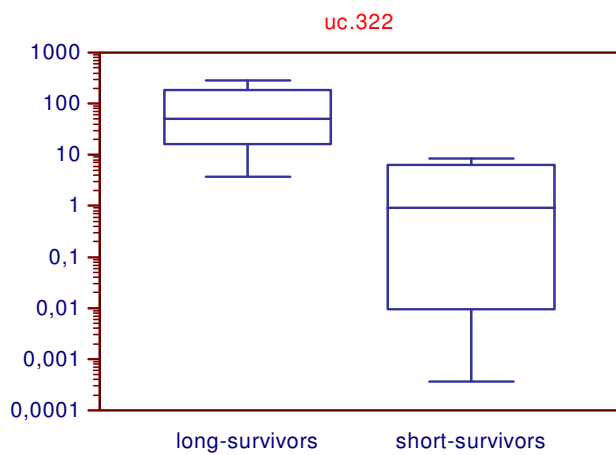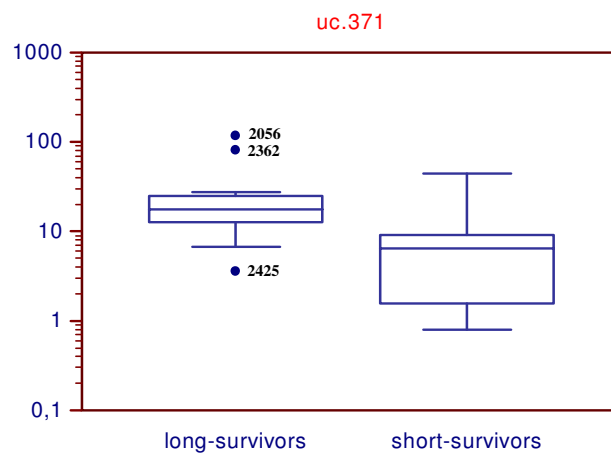

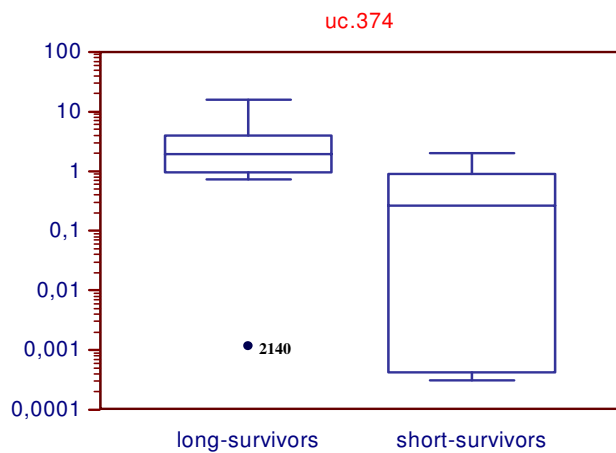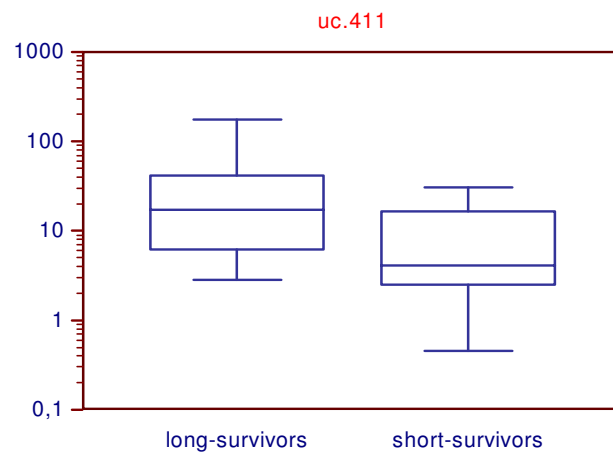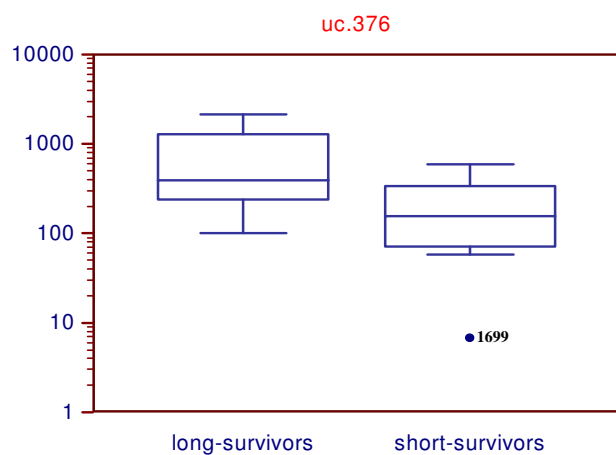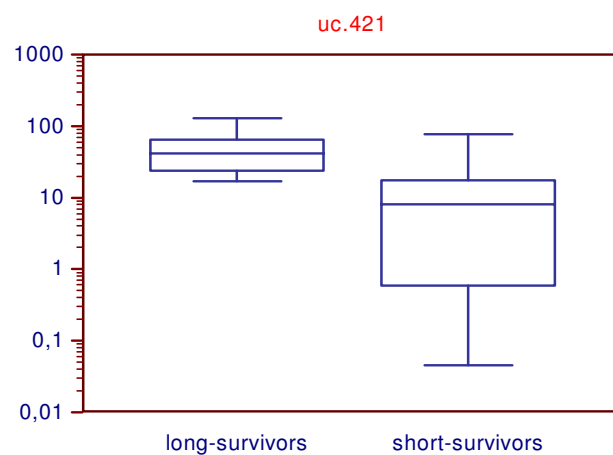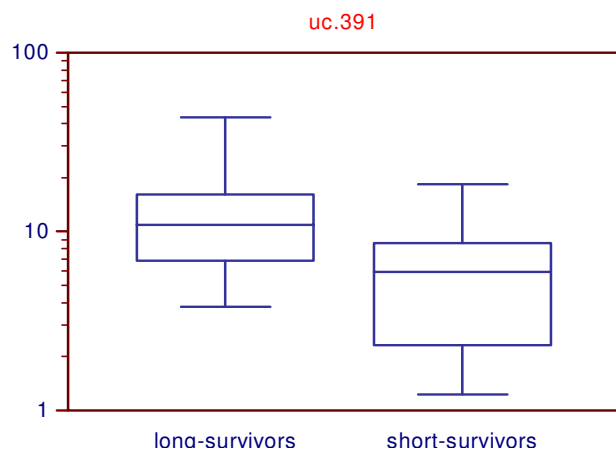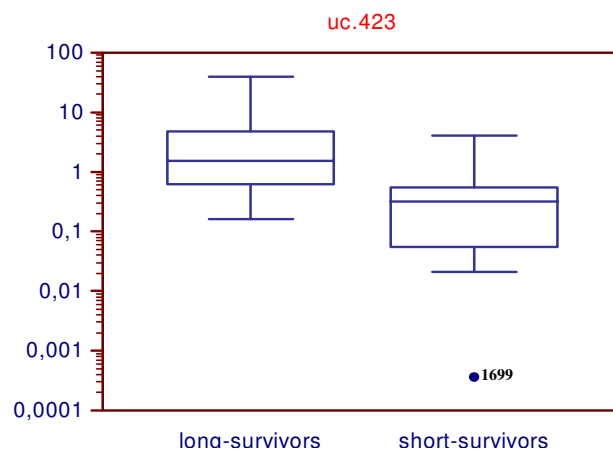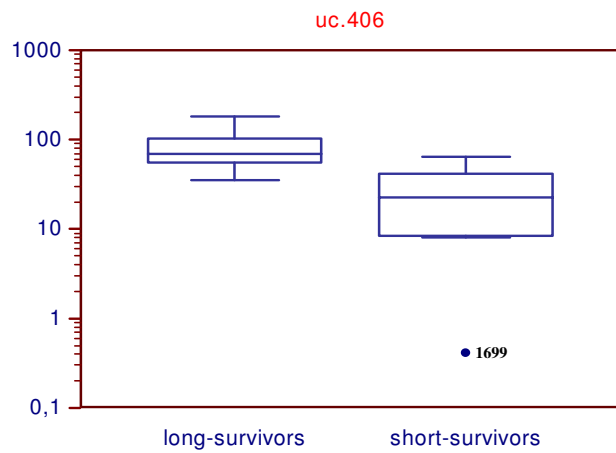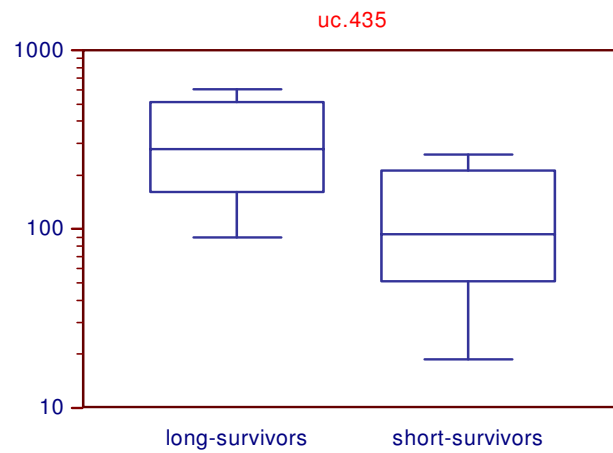

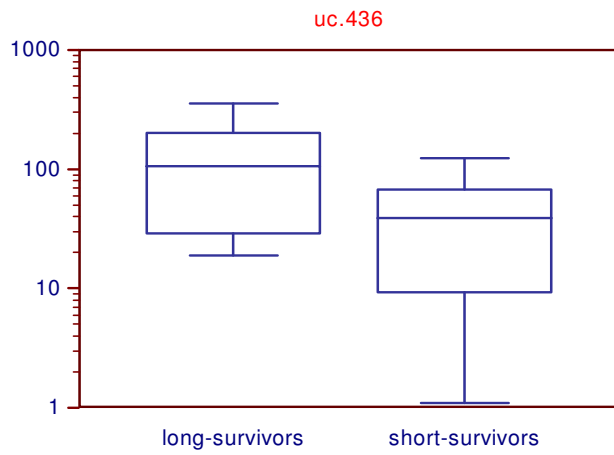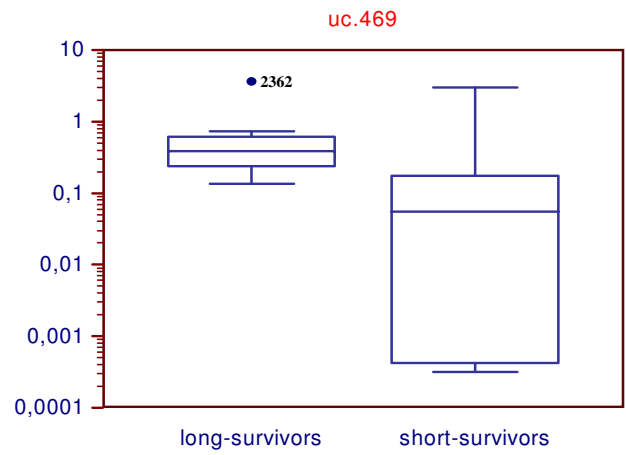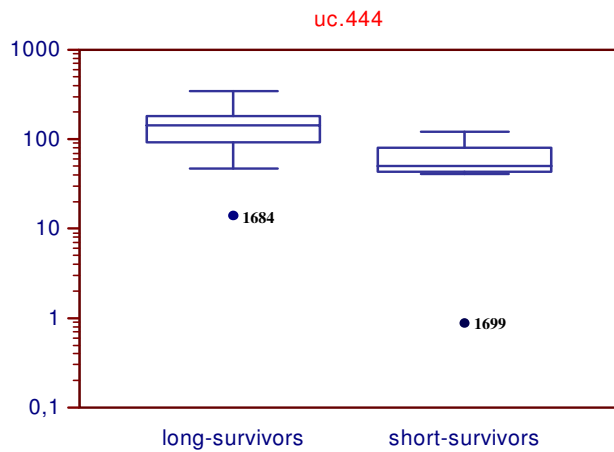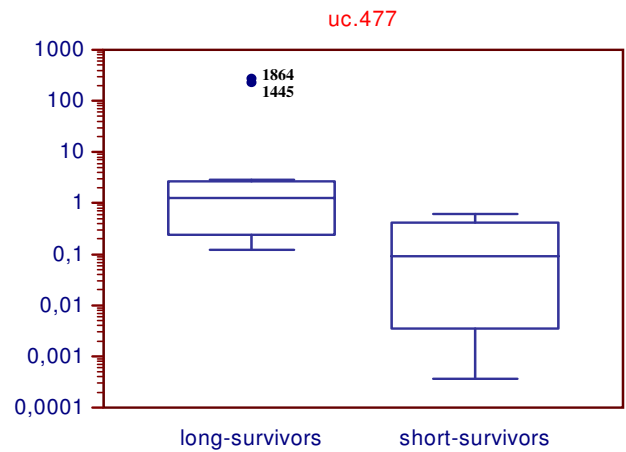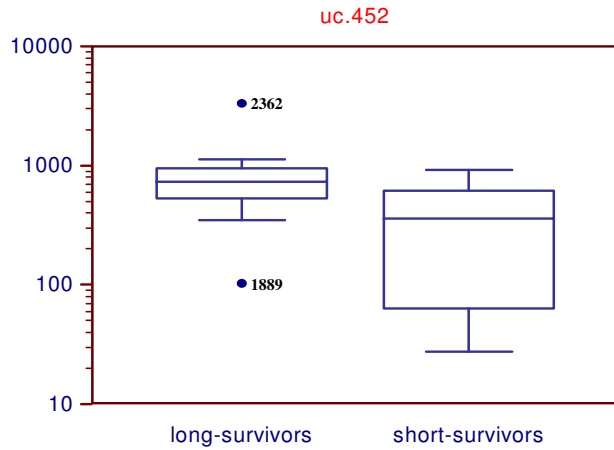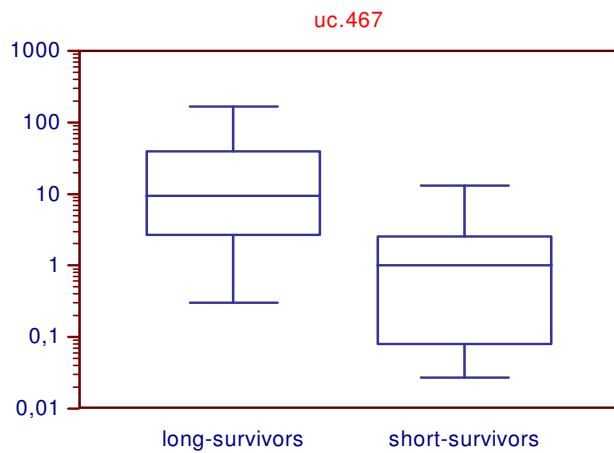

Supplement: Additional file 5 — Figure S1. Box-and-Whisker plots of the comparison among values of T-UCR expression by qPCR (normalized to 18S rRNA, and after logarithmic transformation of original measures) between long- and short-survivors. Each box represents the values from the 25th to 75th percentile, the middle line represents the median, and a line extends from the minimum to the maximum value, excluding outliers which are displayed as blue dots. Nearby each outlier value is indicated the ID of the corresponding NB sample. Relative expression data were multiplied by 104. [file 1471-2407-9-441-S5.PDF]

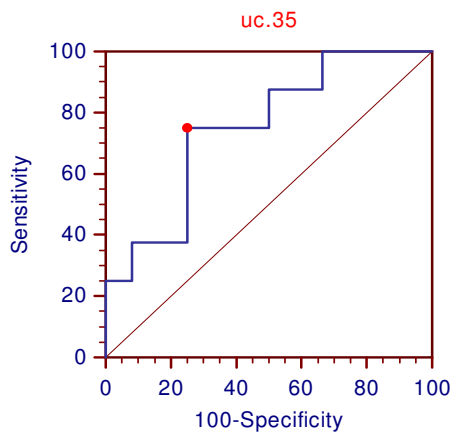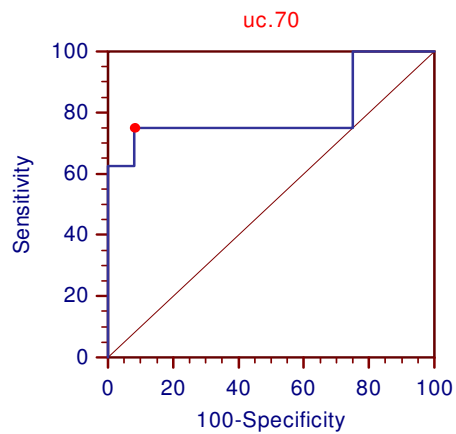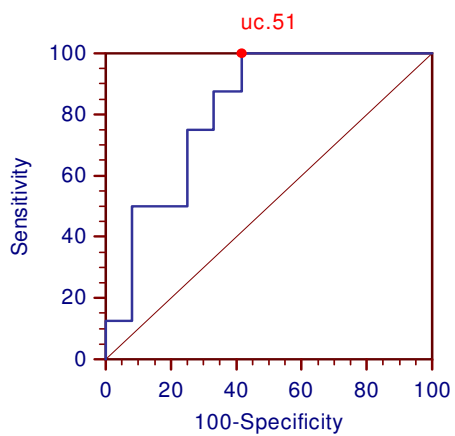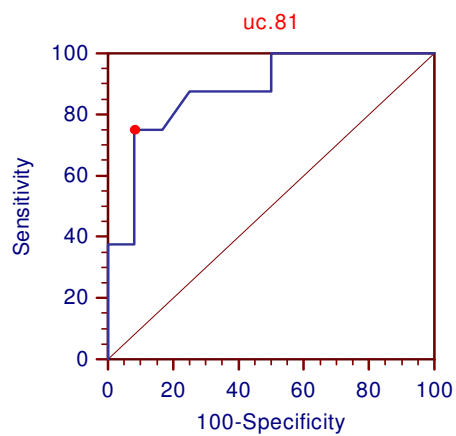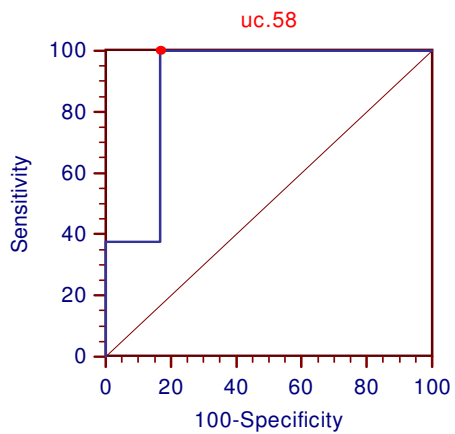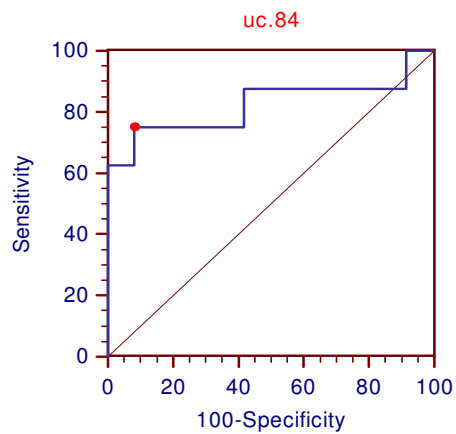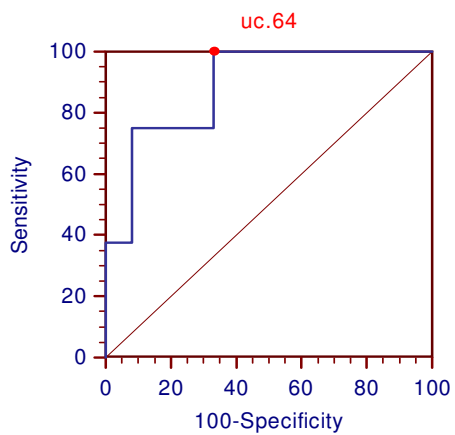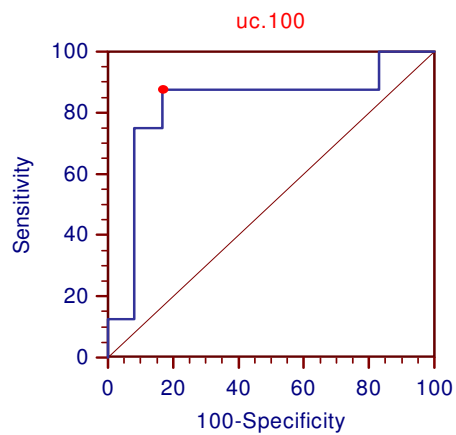

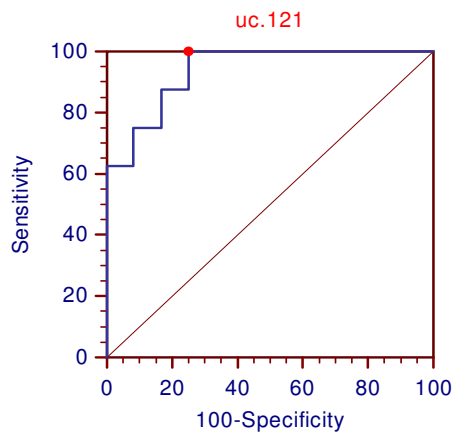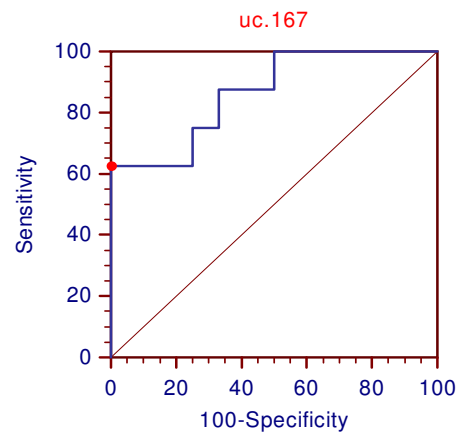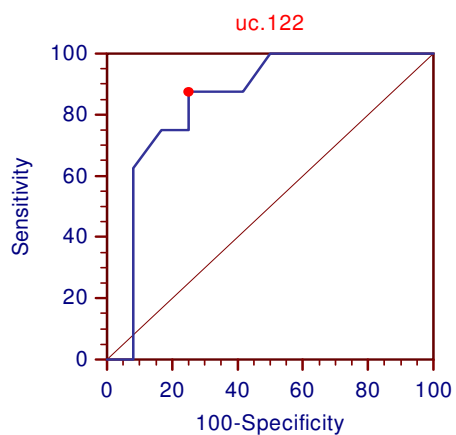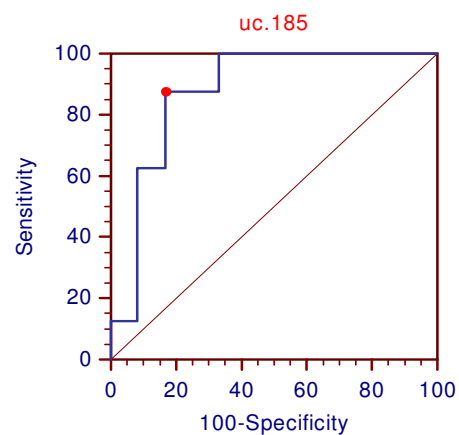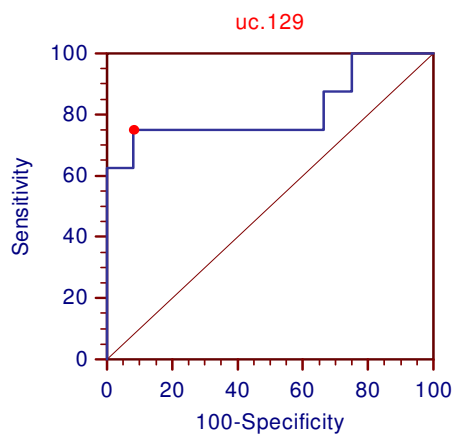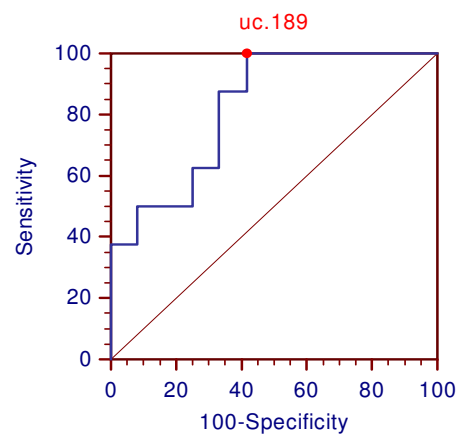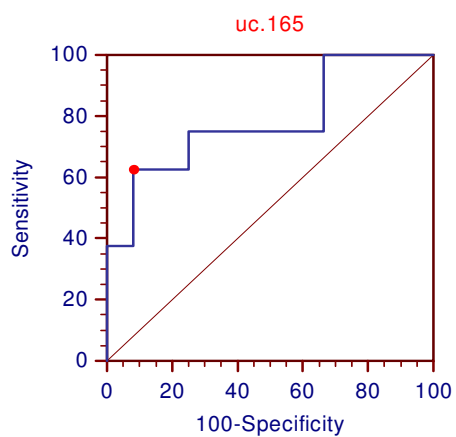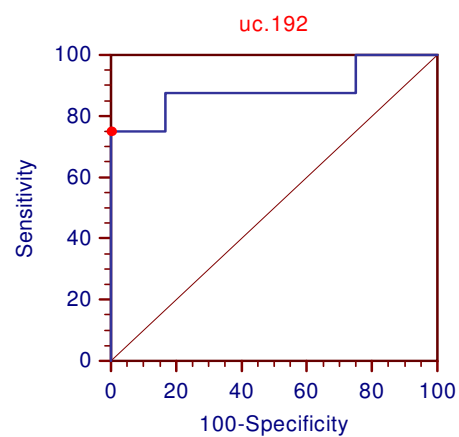

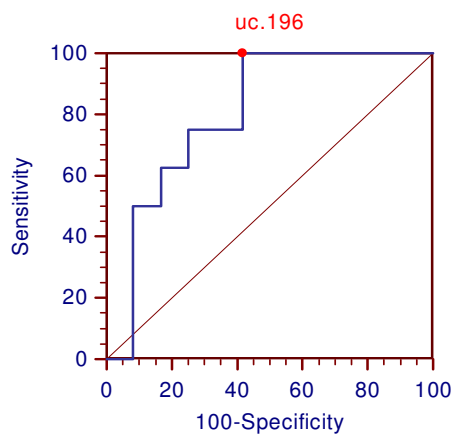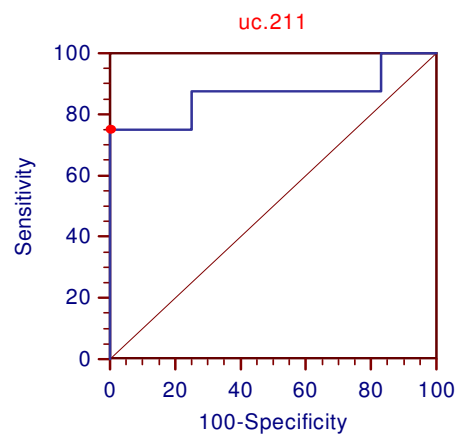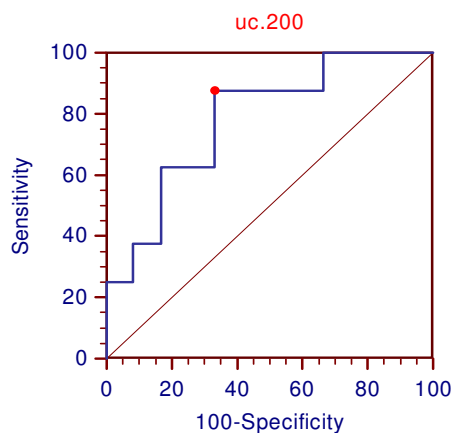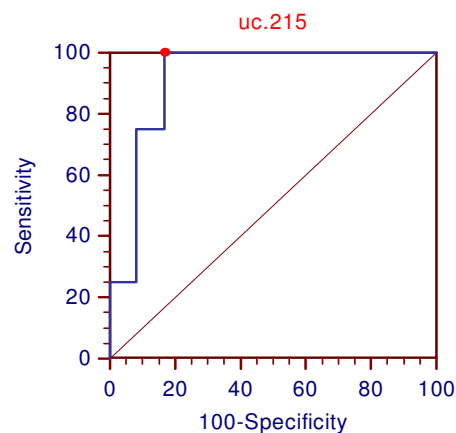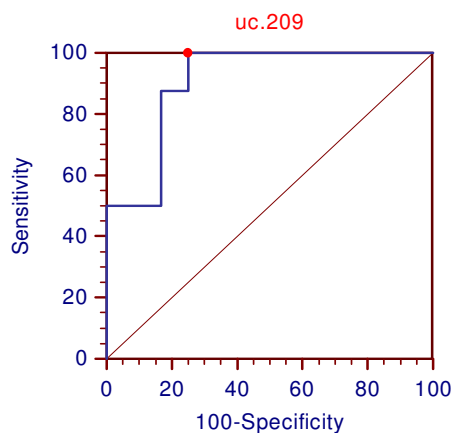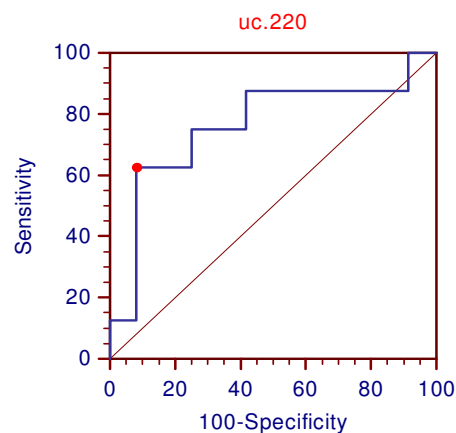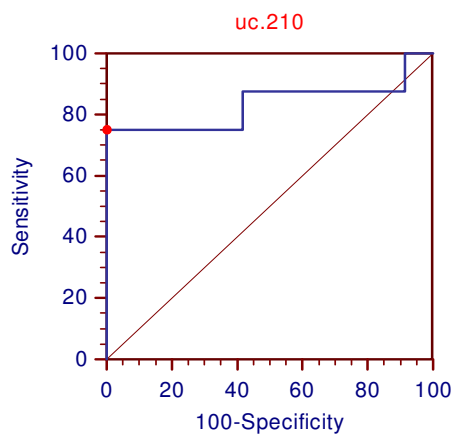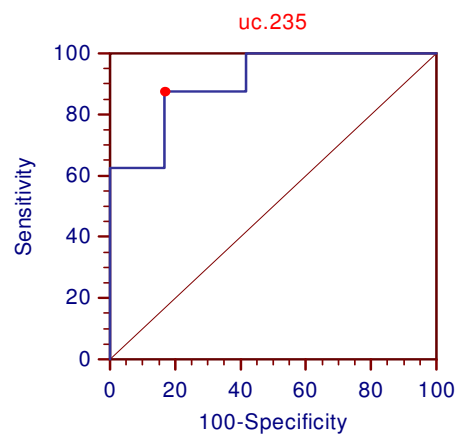

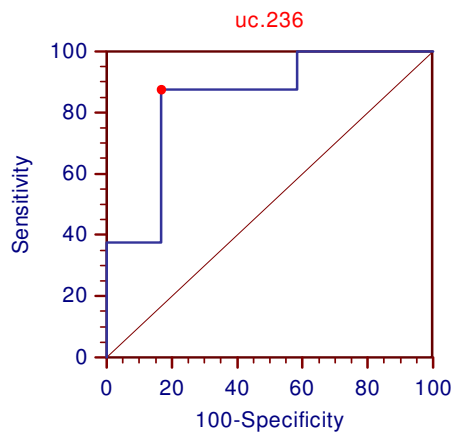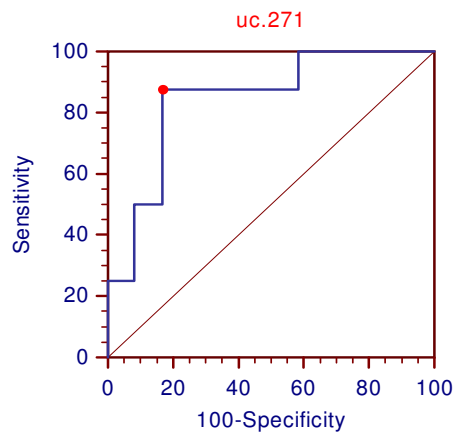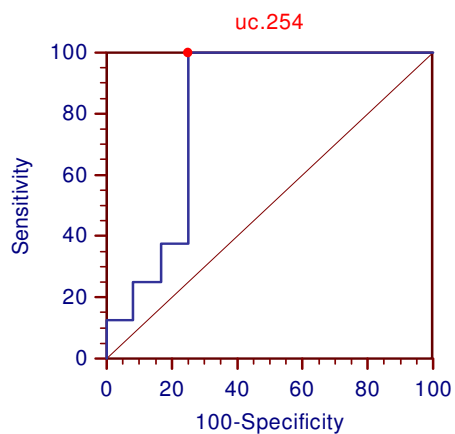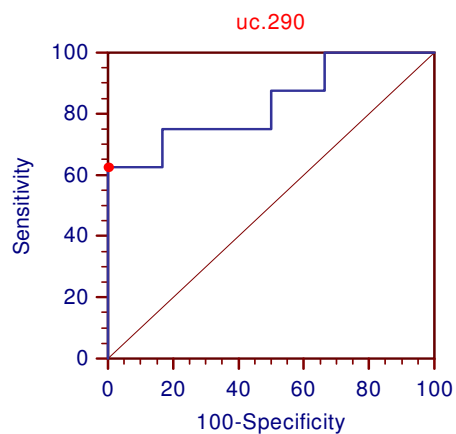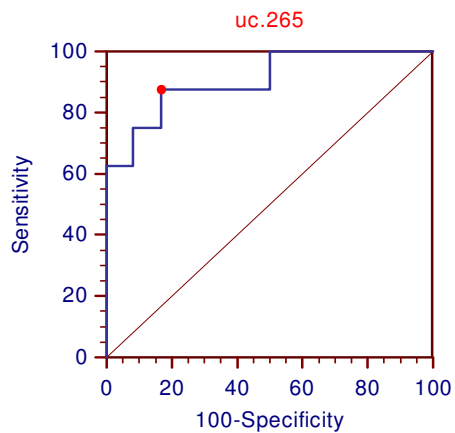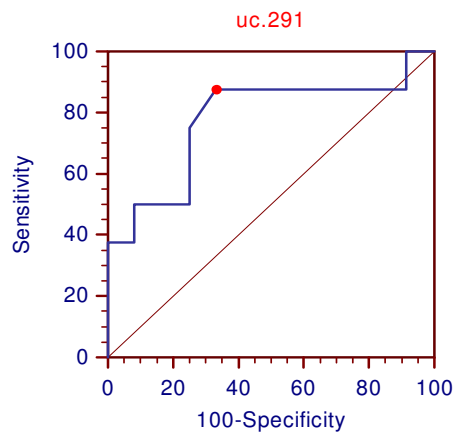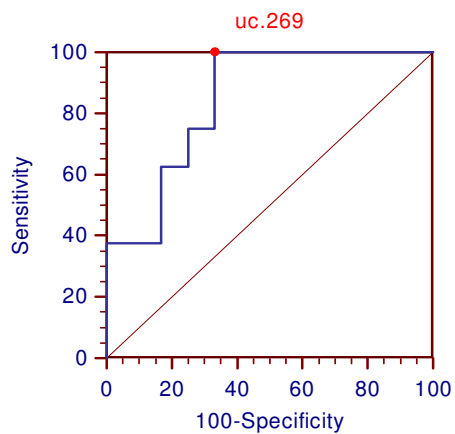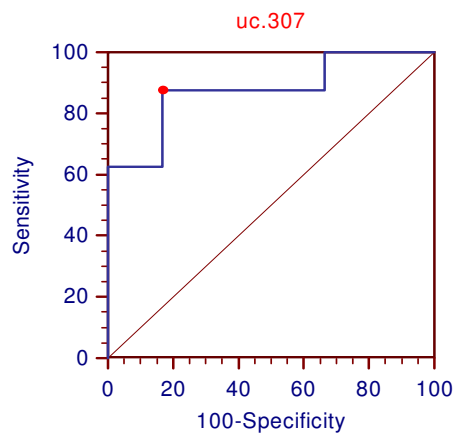

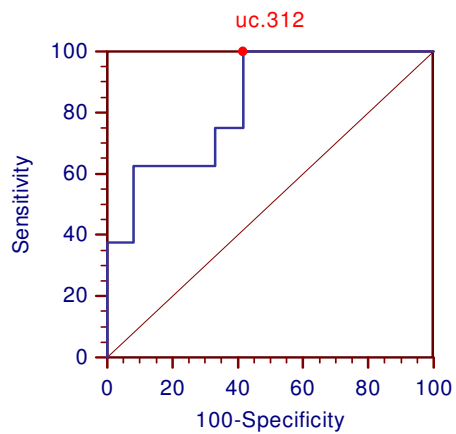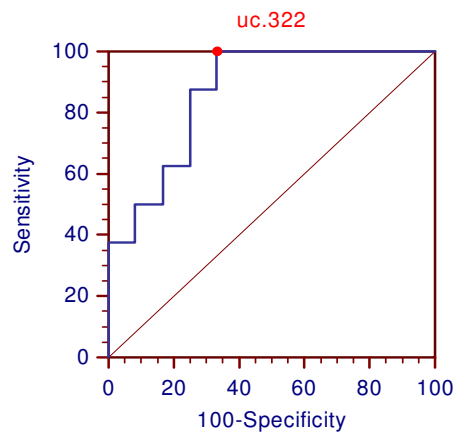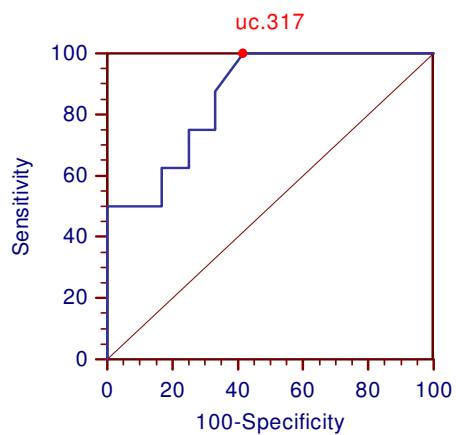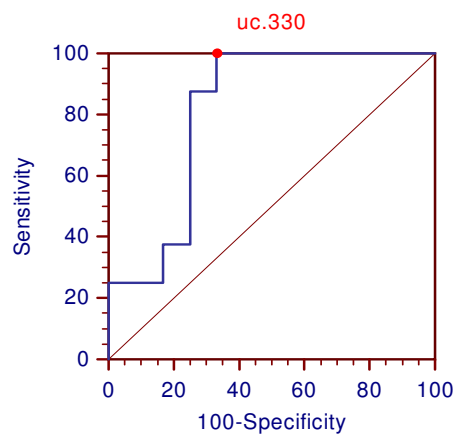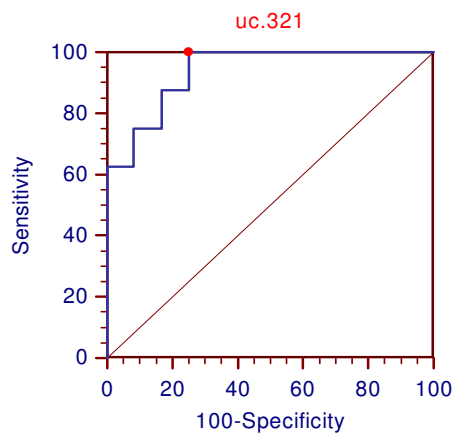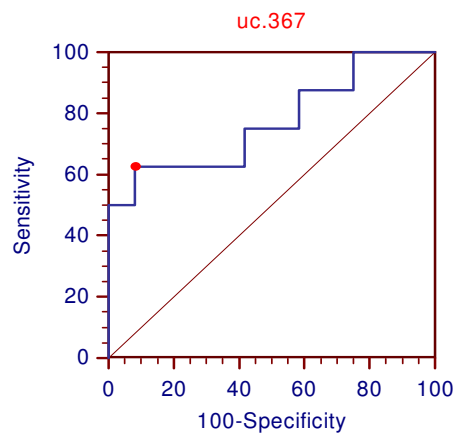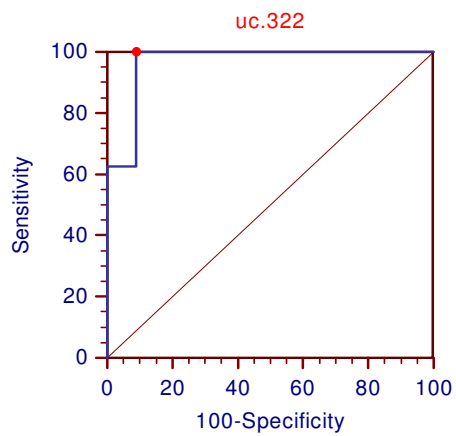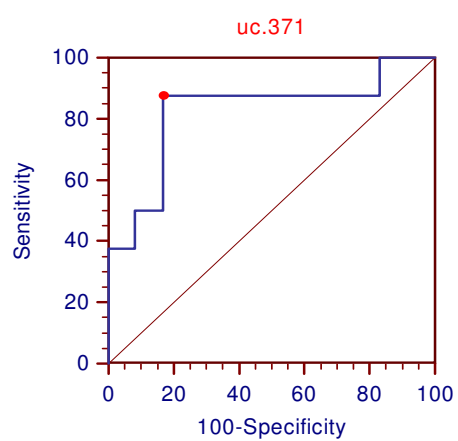

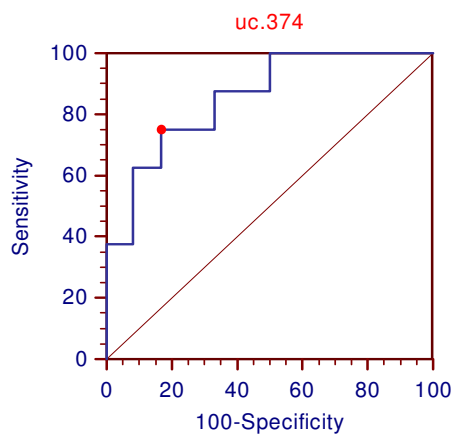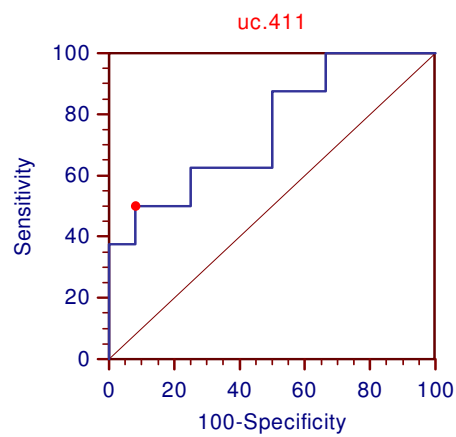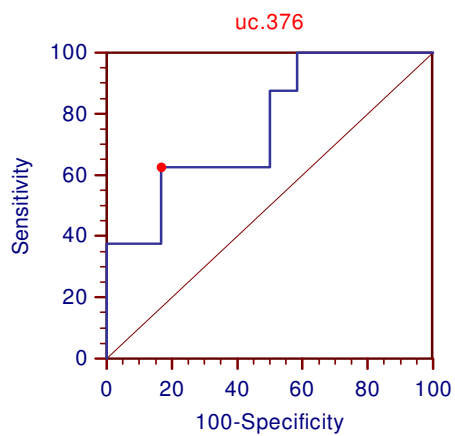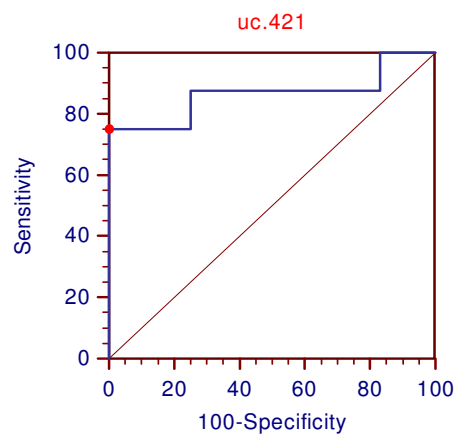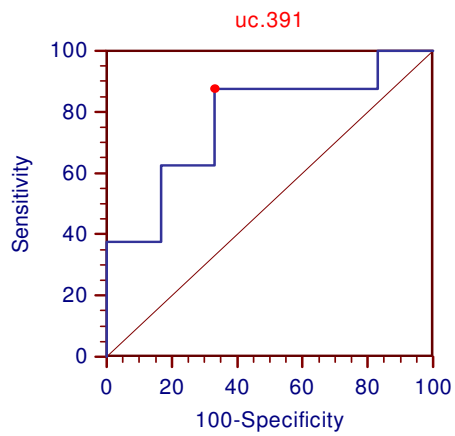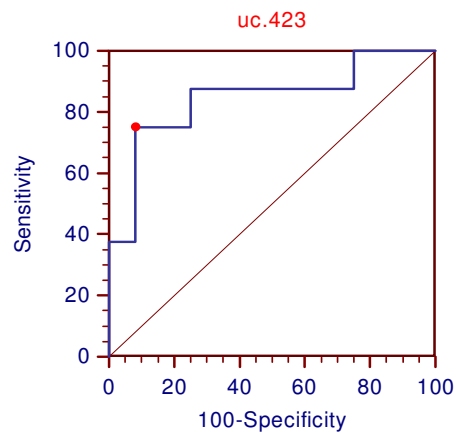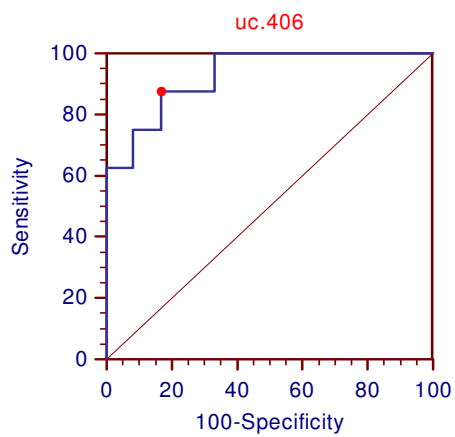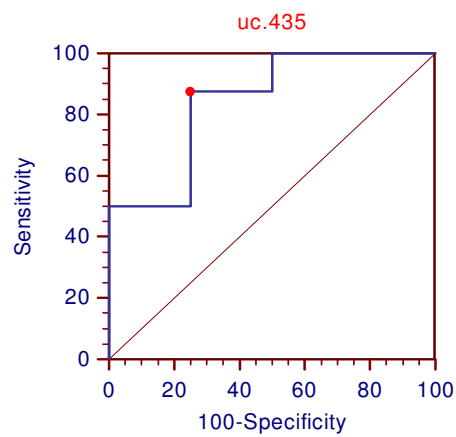

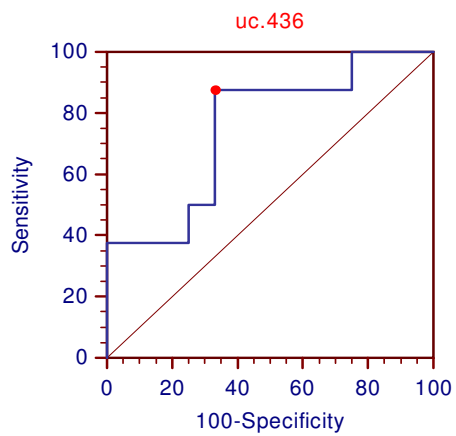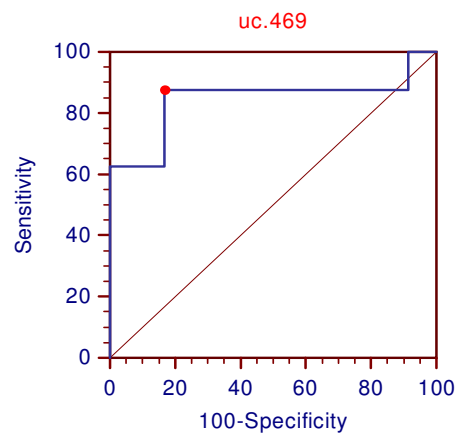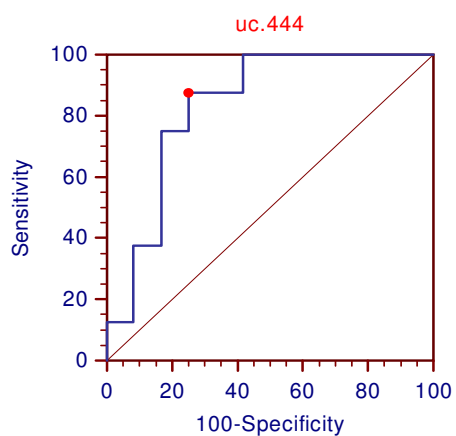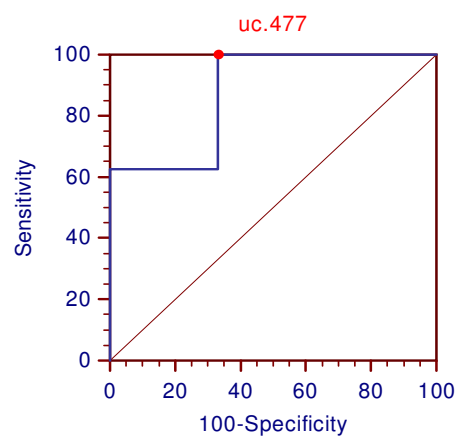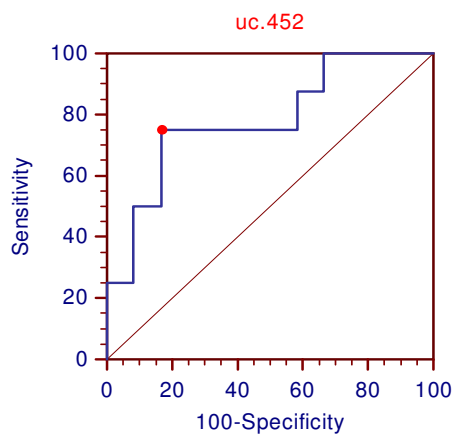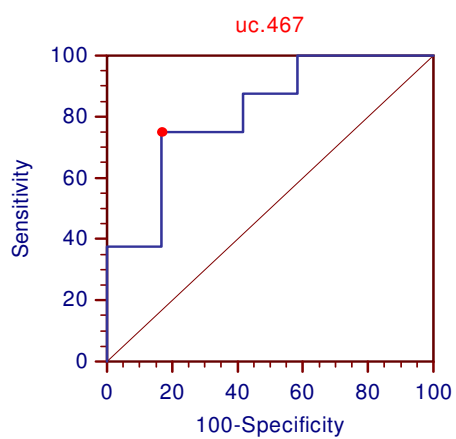

Supplement: Additional file 6 — Figure S2. ROC curves based on qPCR results of the 54 T-UCRs differently expressed between long- and short-survivors. In each graph the point indicated with a red dot is the value corresponding to the highest average of sensitivity and specificity. [file 1471-2407-9-441-S6.PDF]

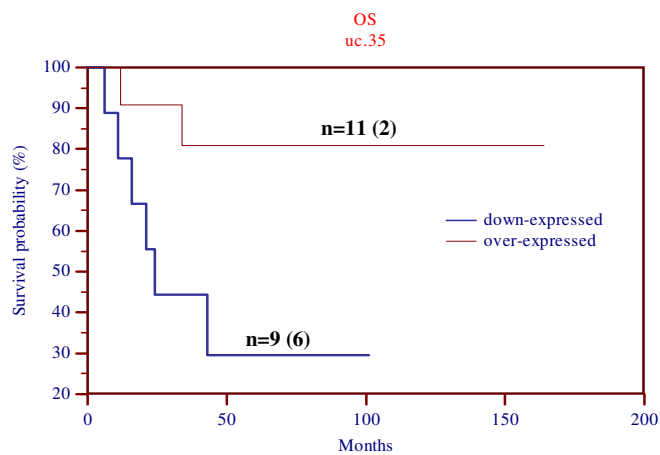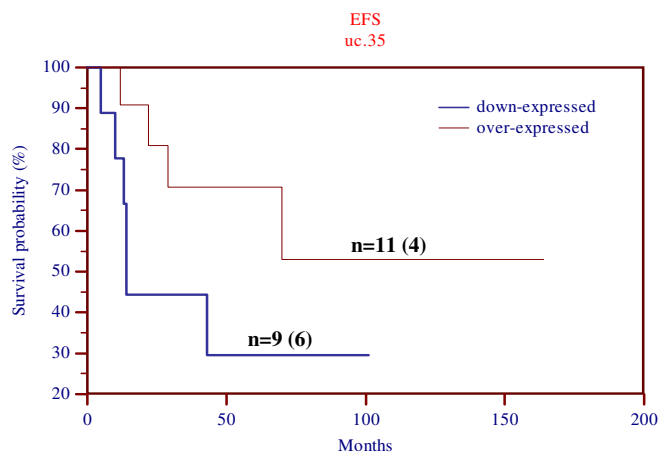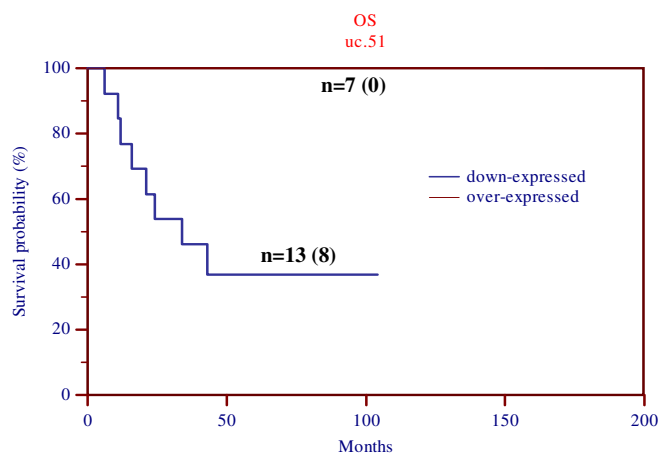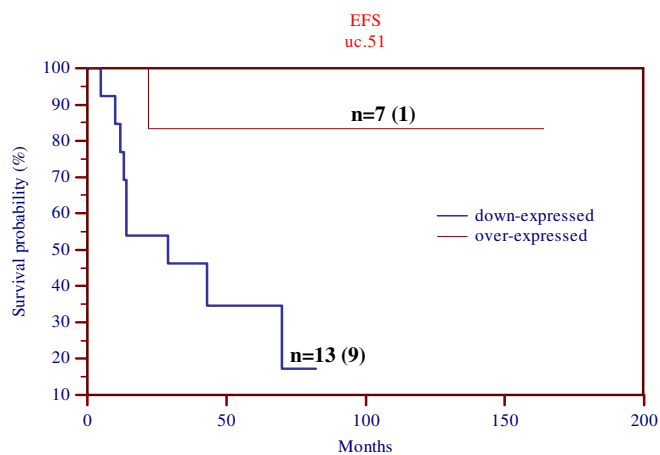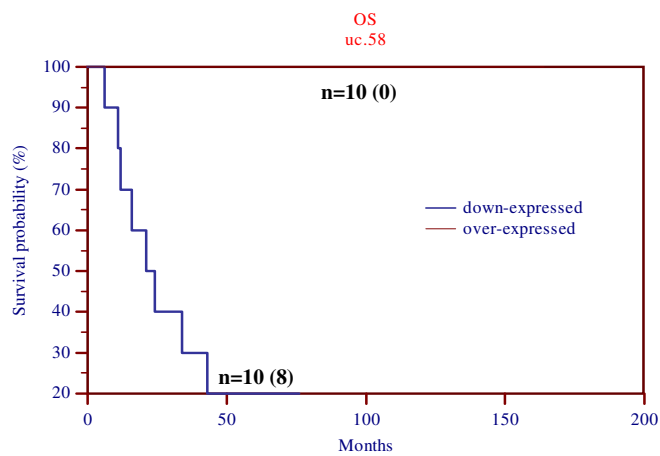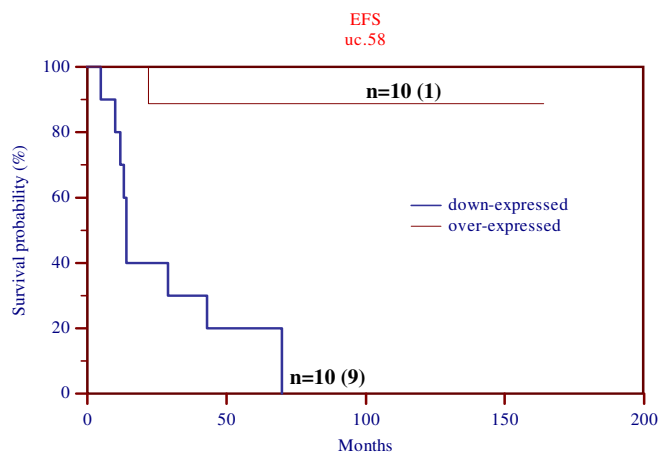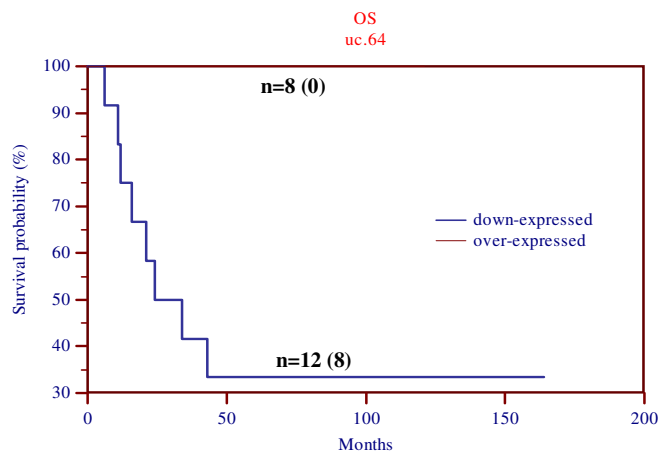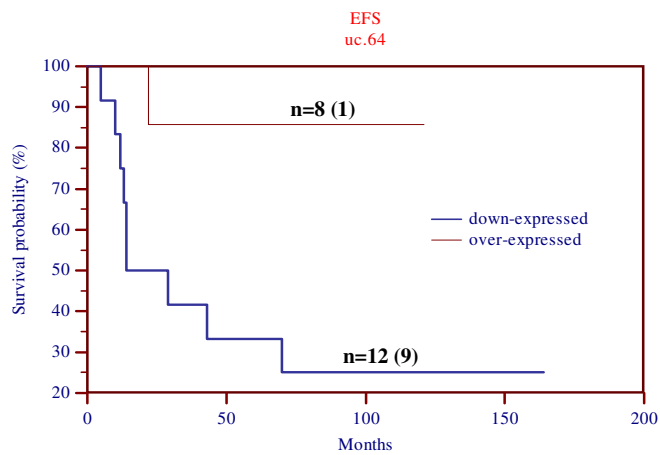

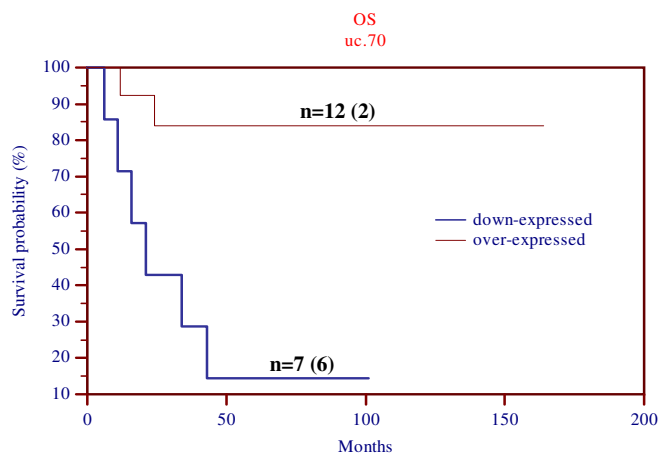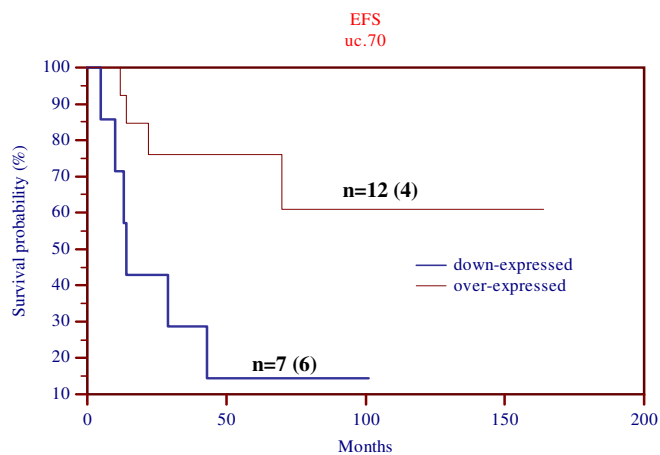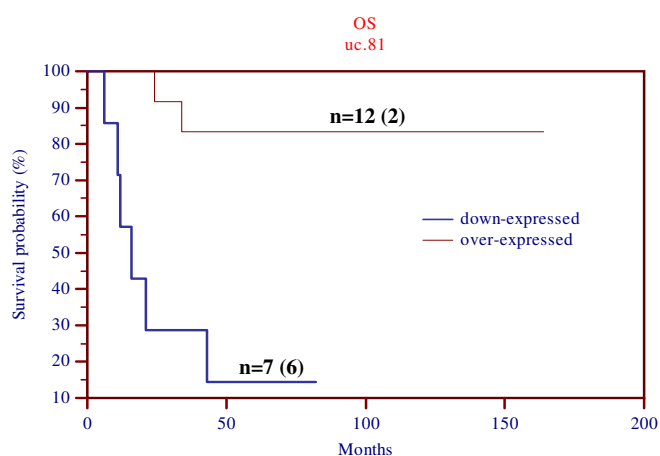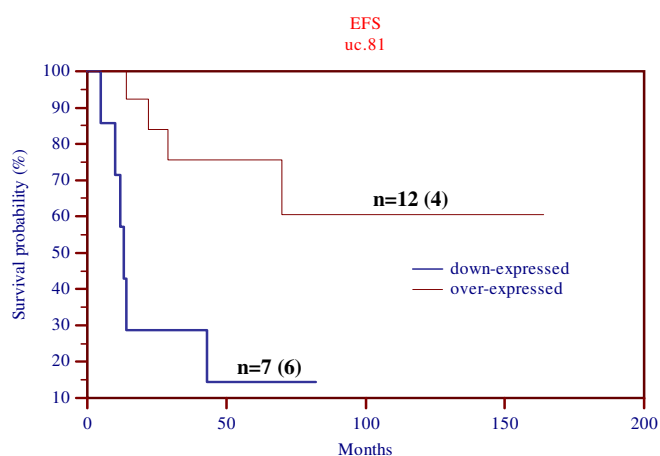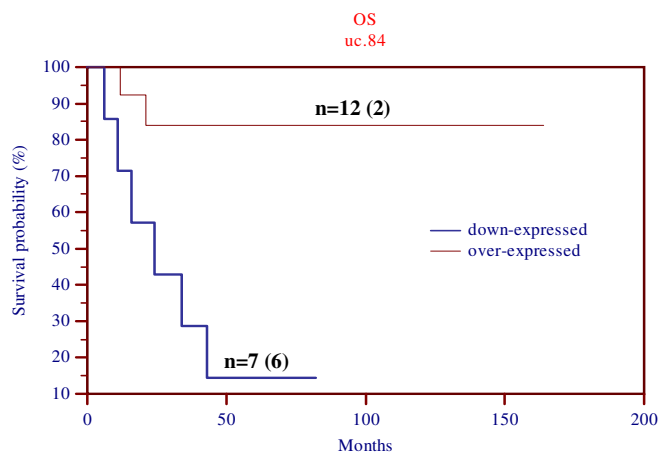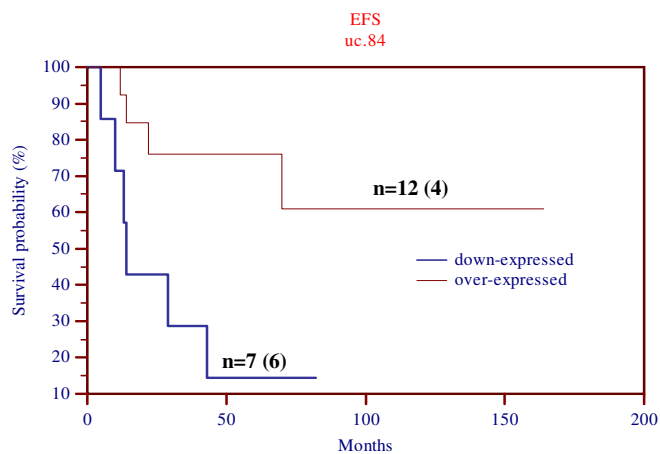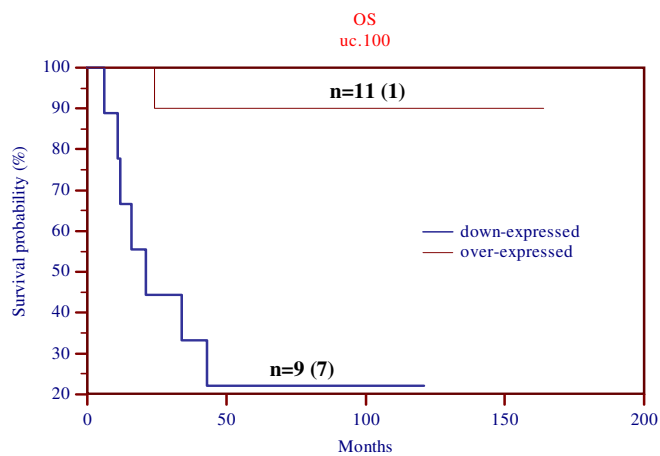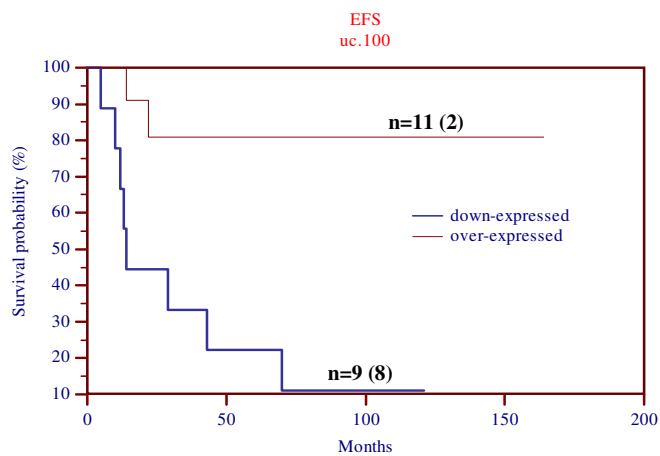

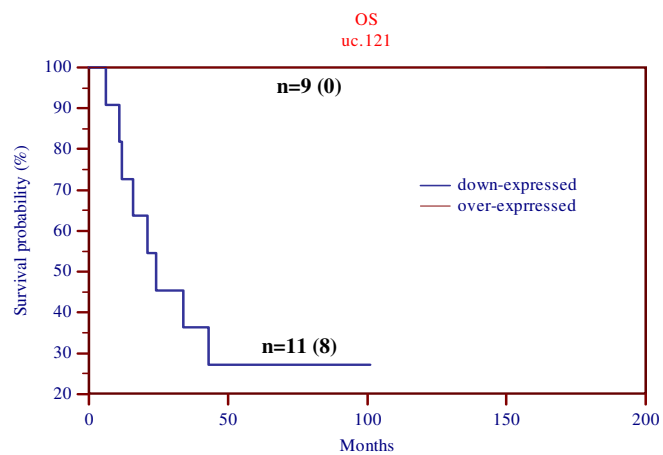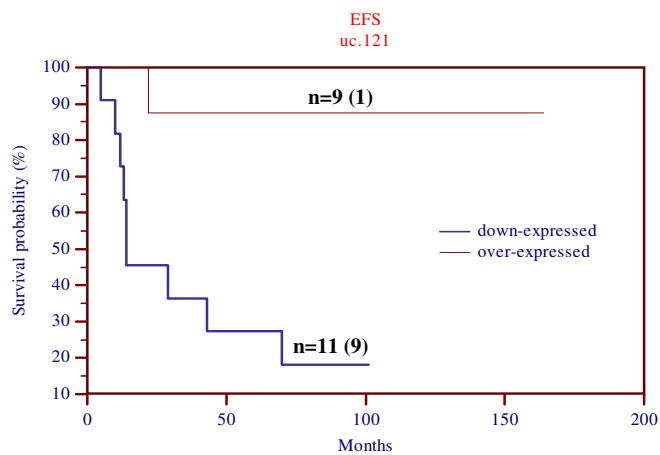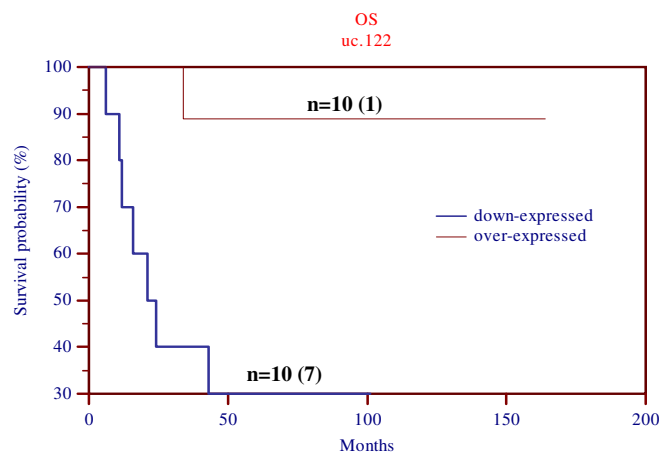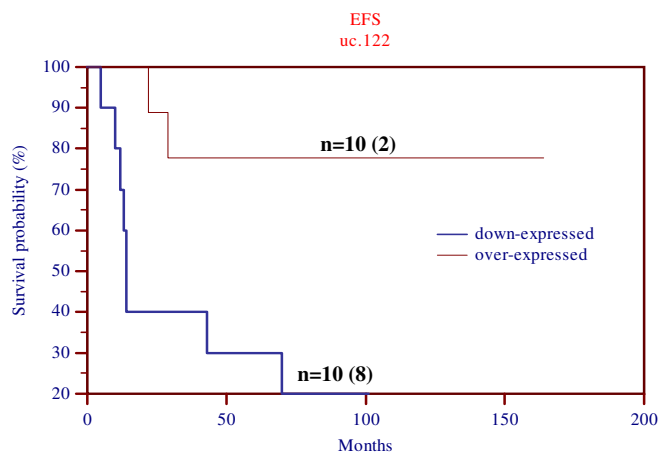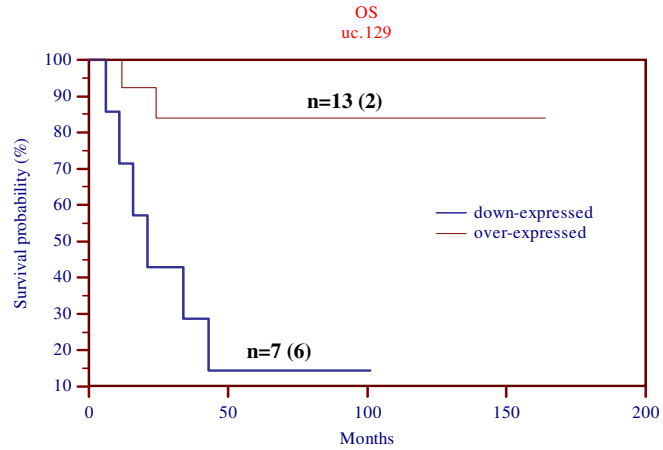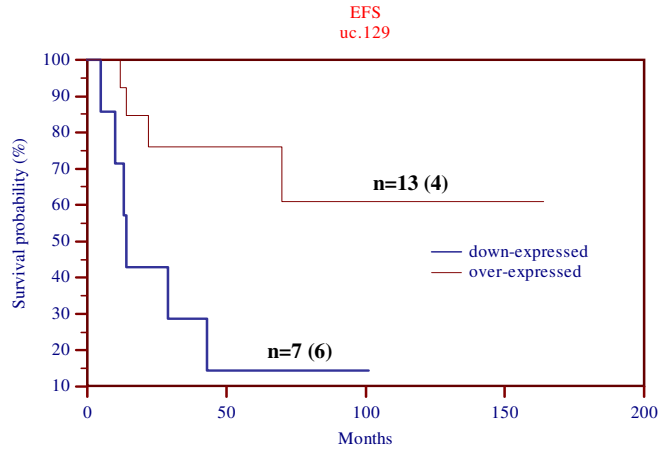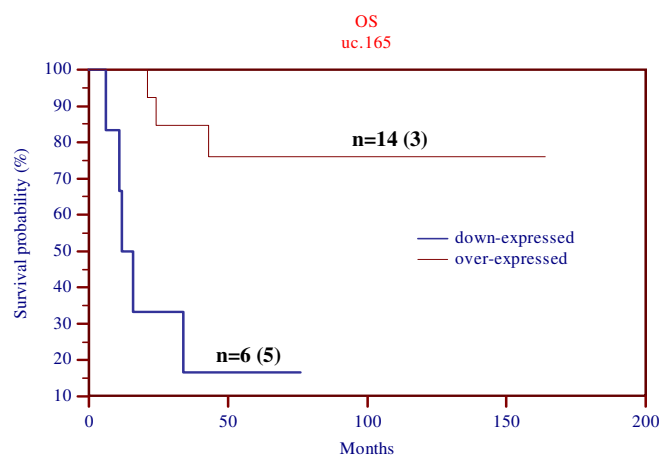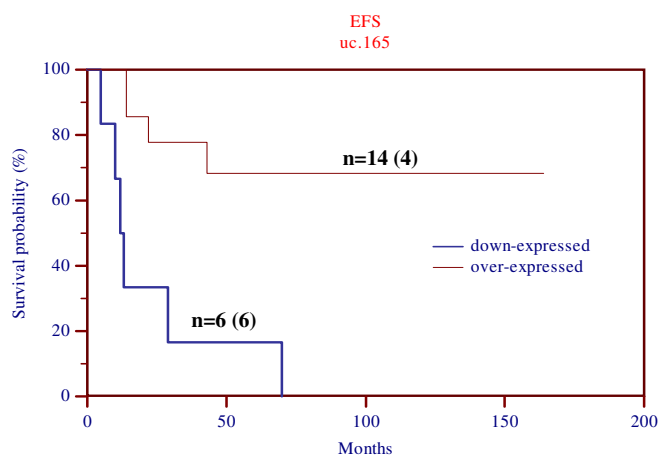

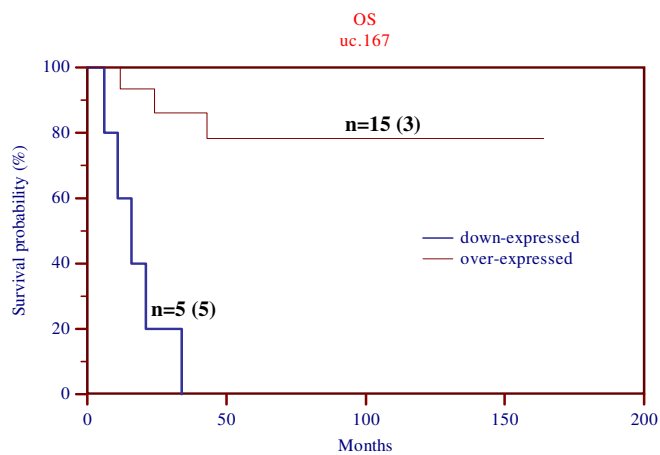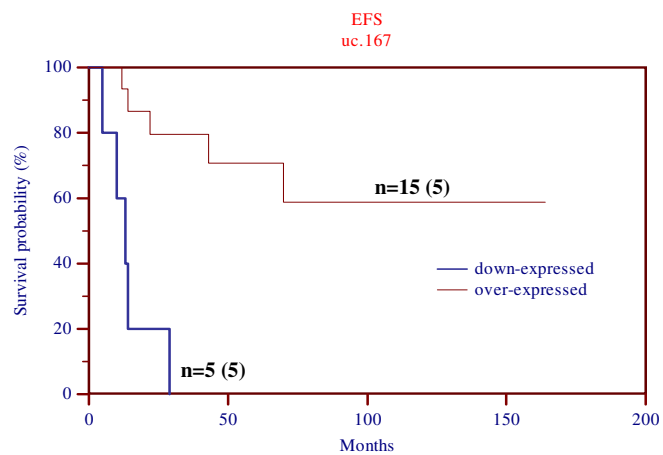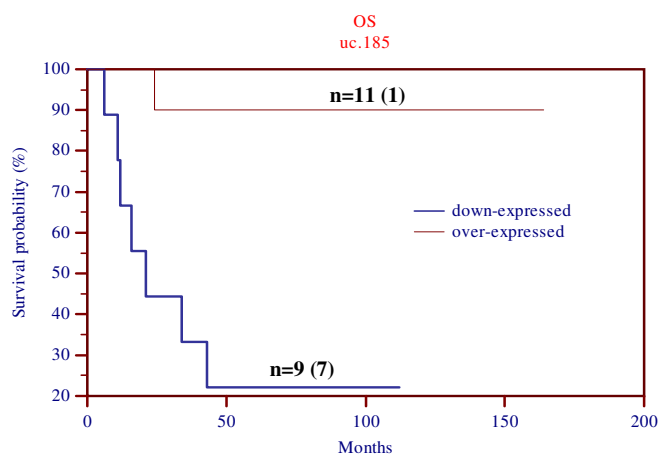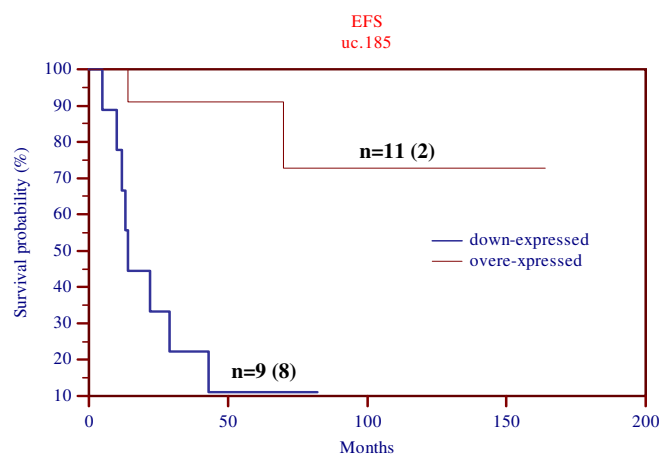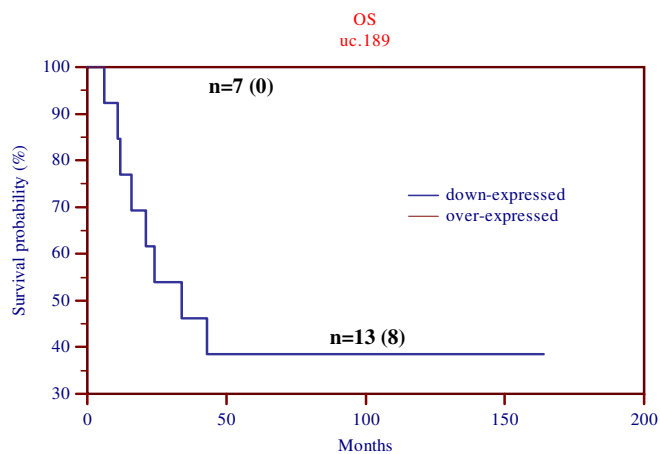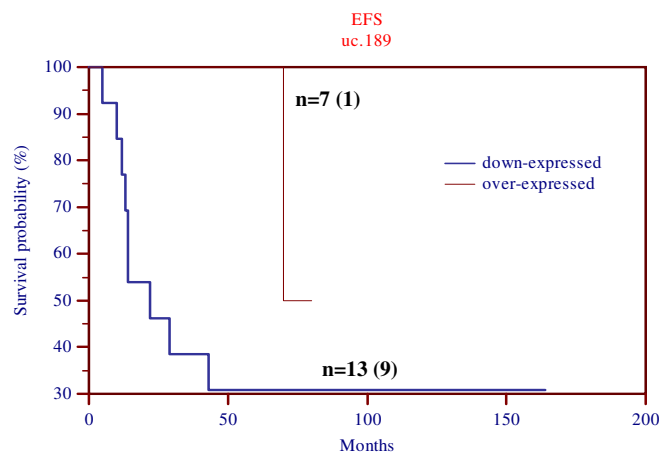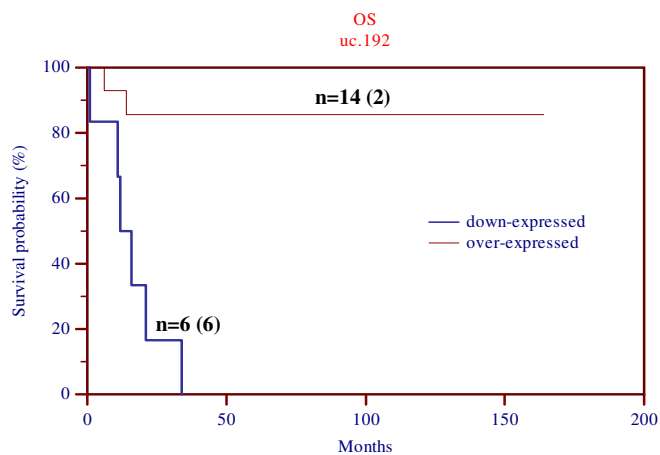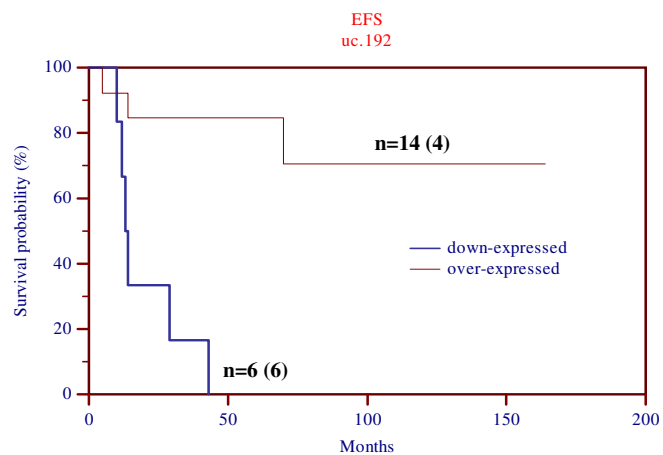

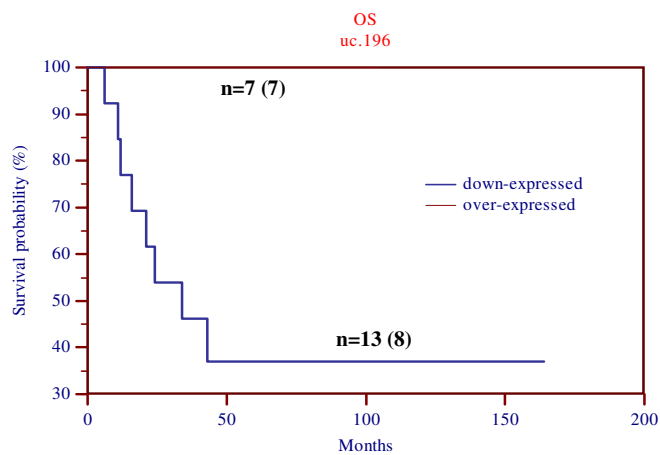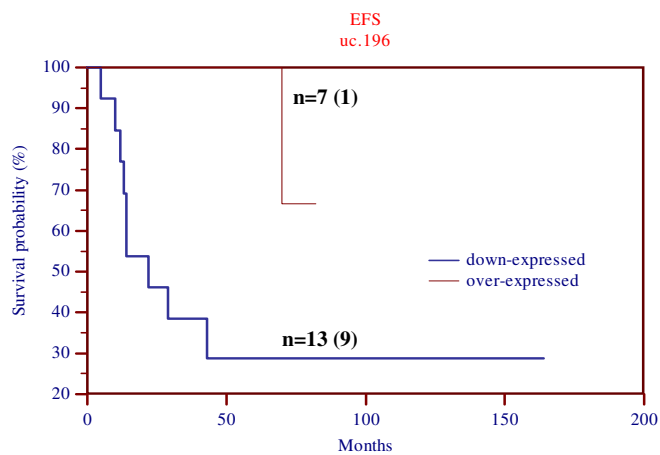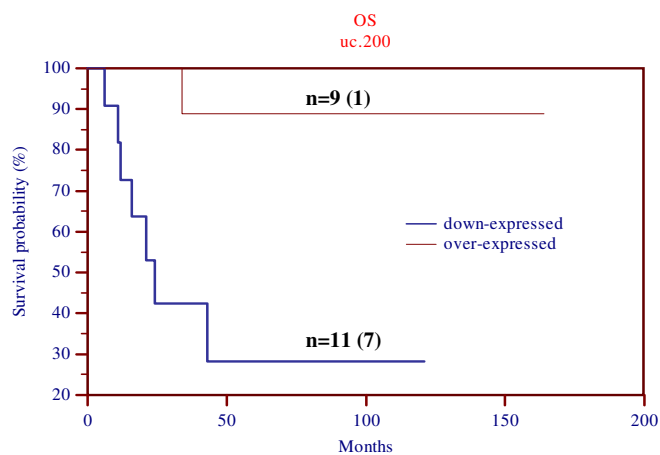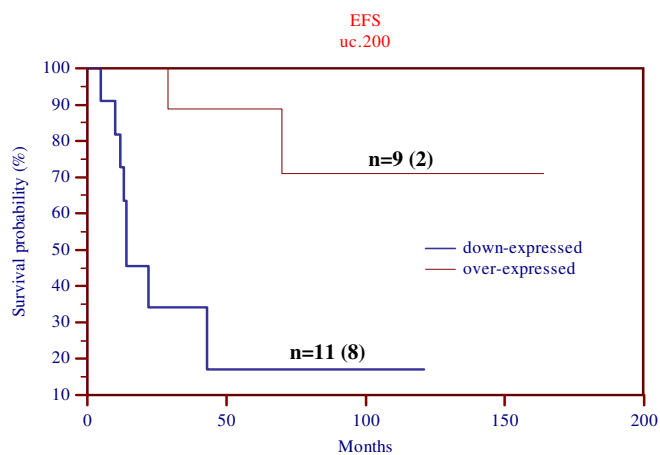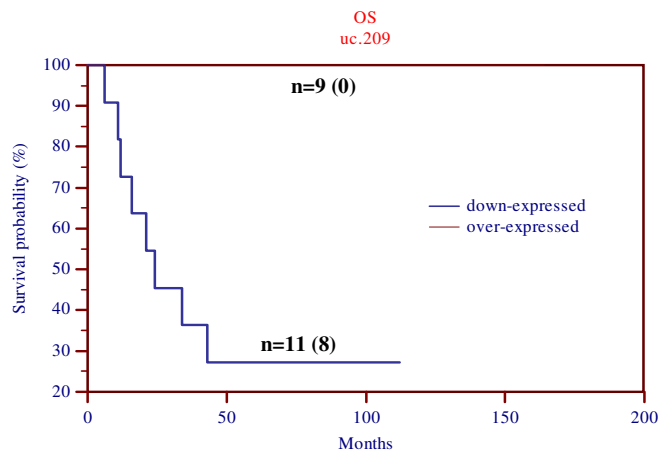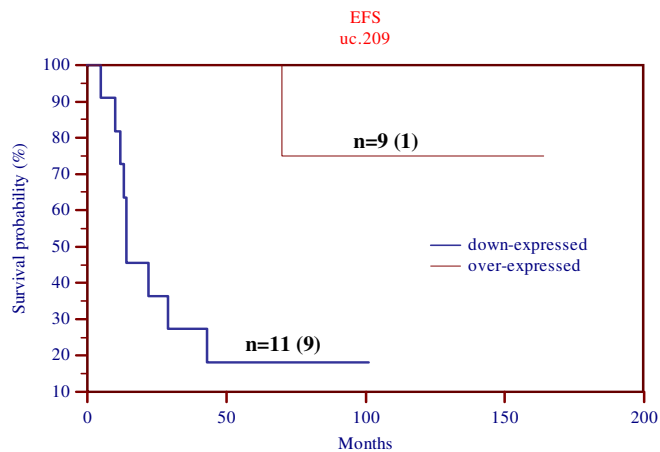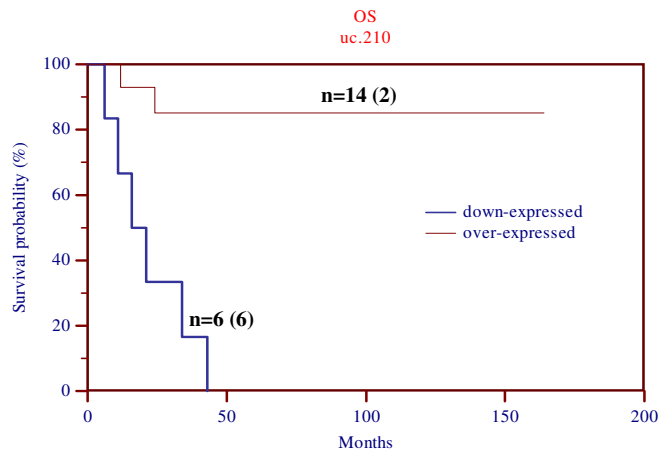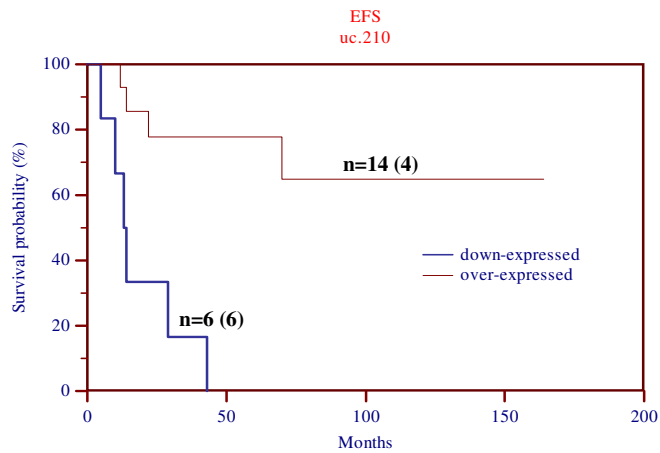

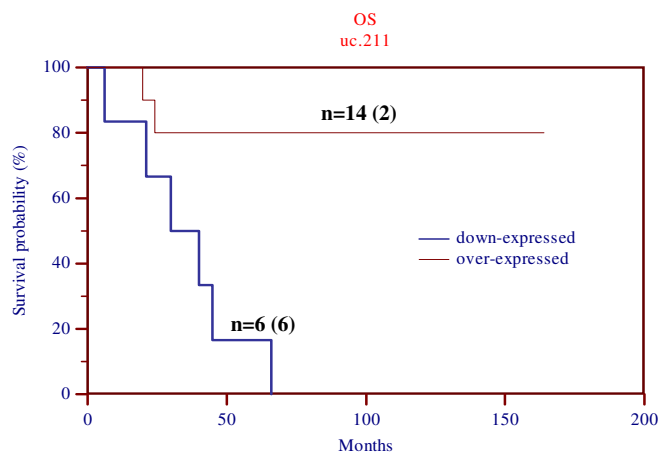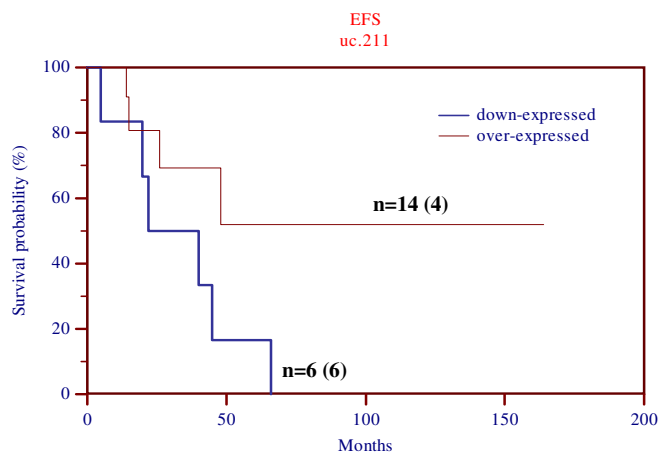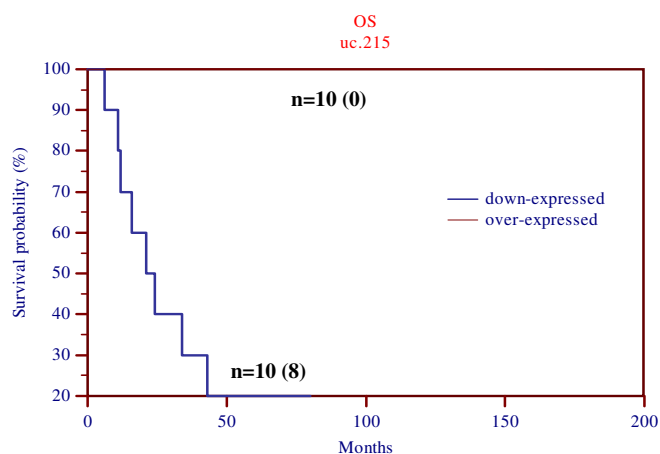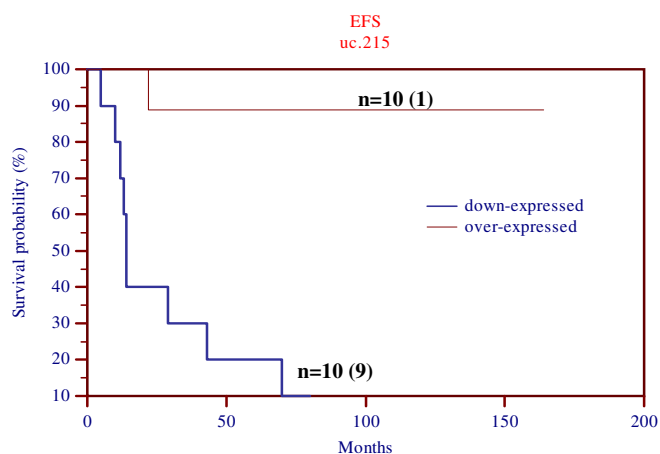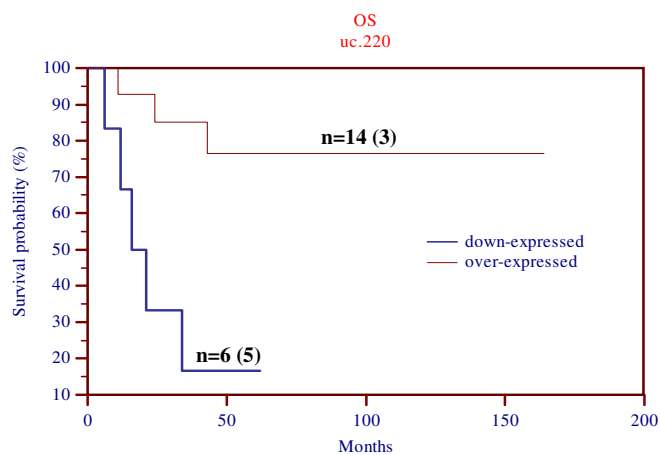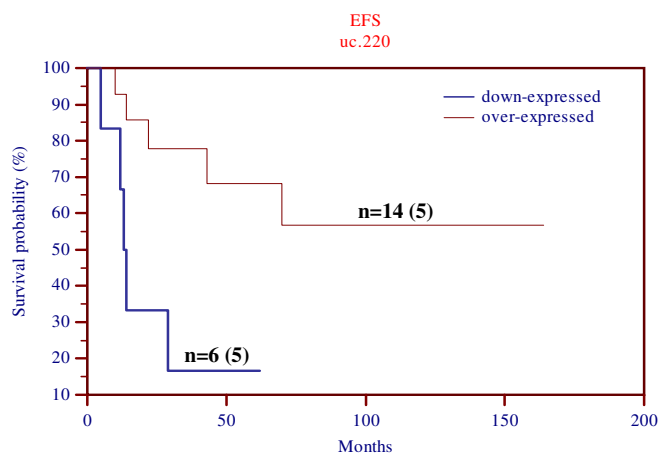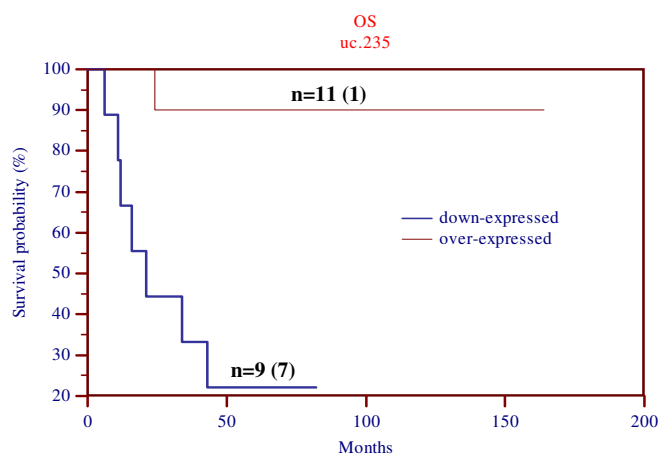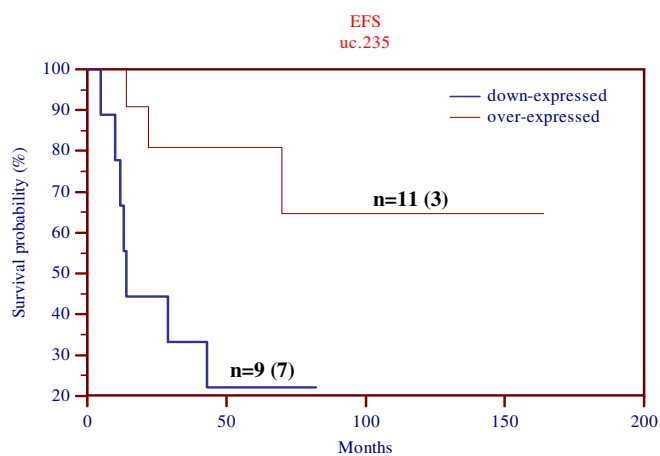

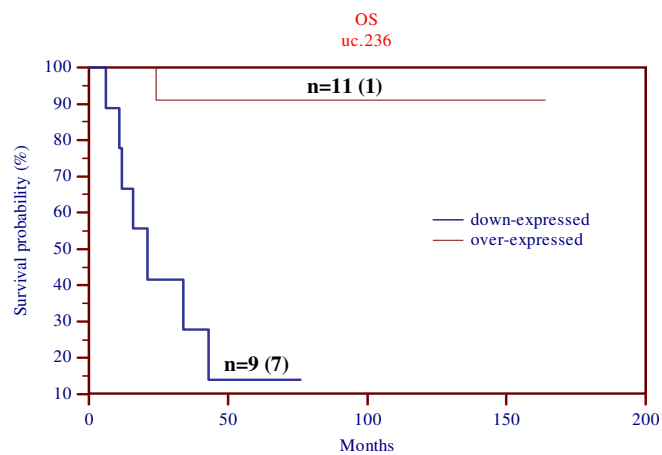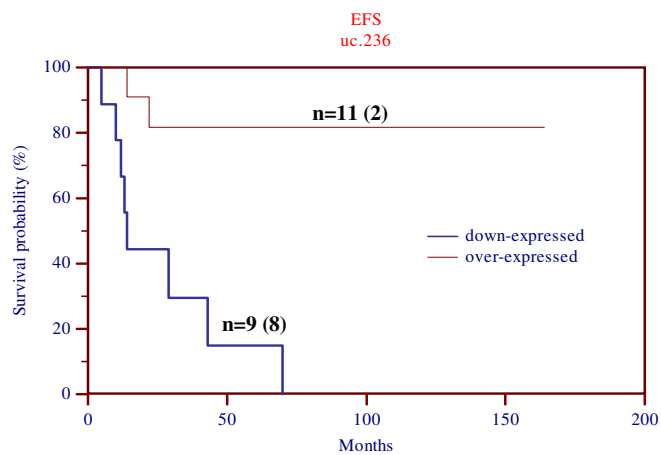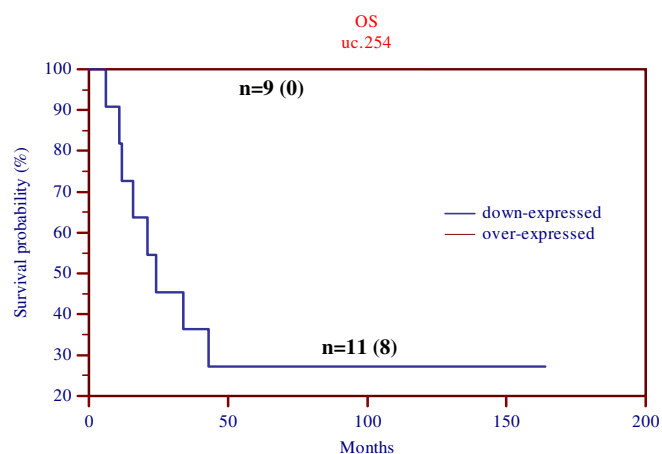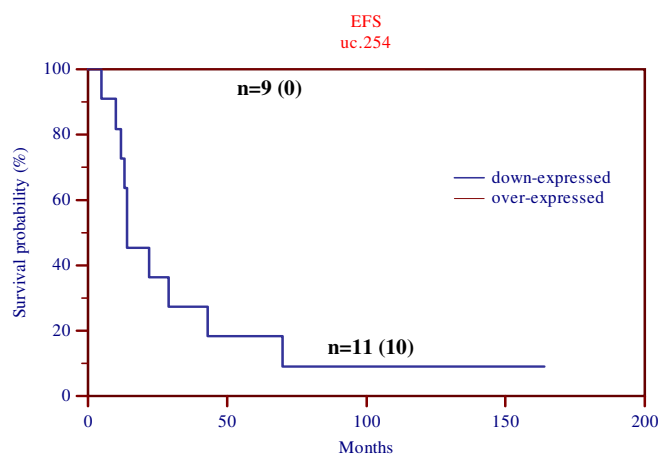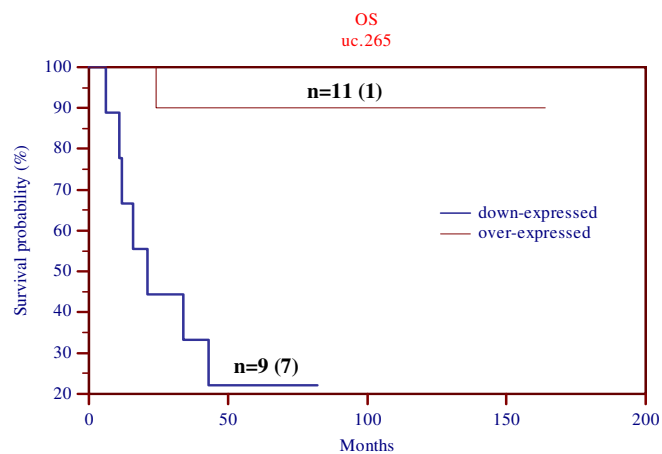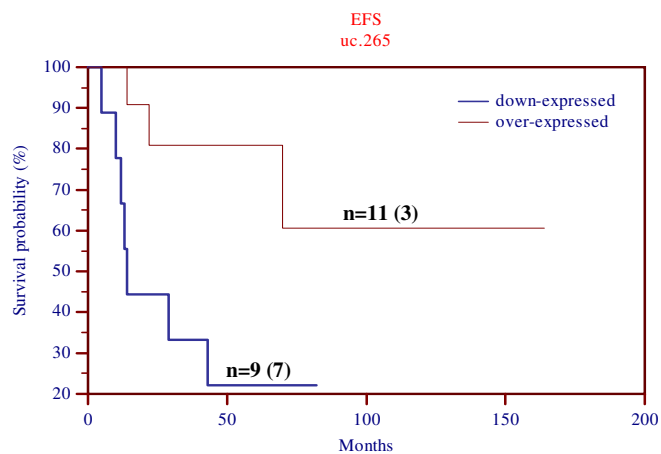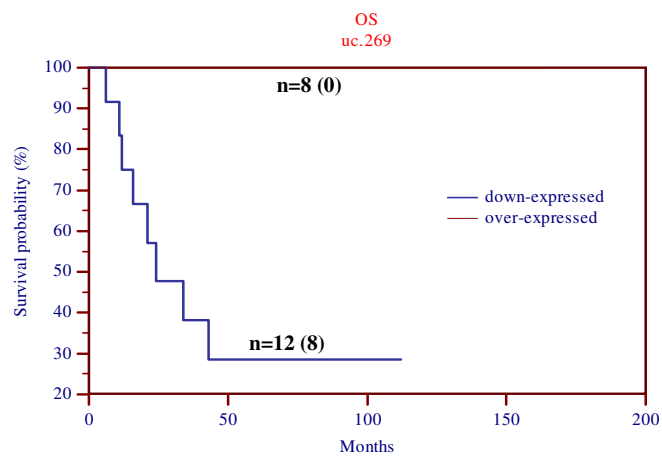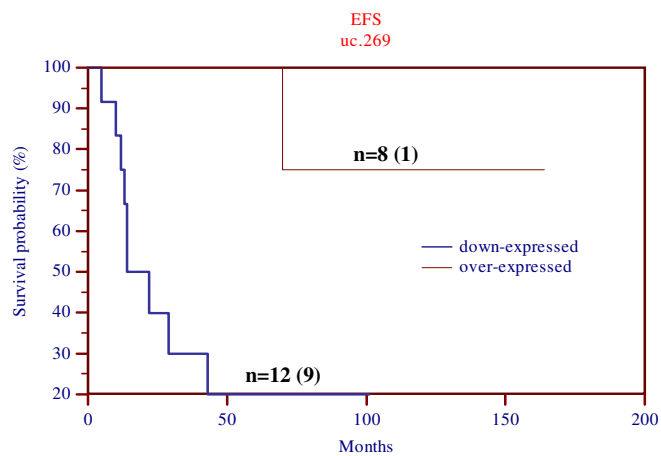

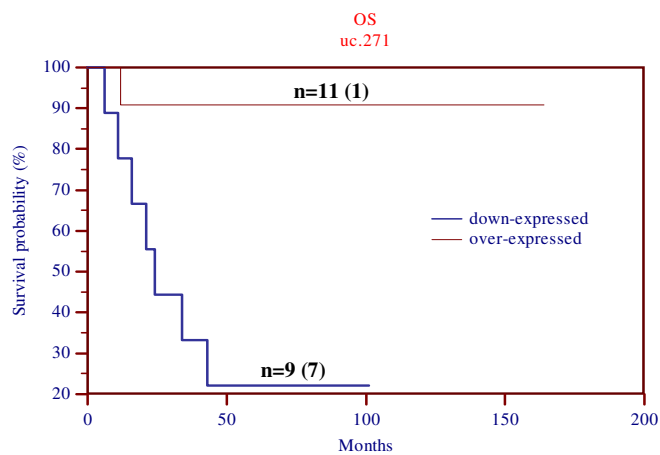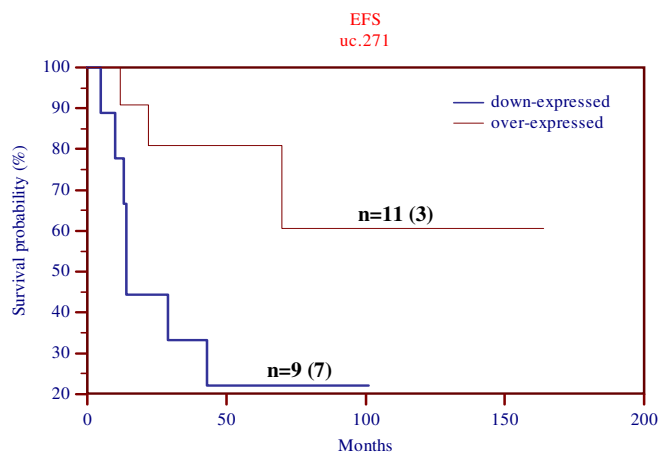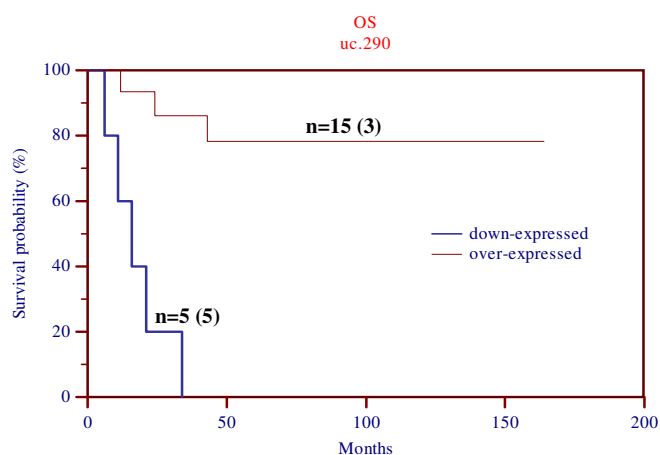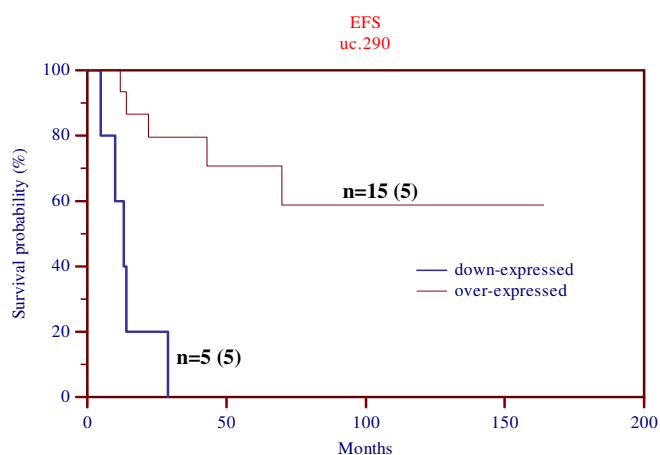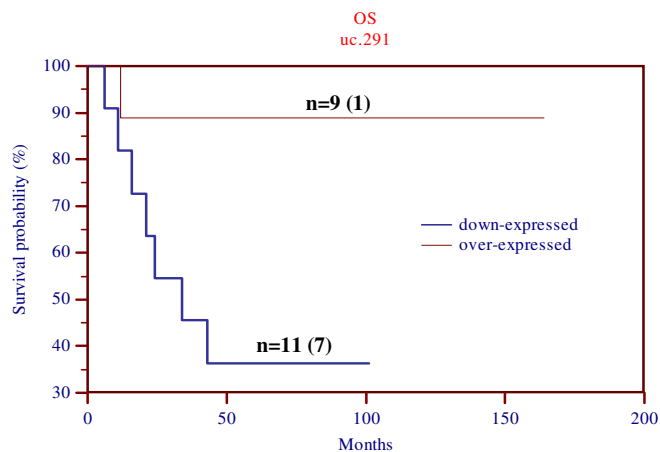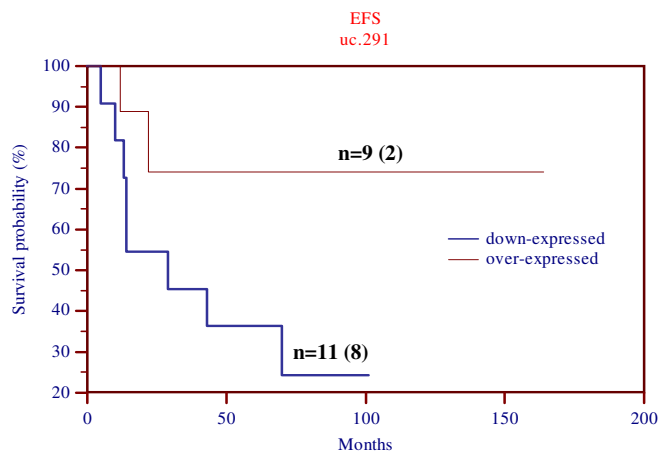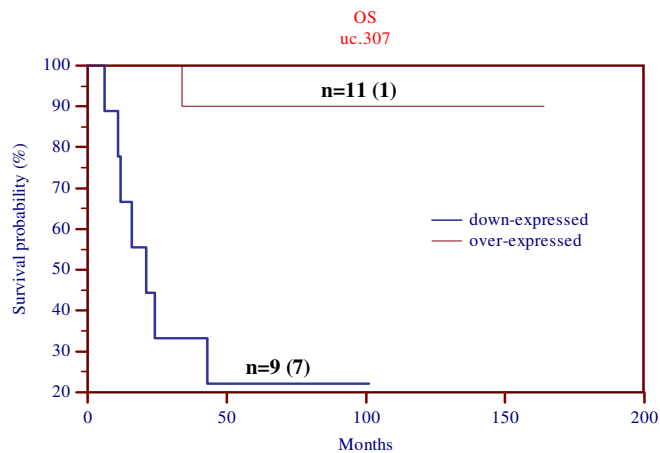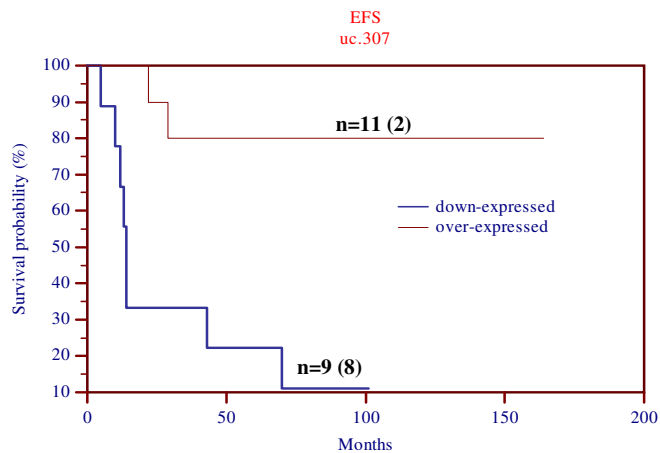

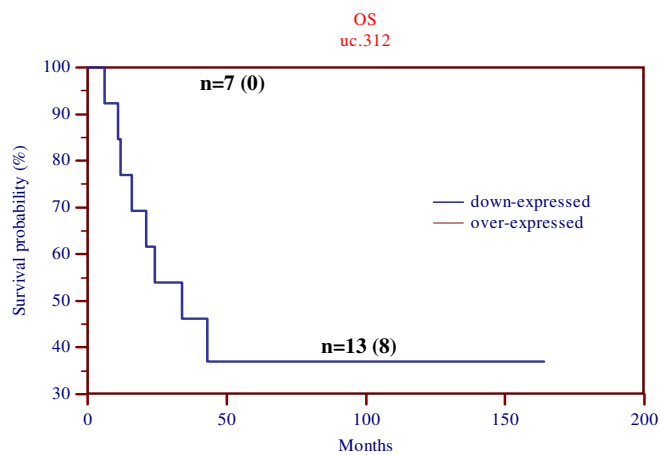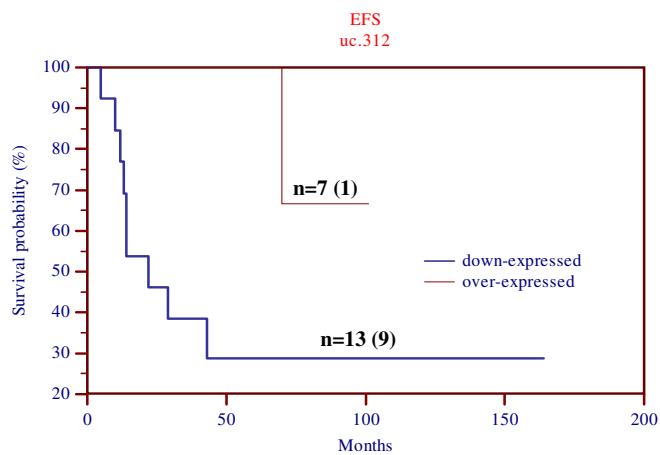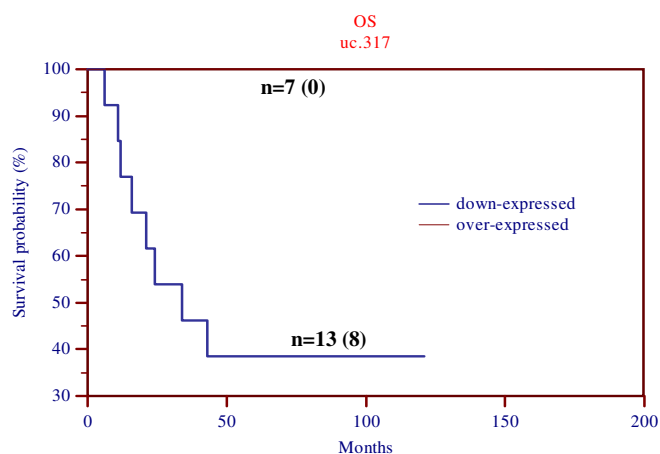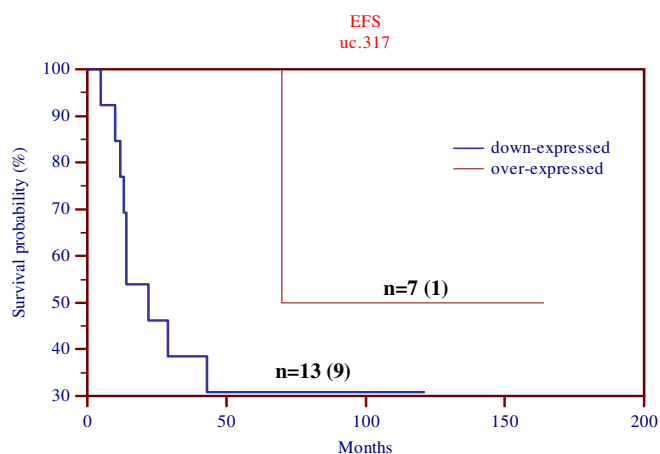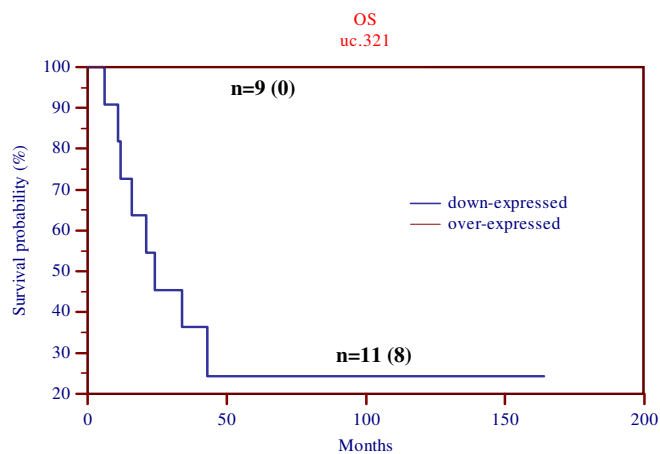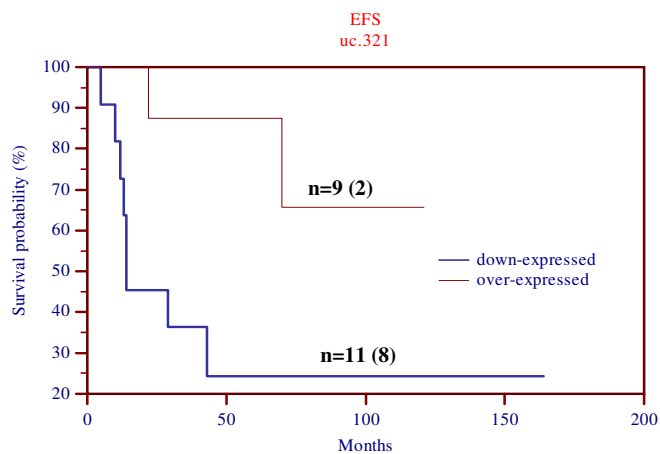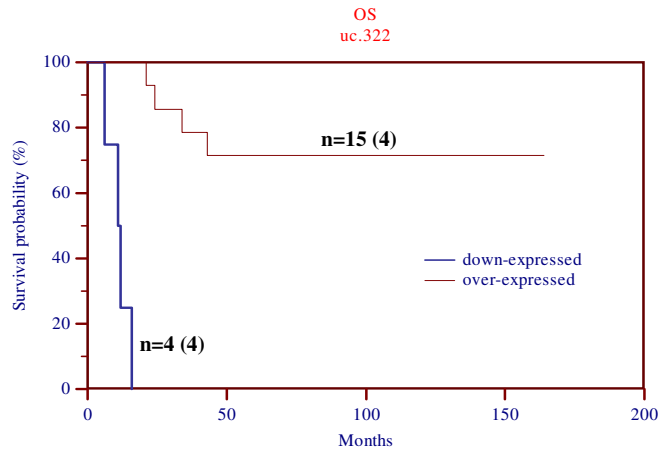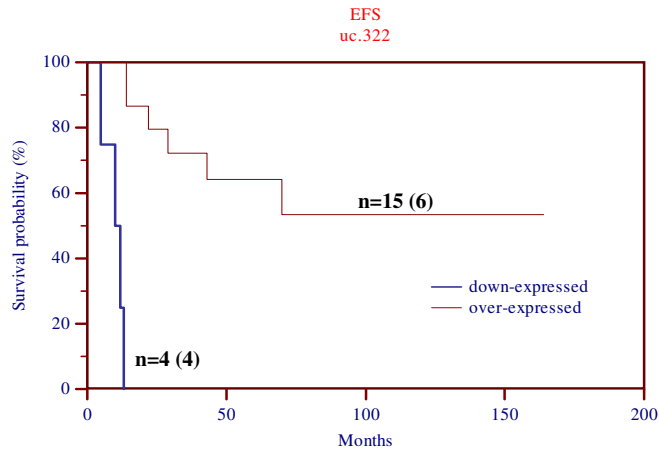

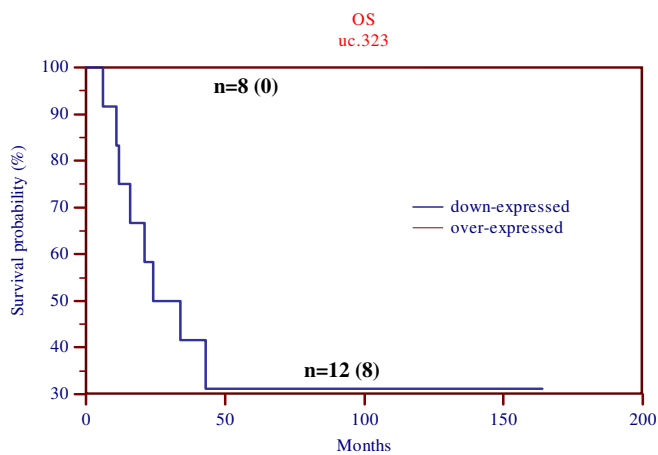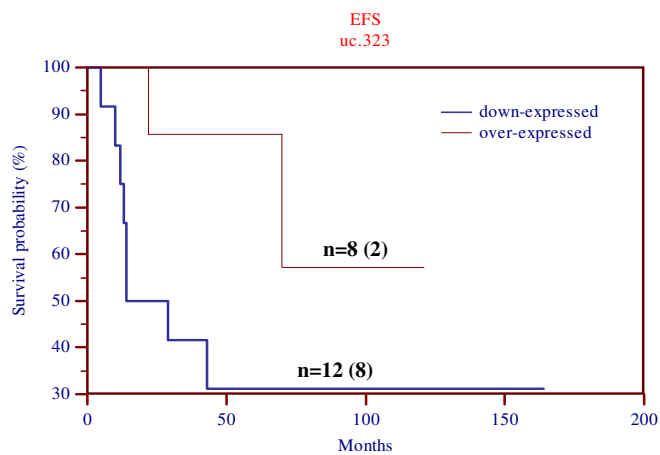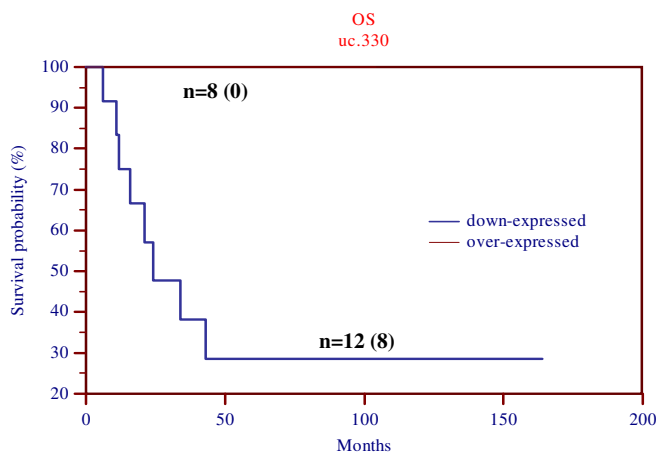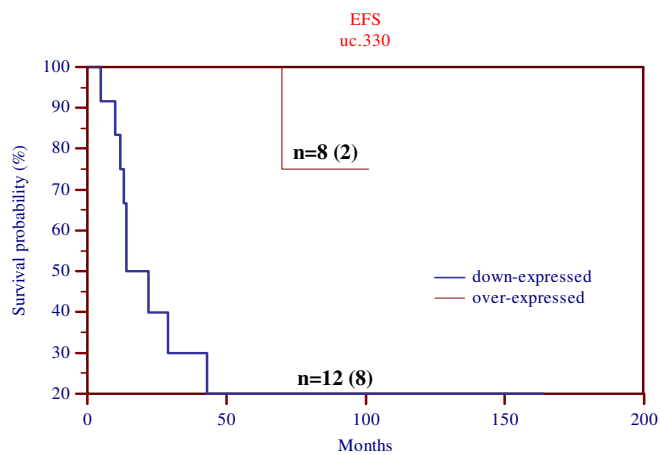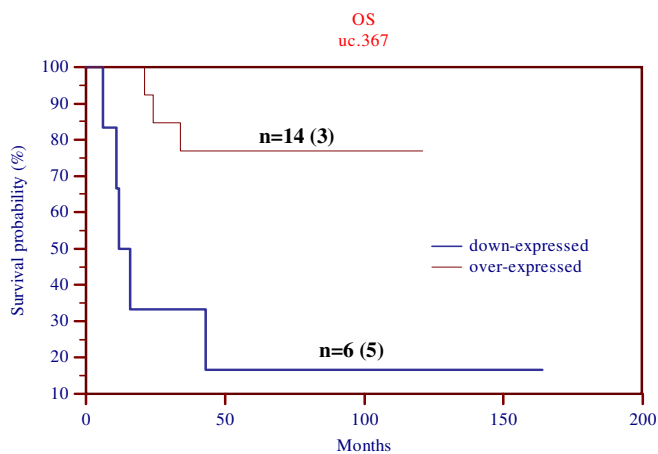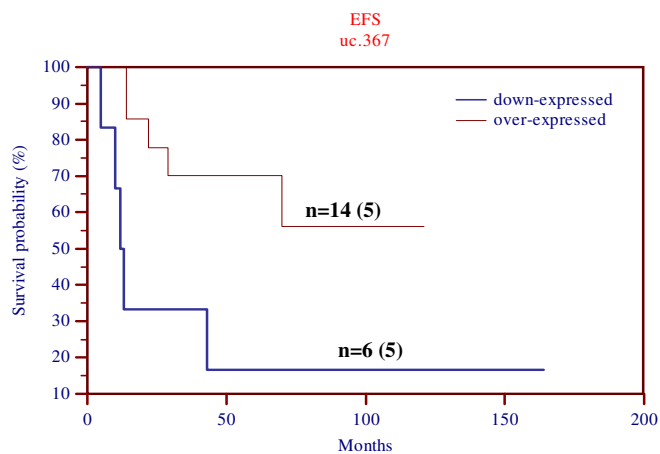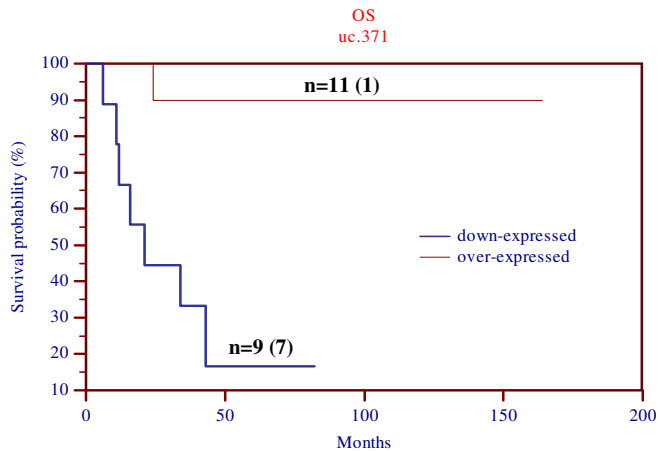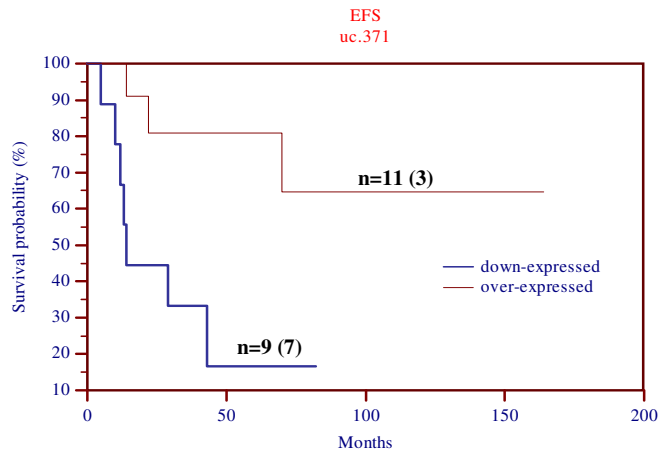

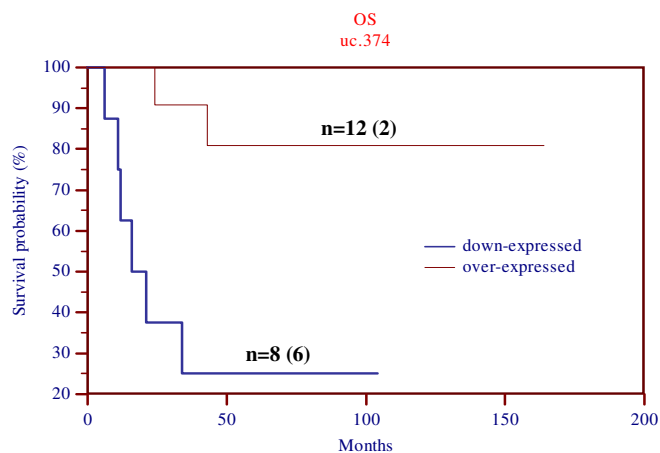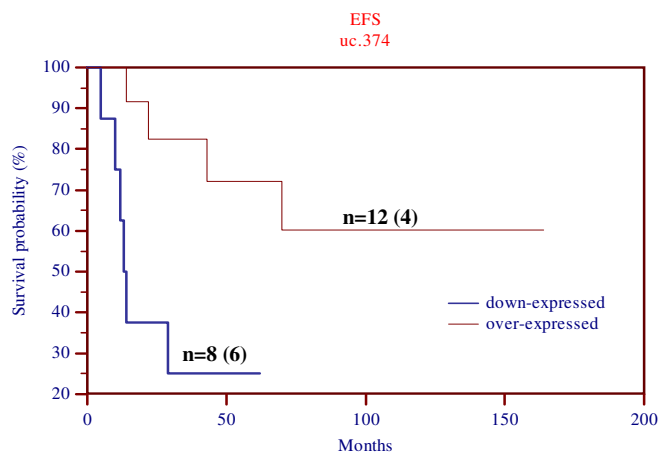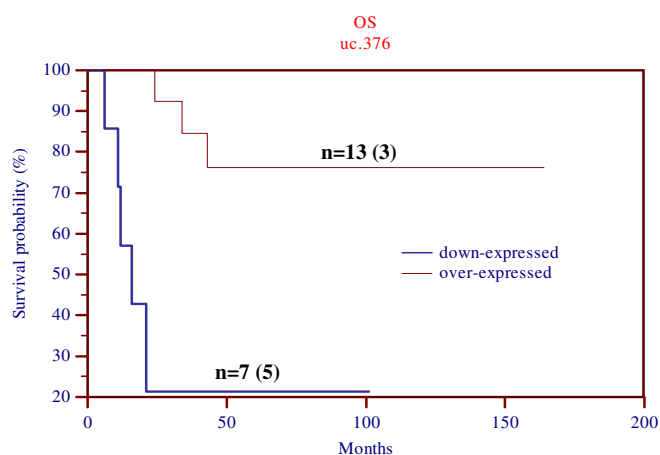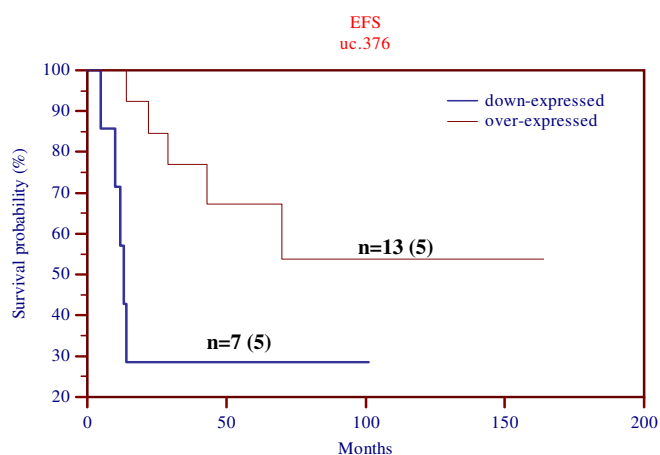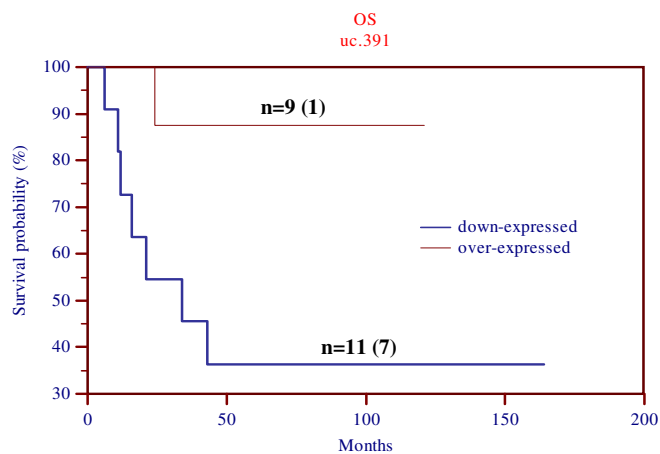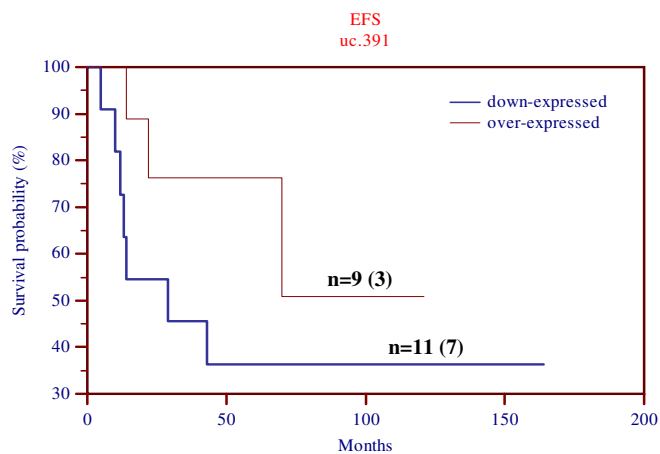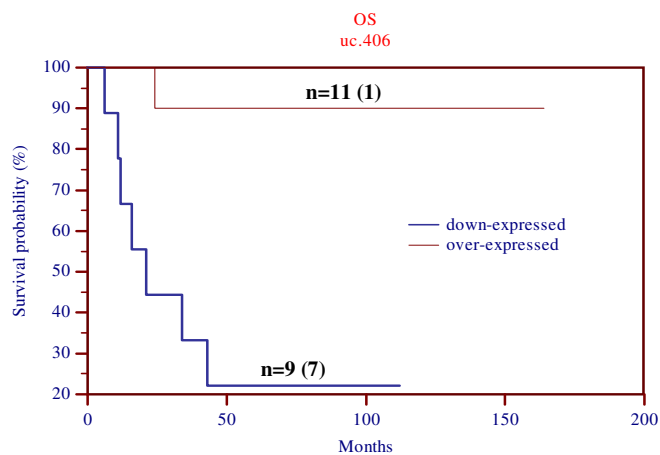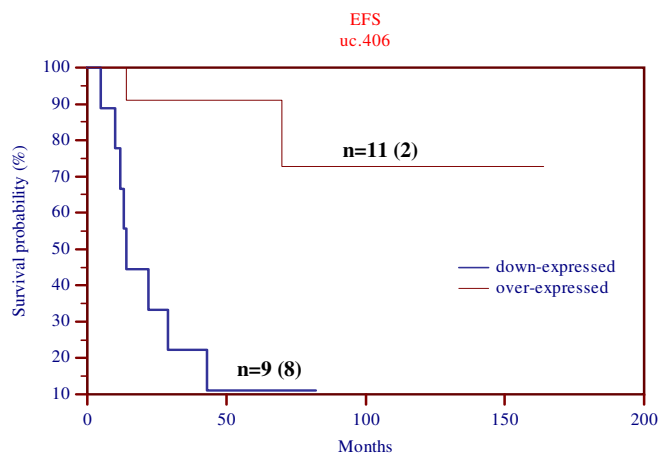

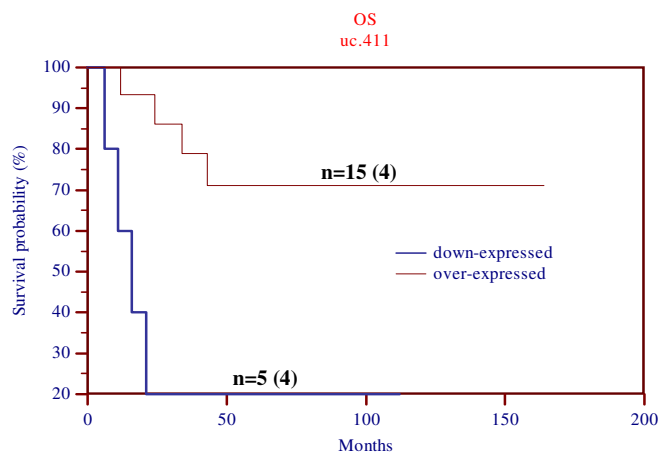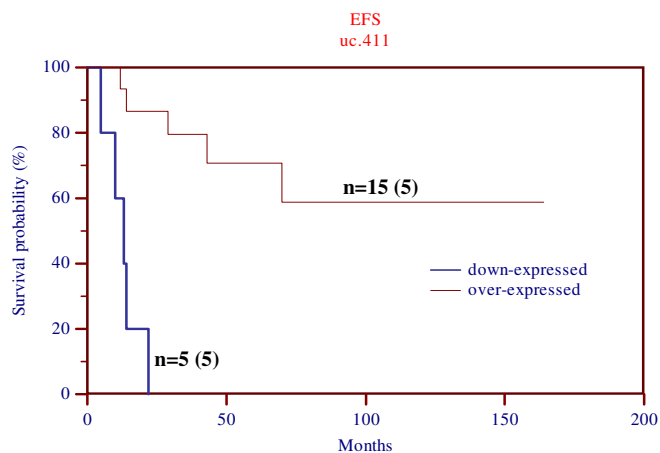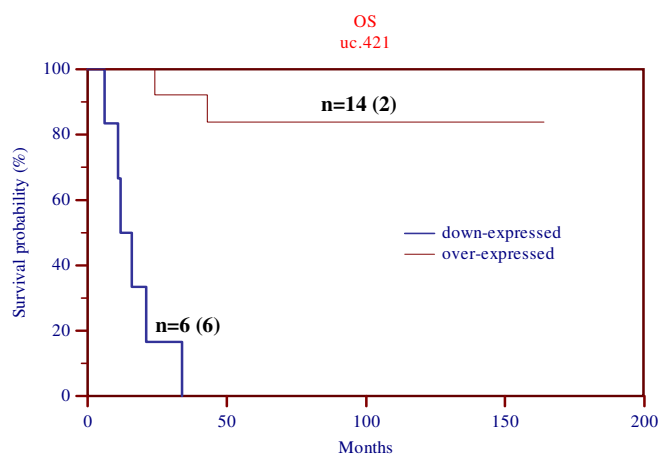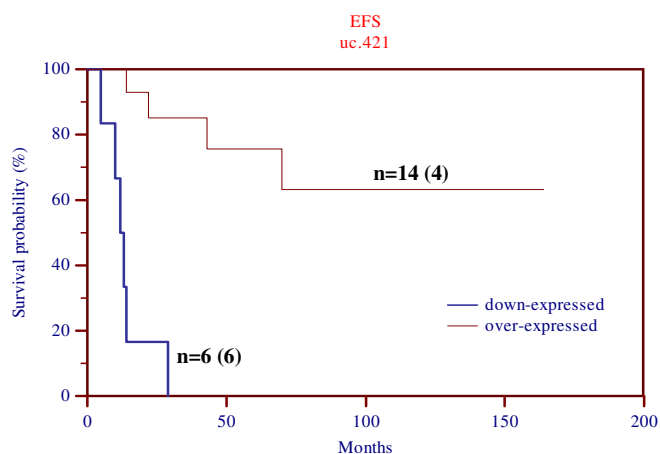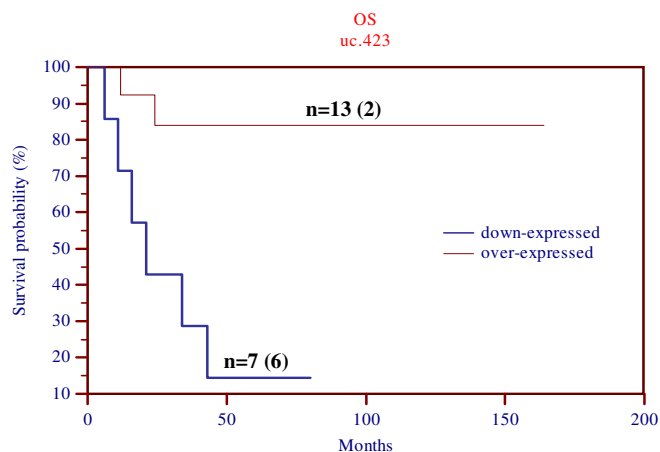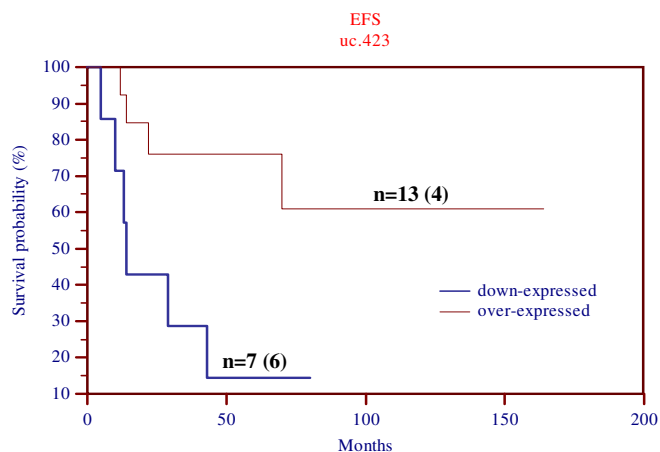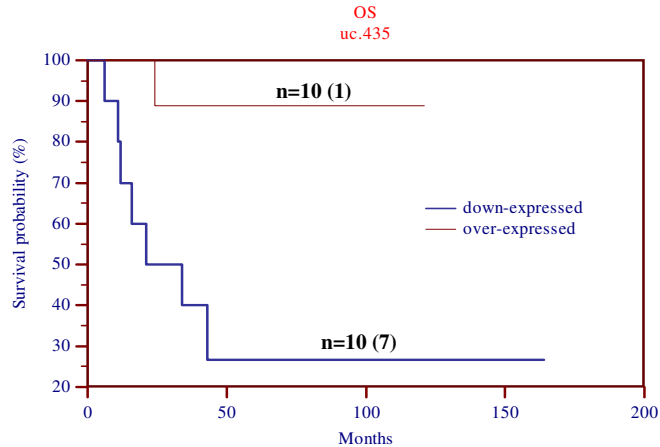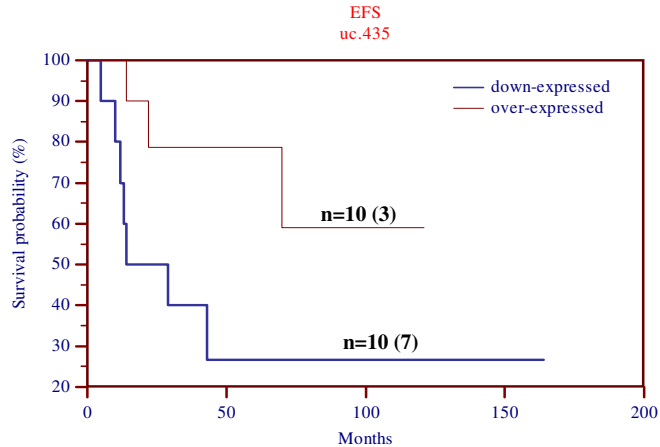

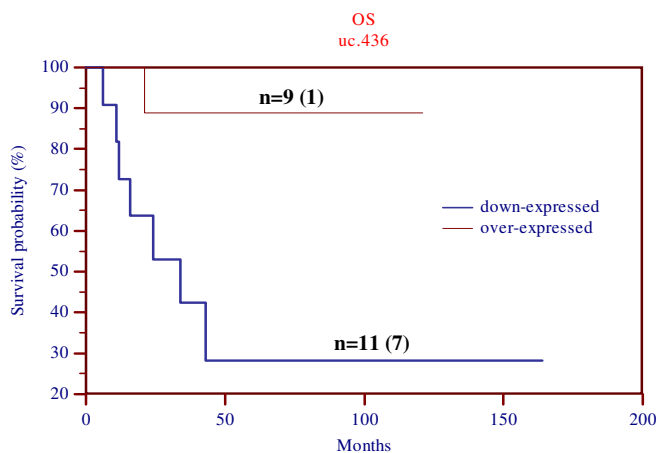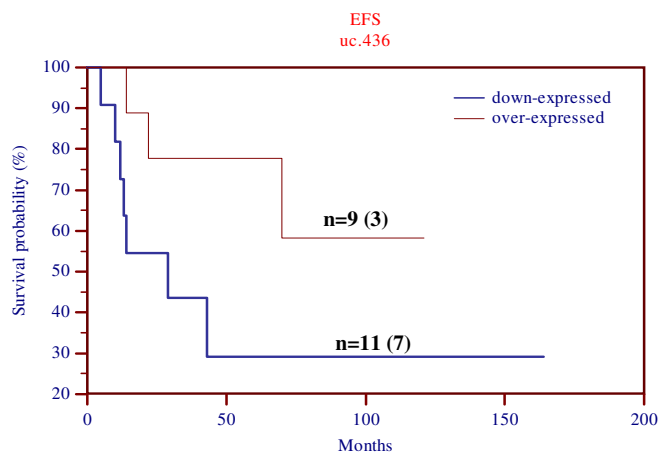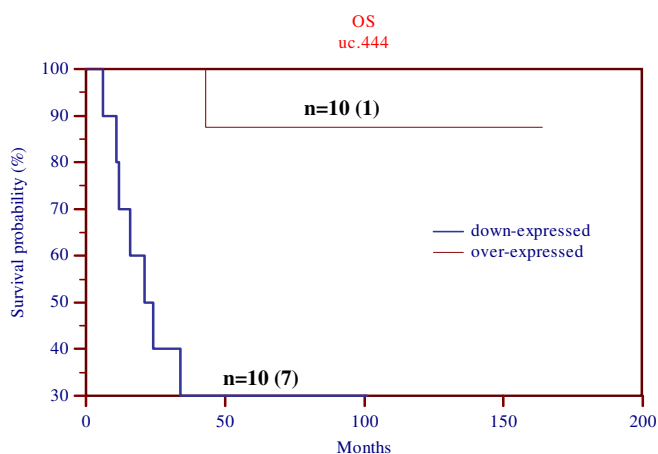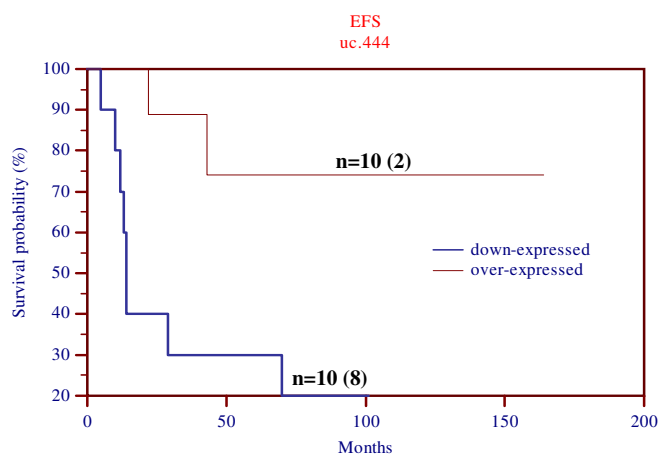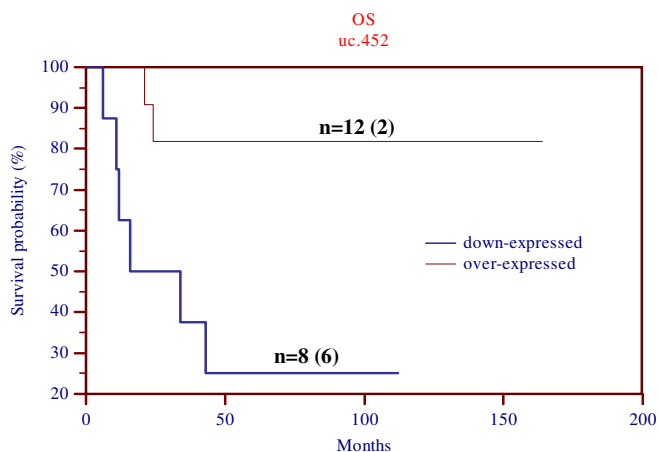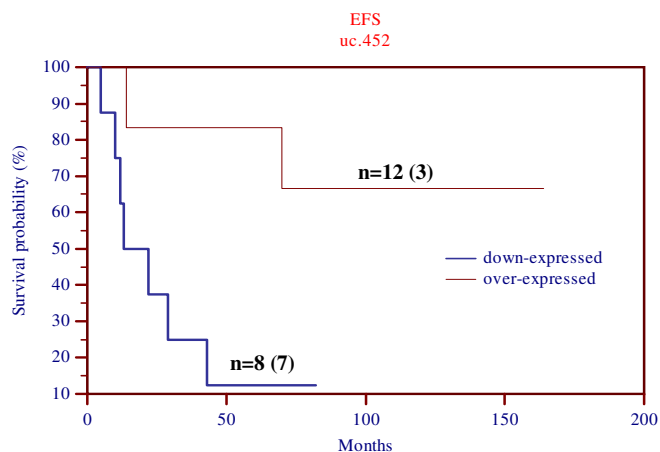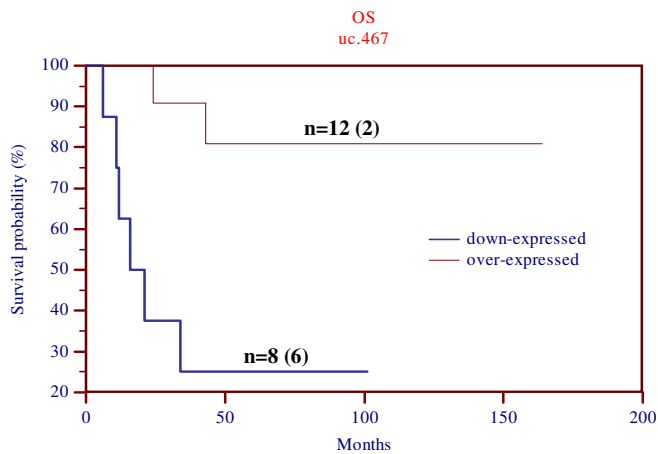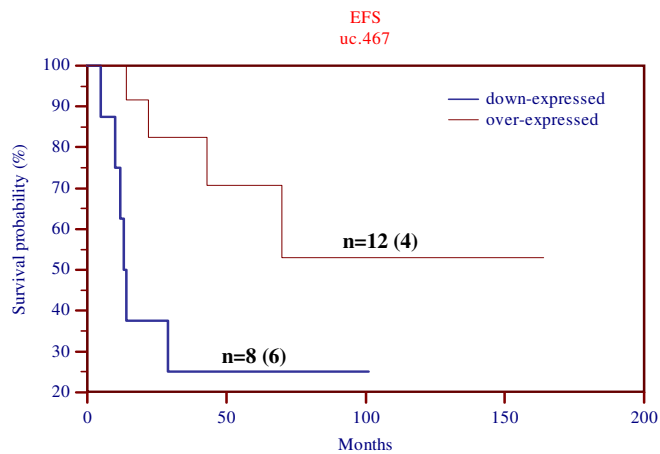

Supplement: Additional file 7 — Figure S3. Kaplan-Meier overall and event-free survival curves of the first set of 20 NB patients categorized according to T-UCR expression values (above or below the threshold defined by the respective ROC curve). In each graph are reported the number of patients in predicted subgroups and the number of patients with event (between brackets). [file 1471-2407-9-441-S7.PDF]

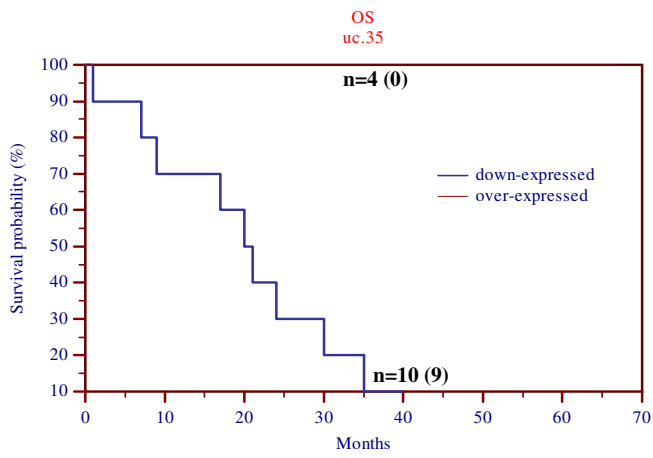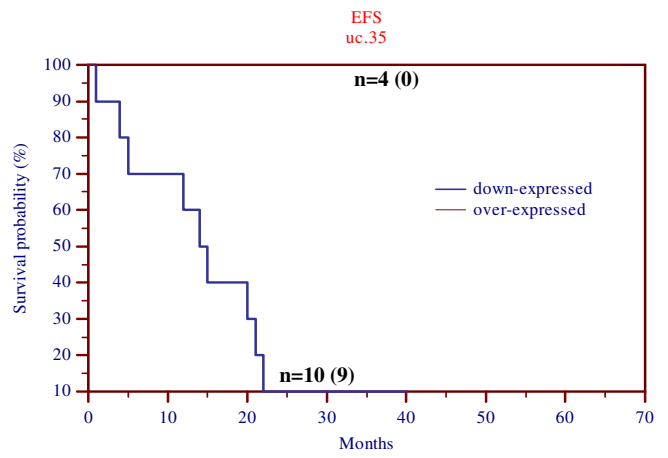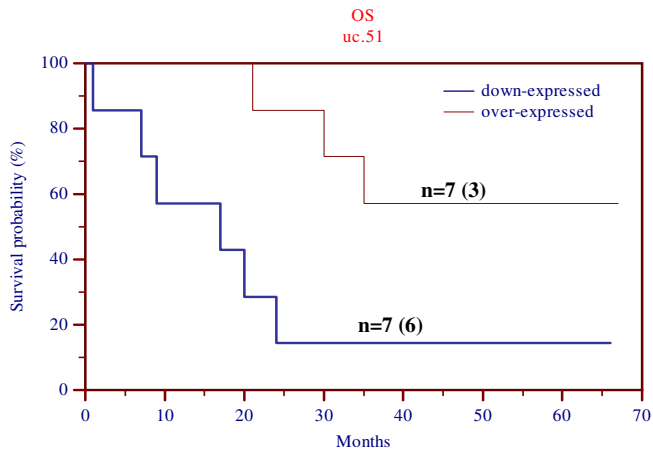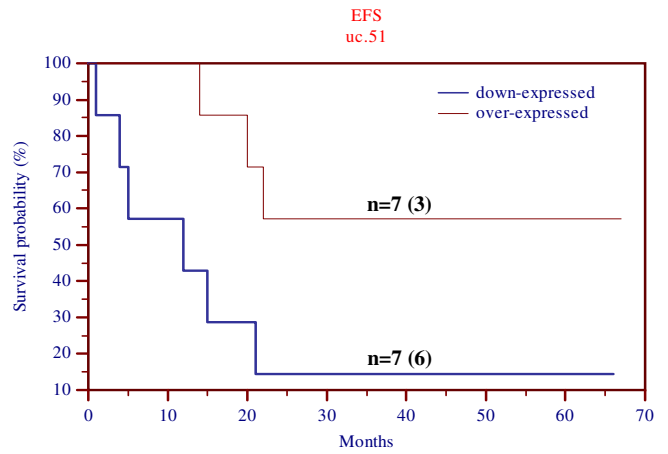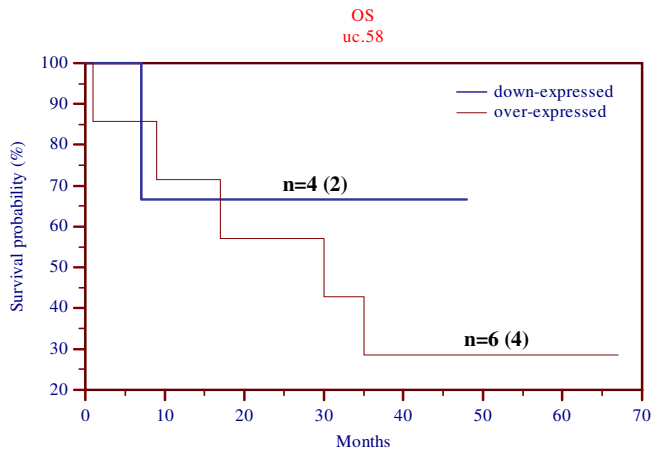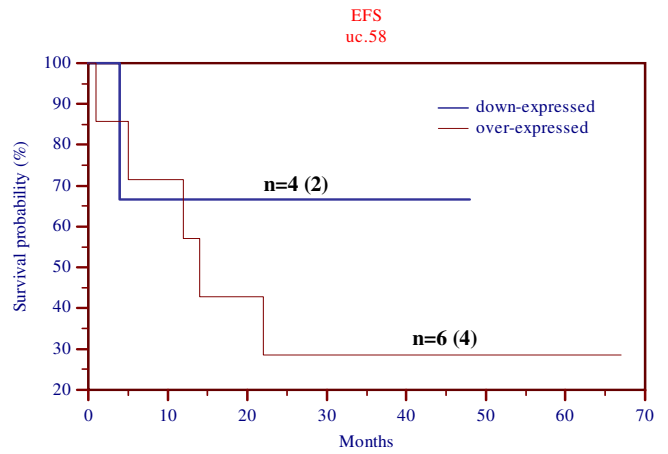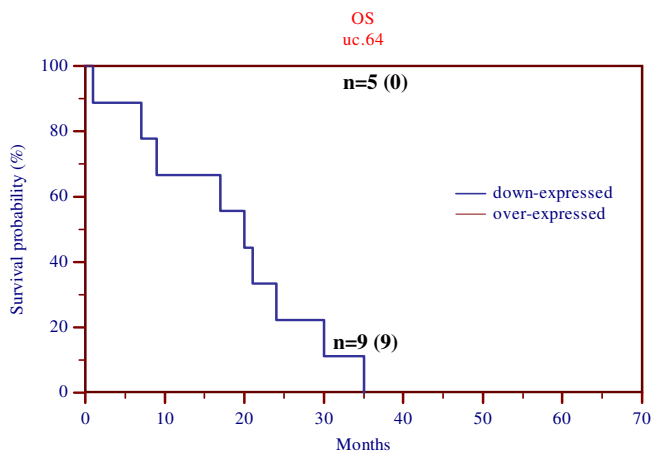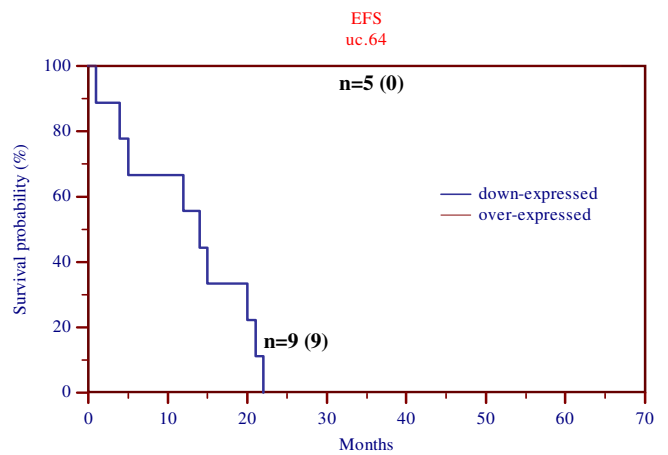

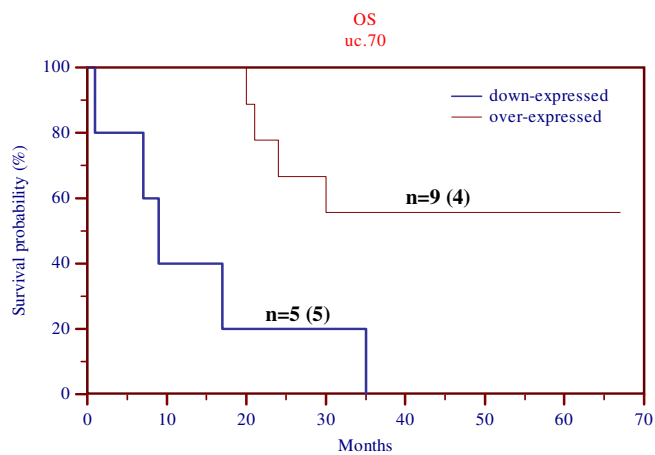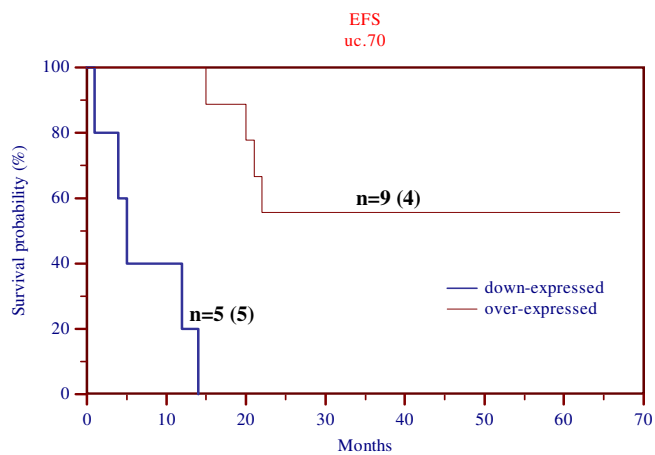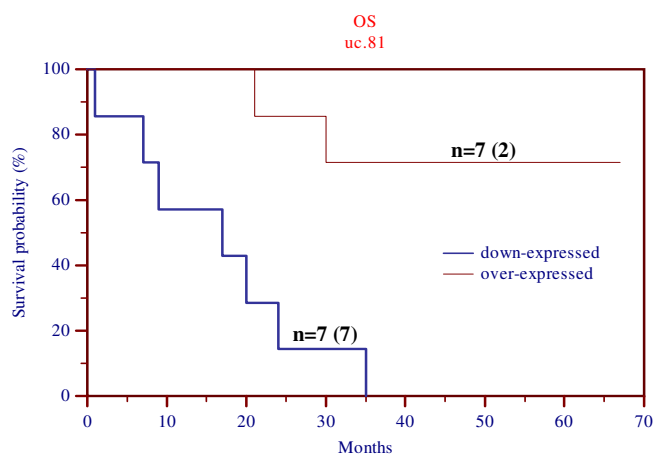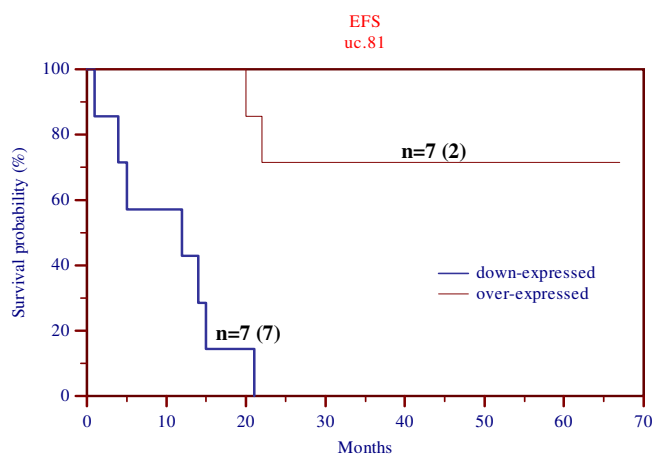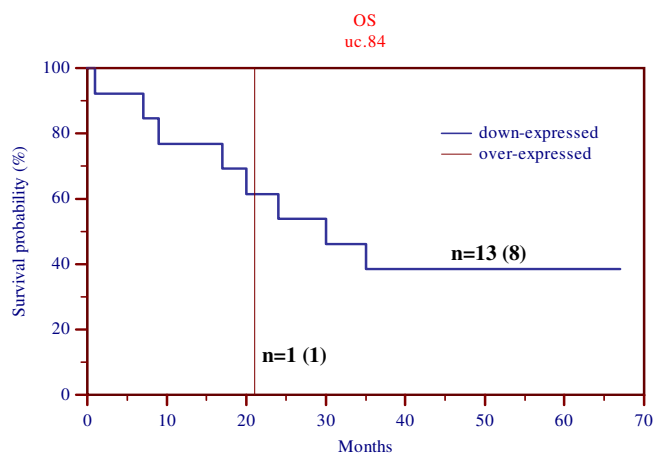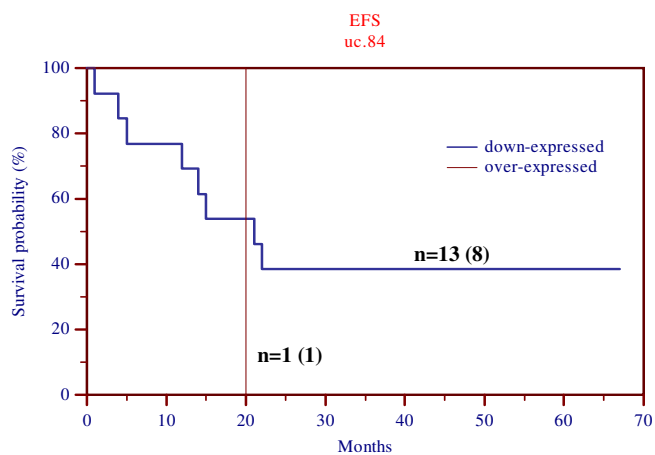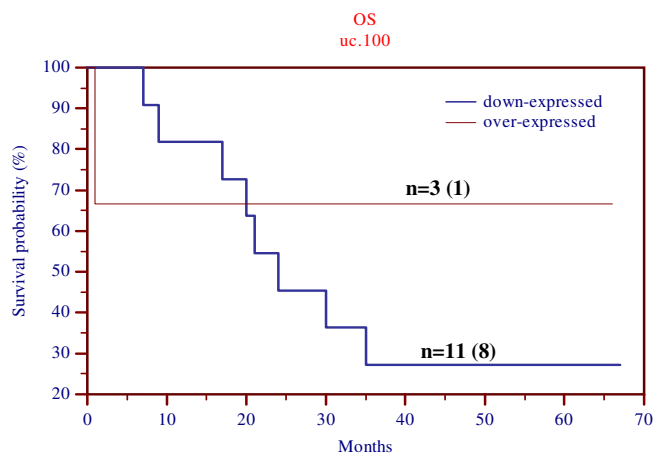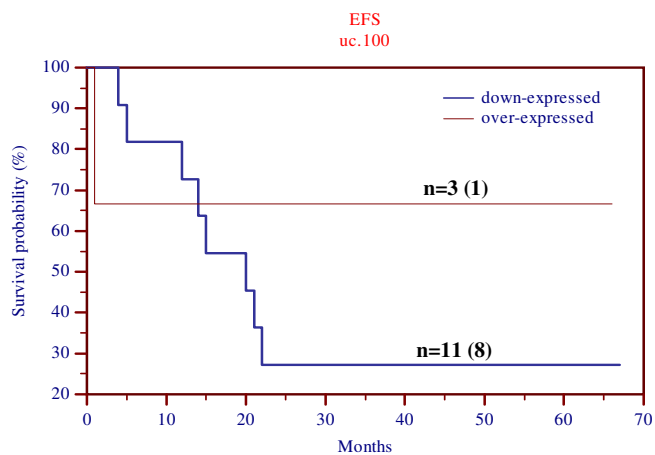

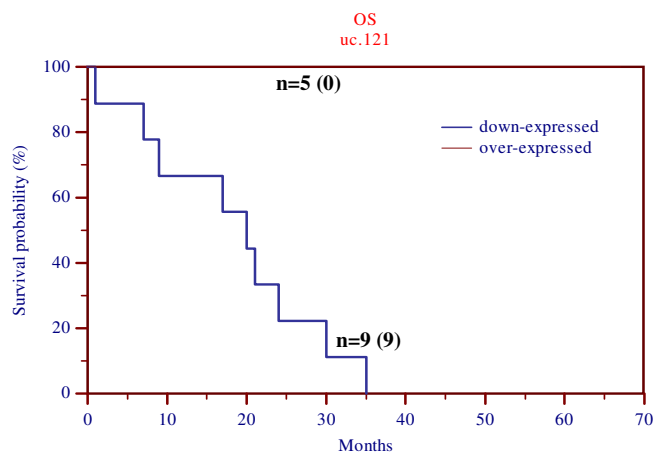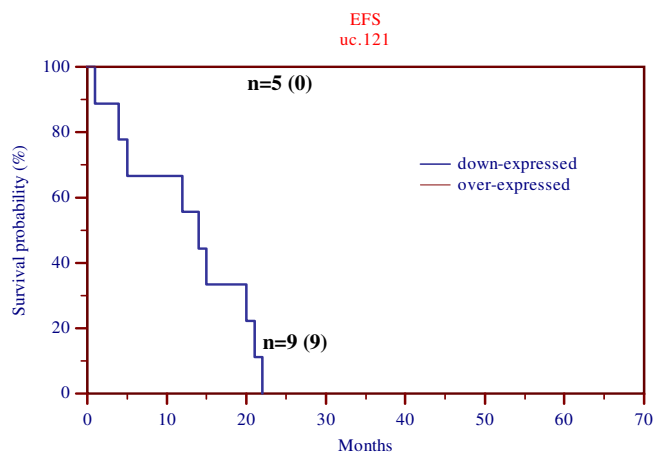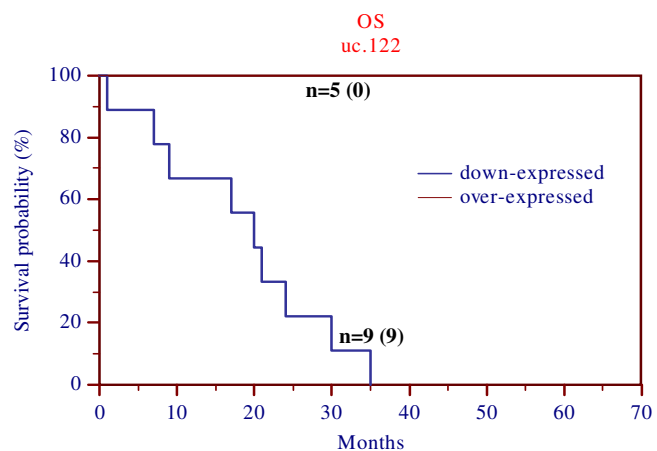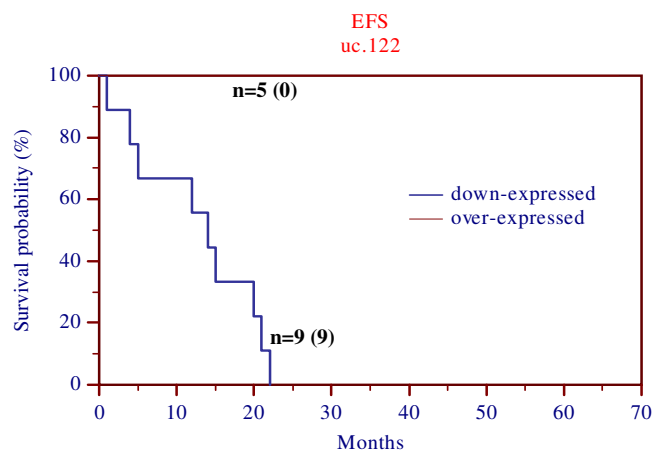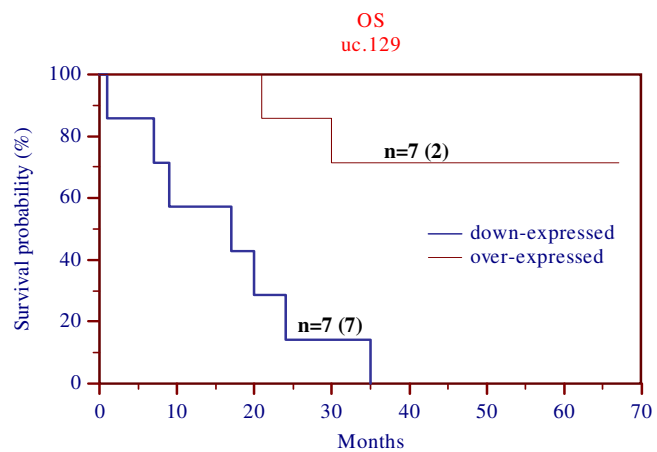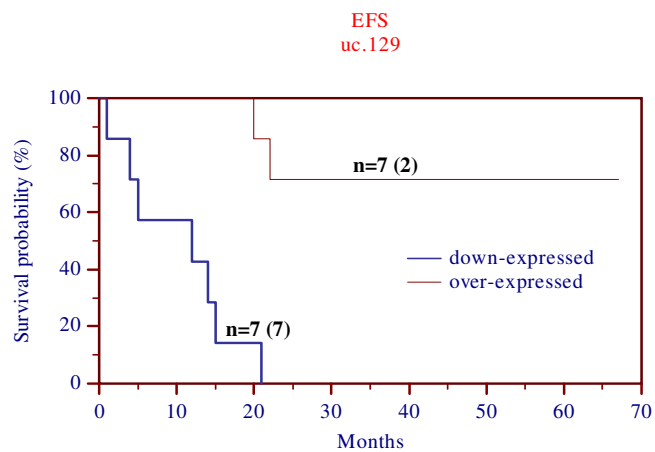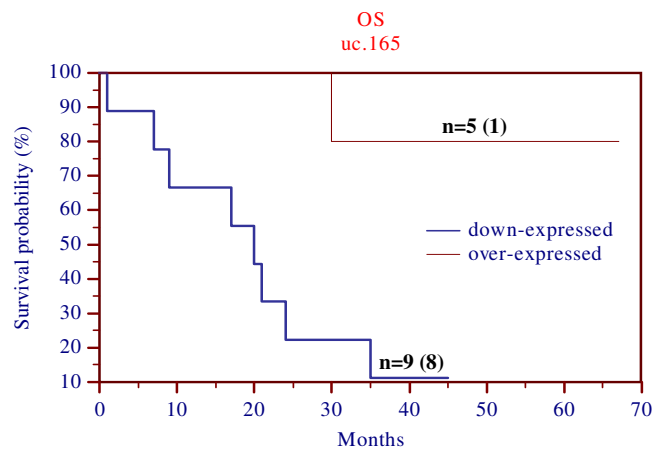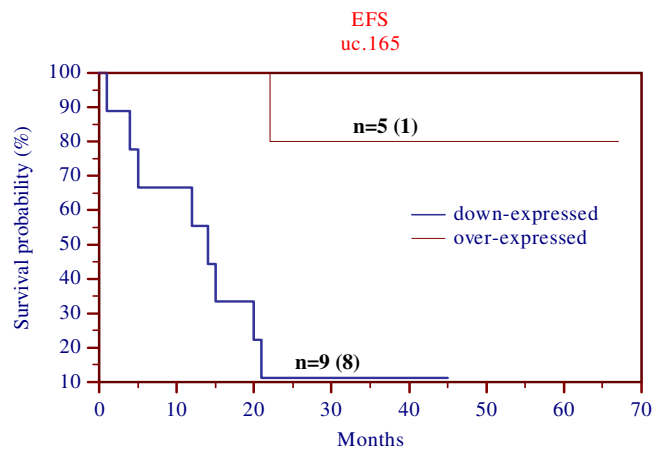

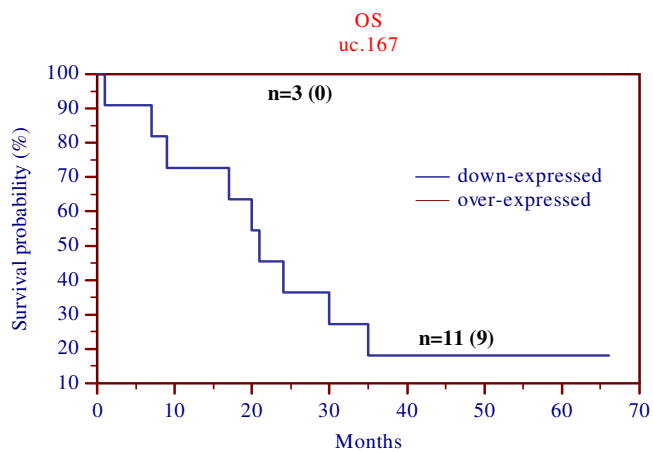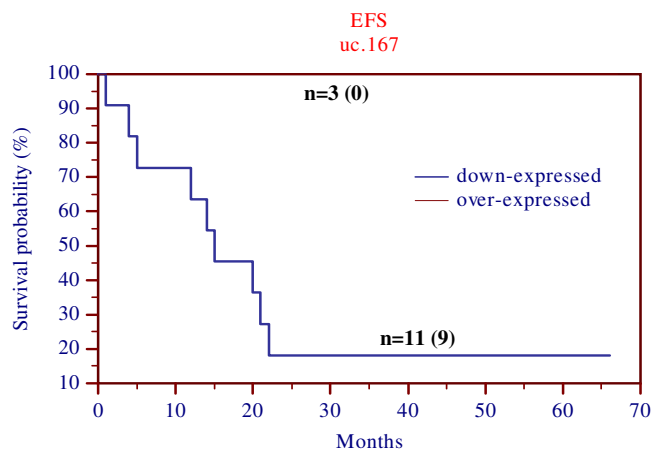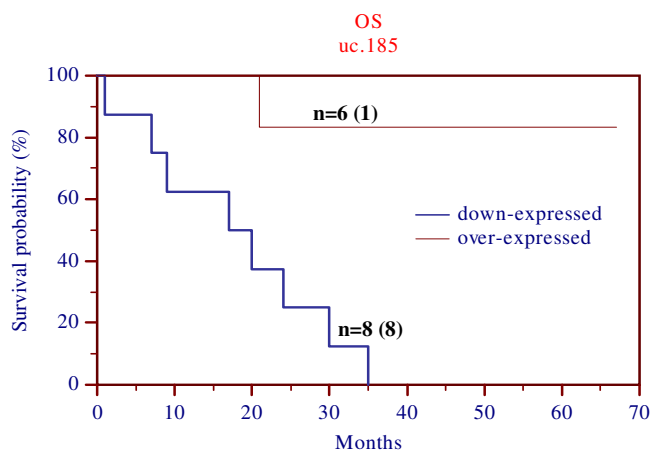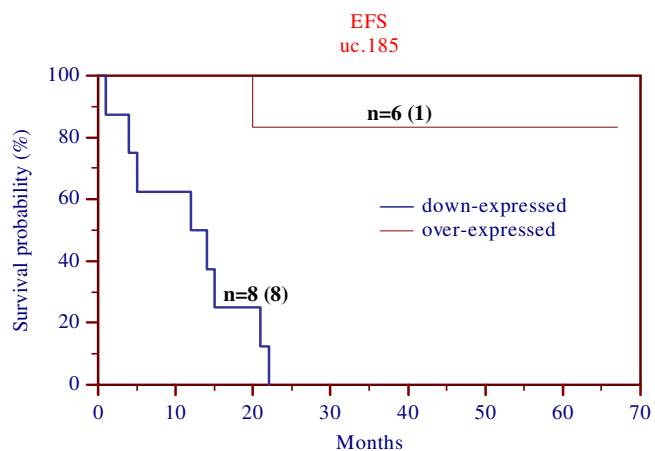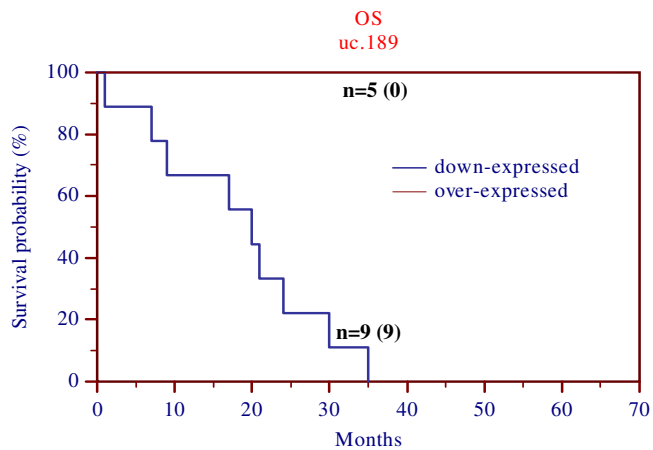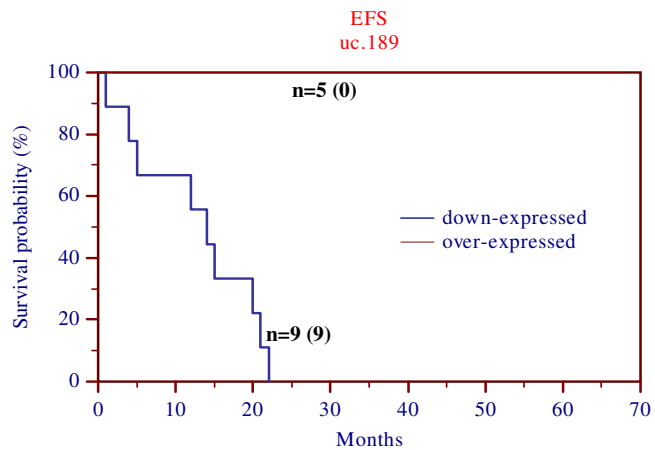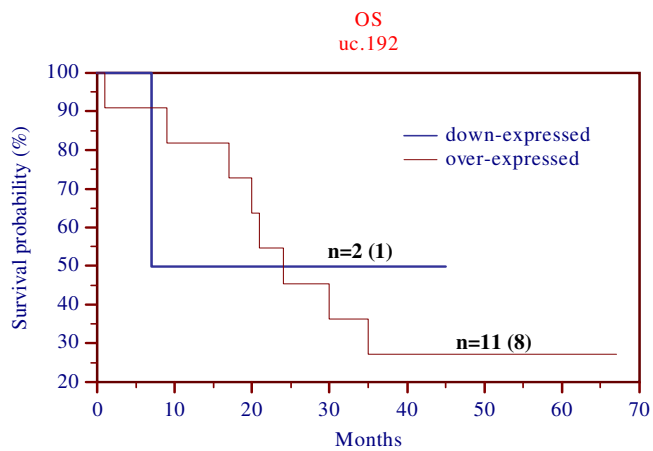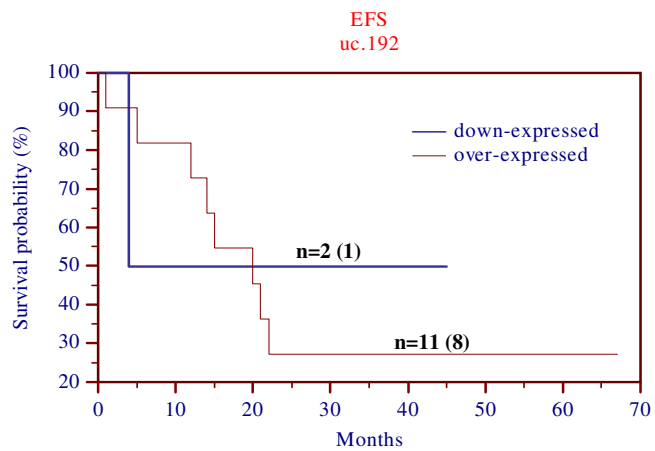

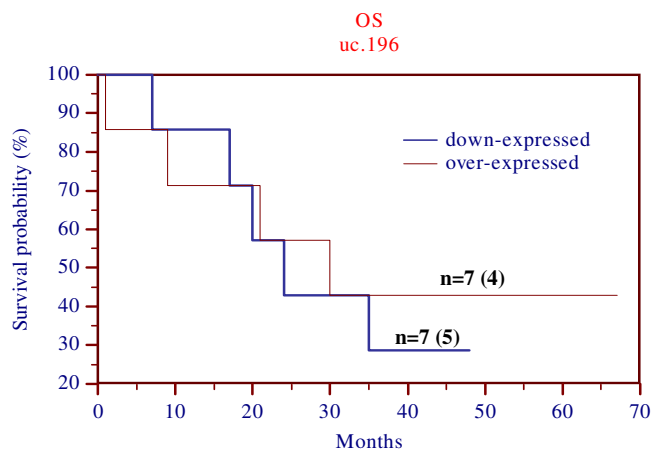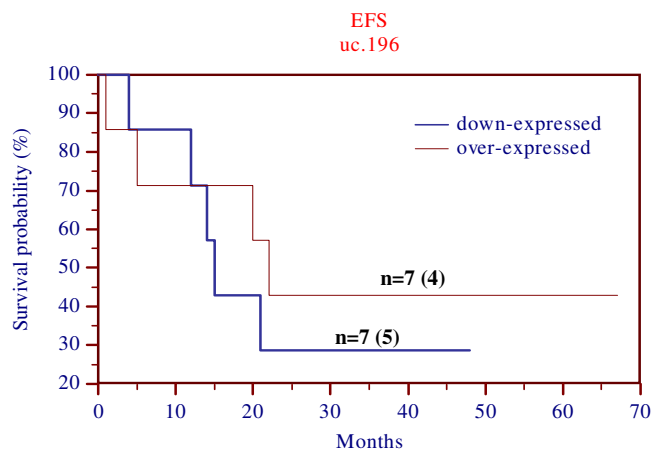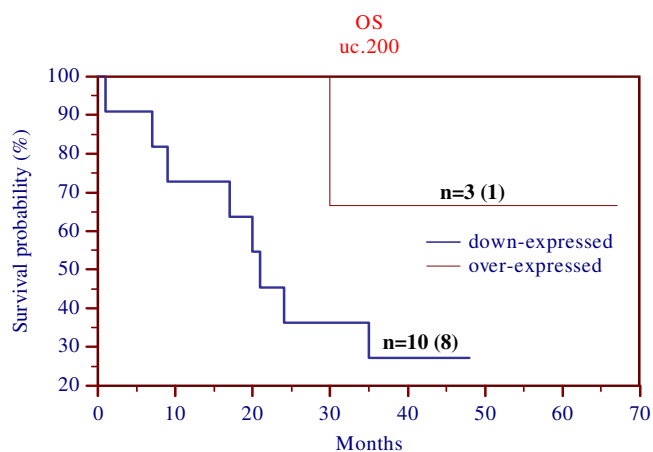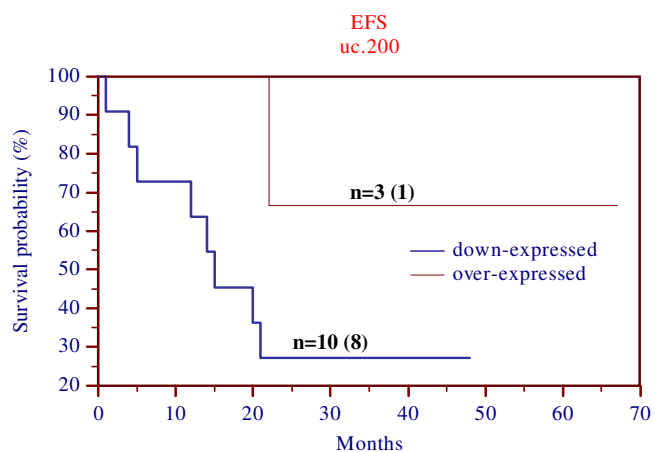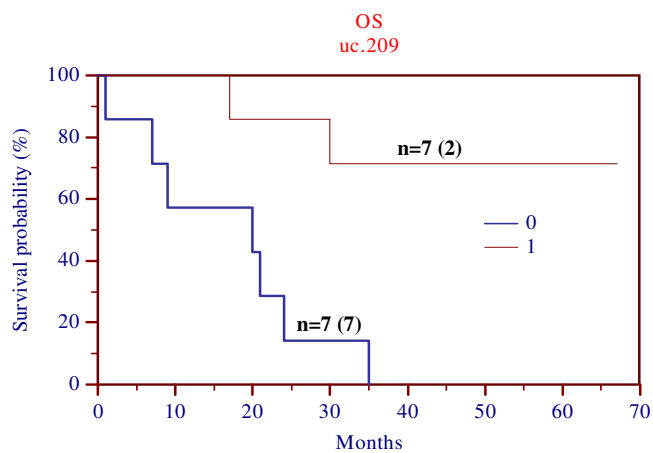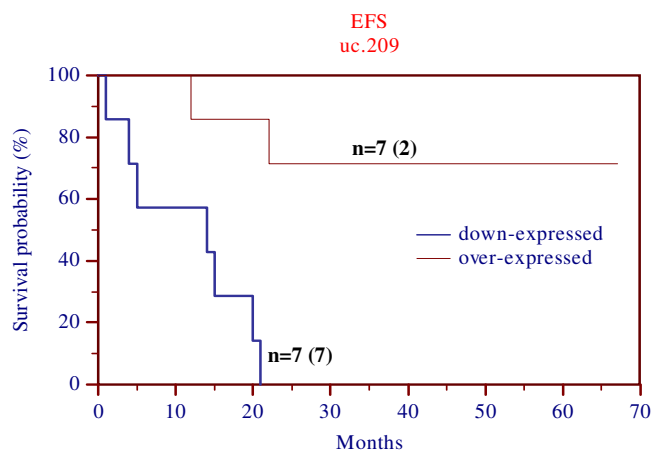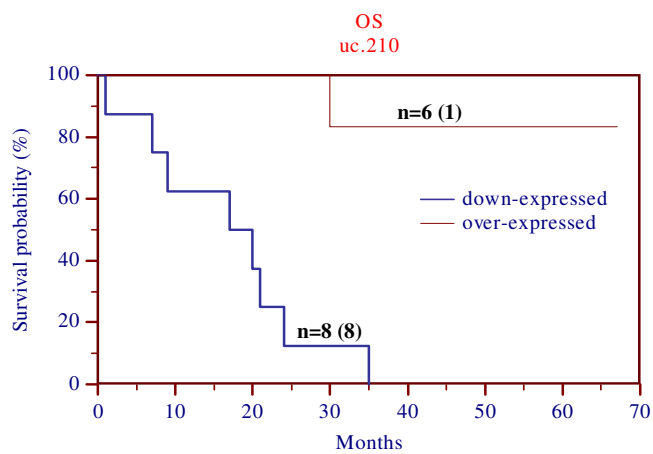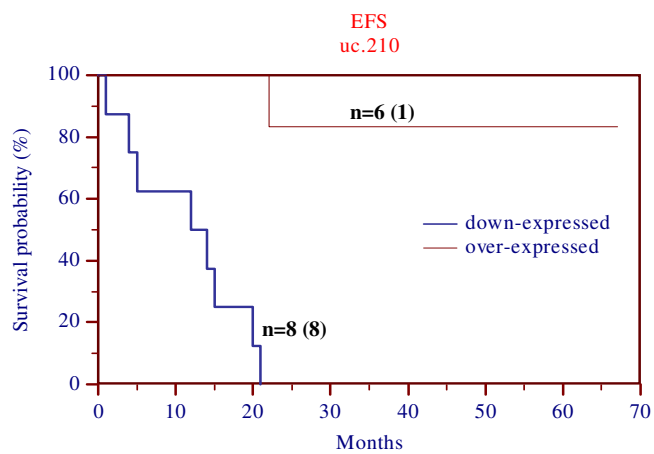

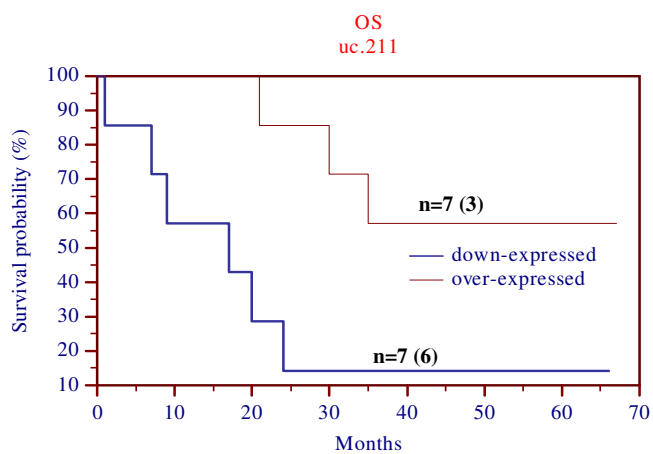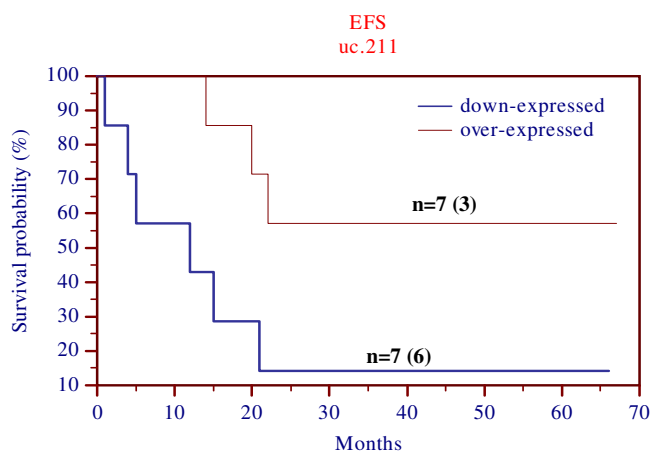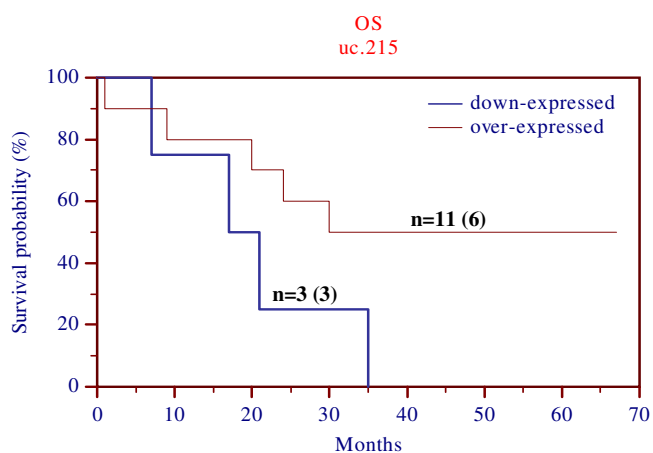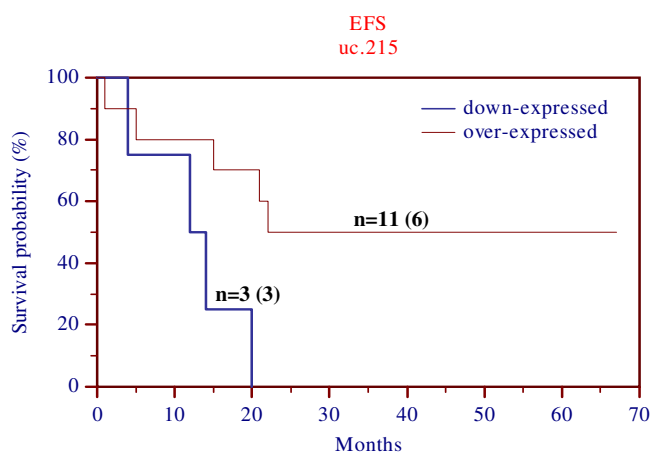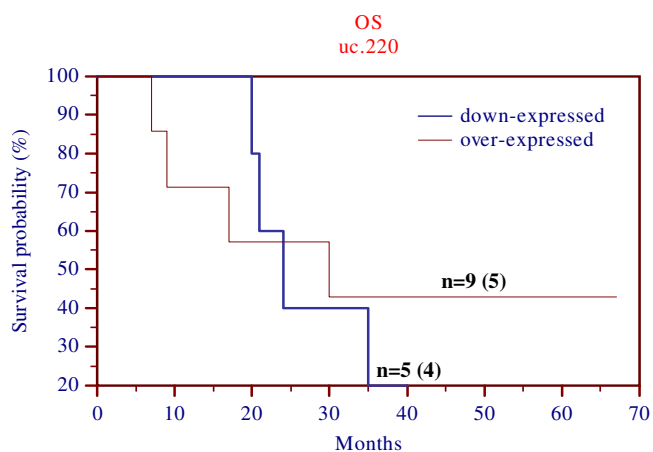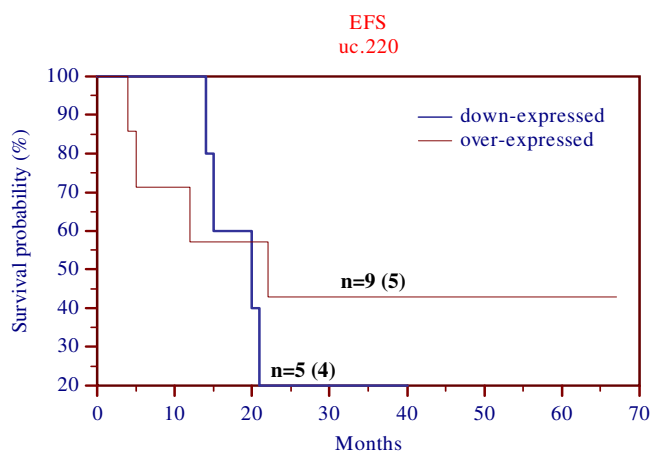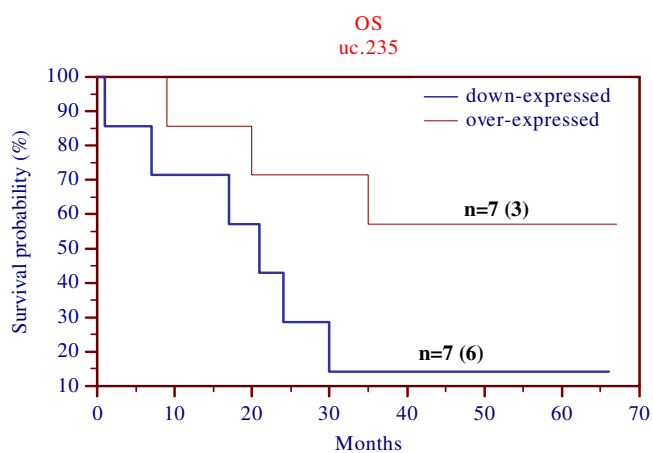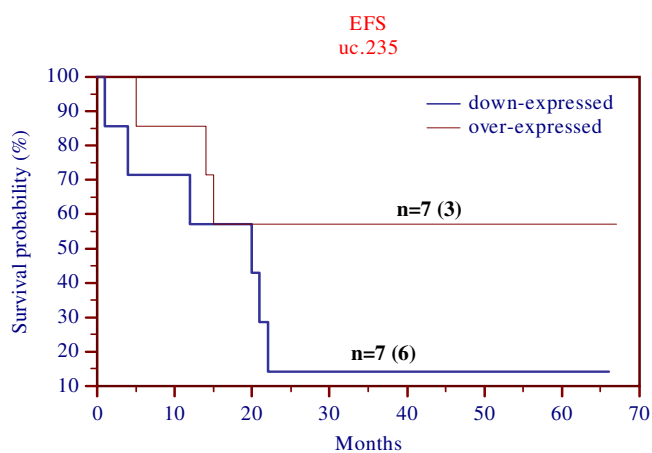

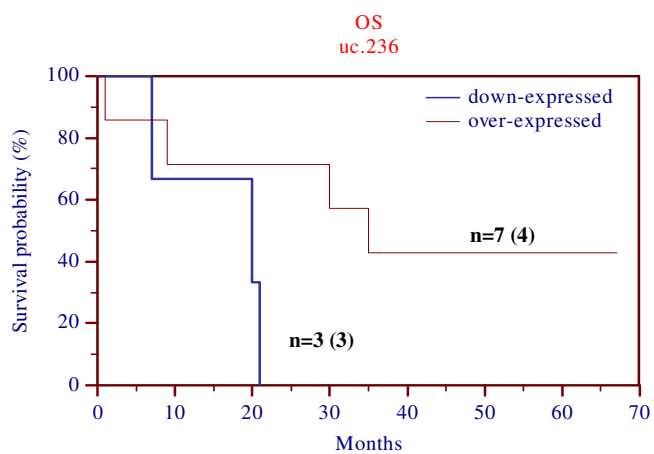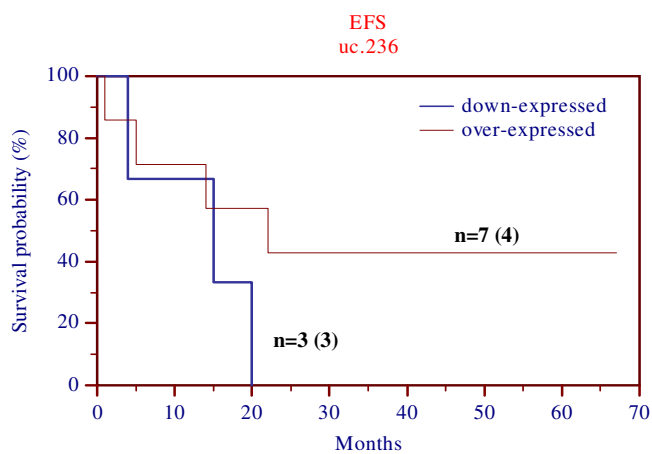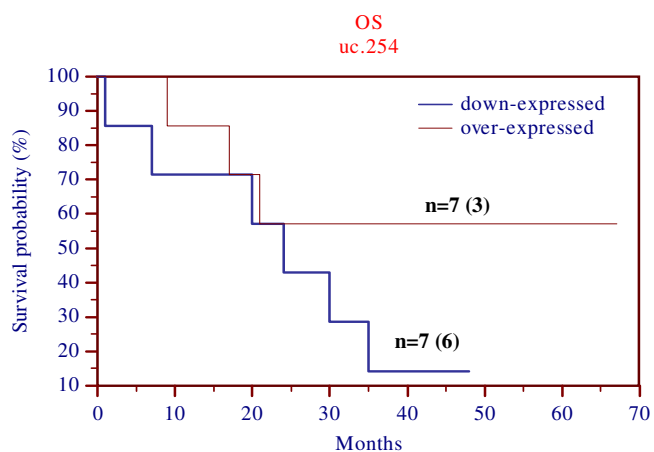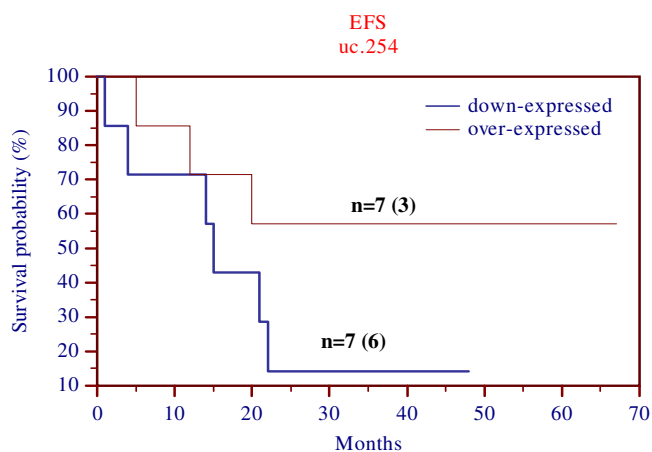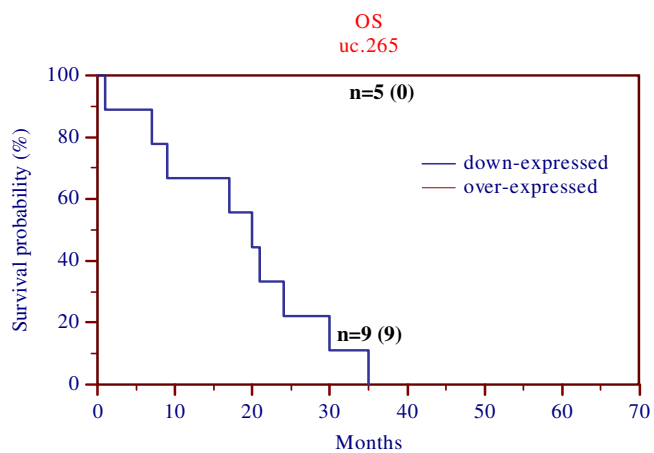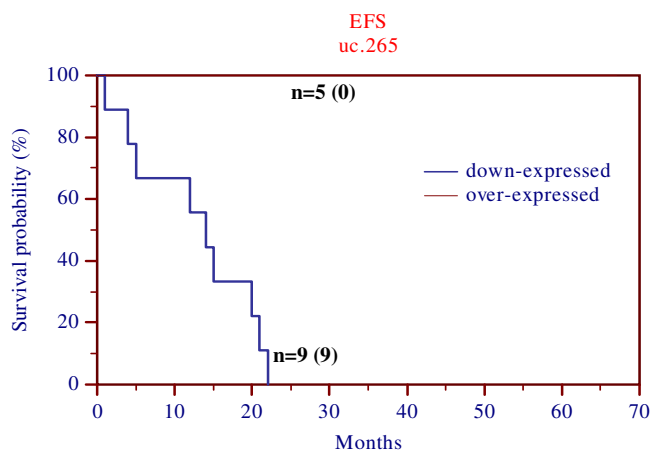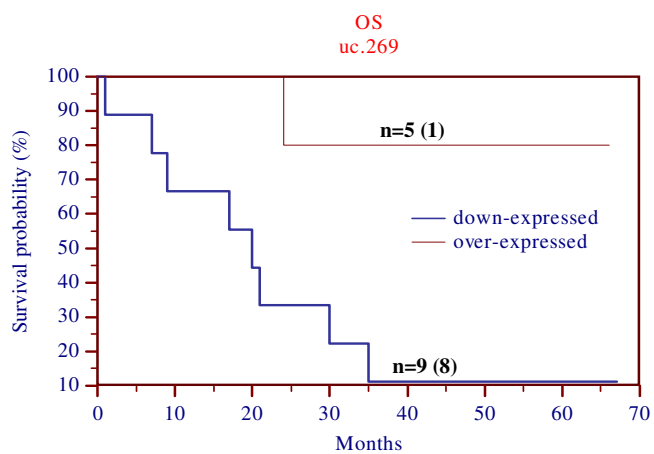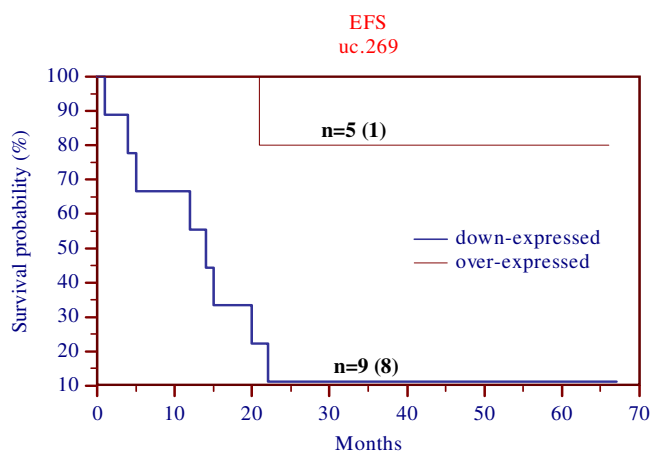

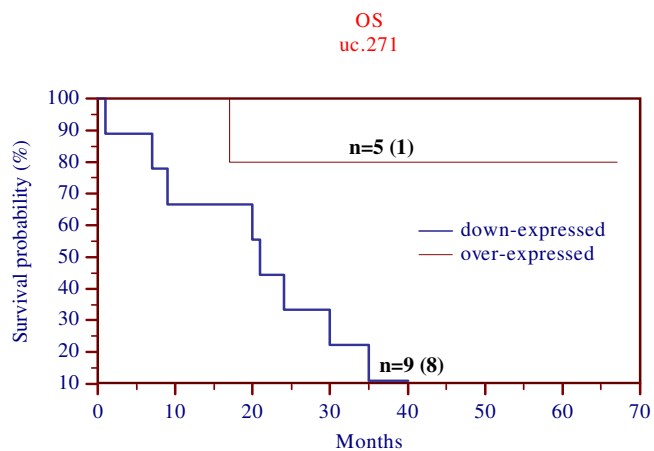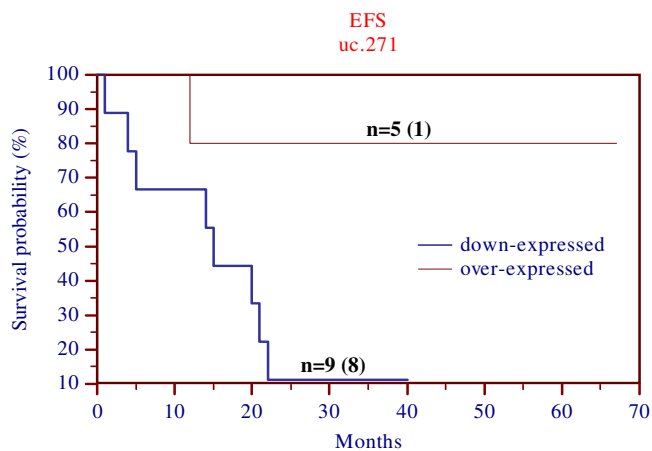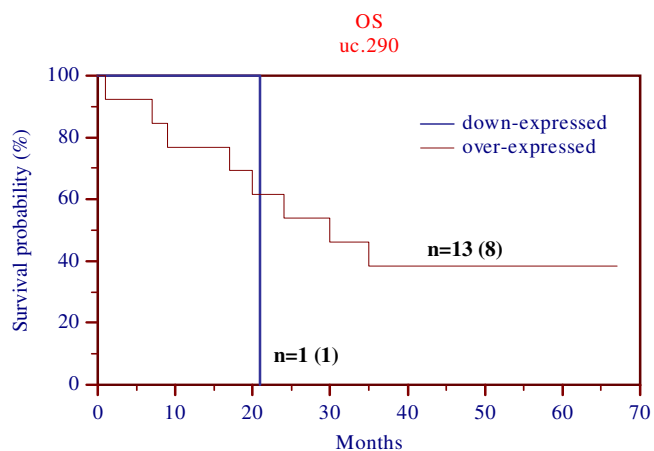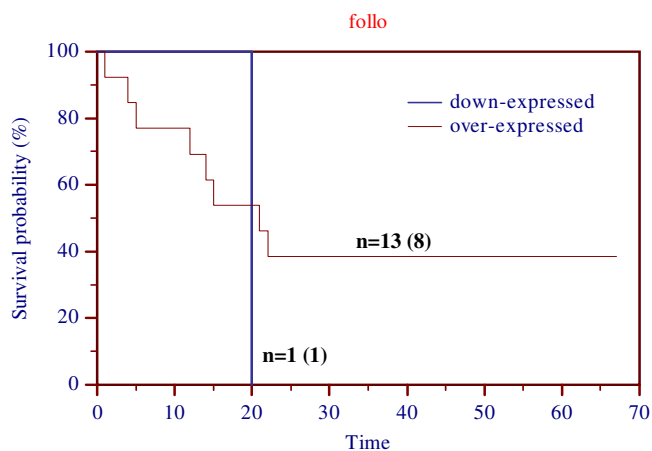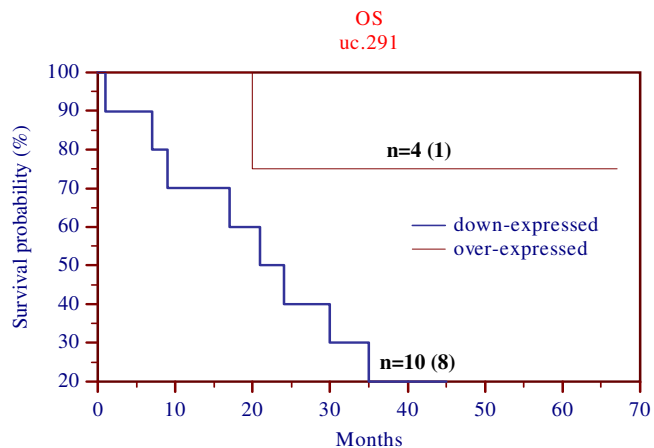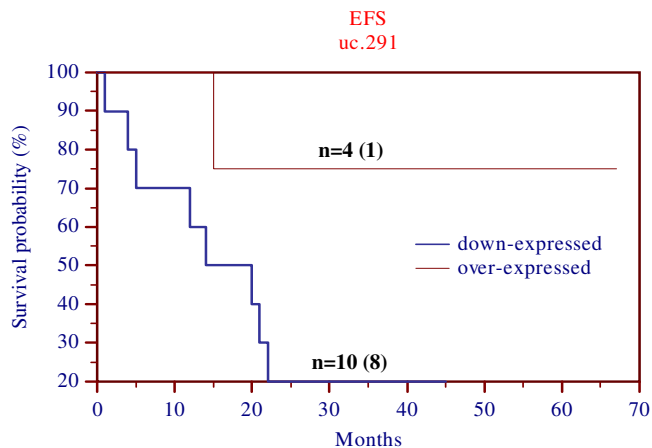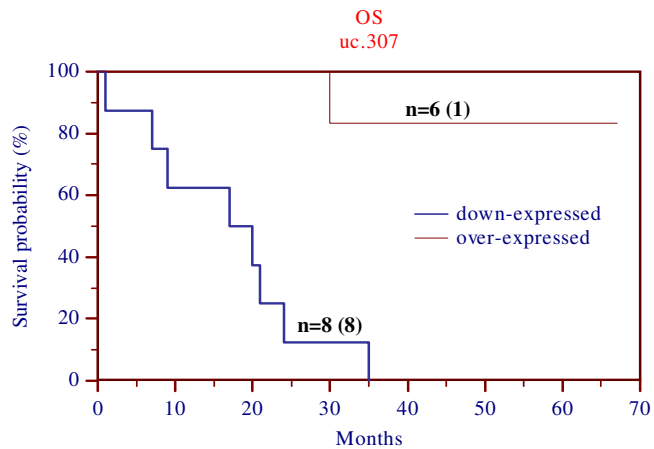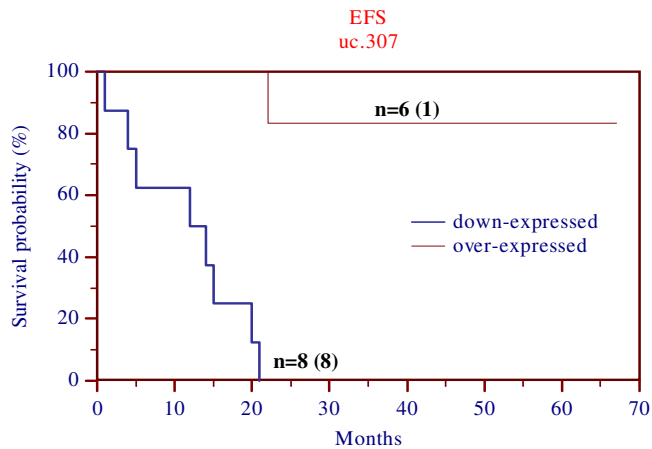

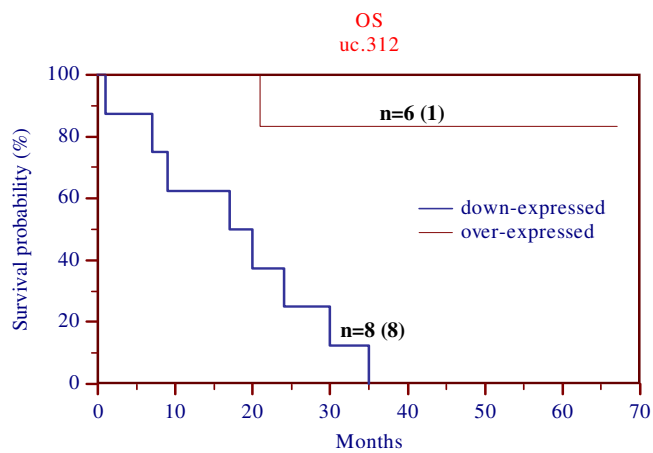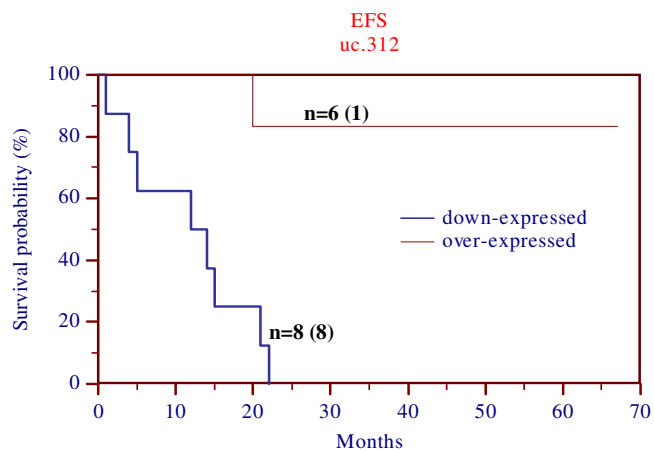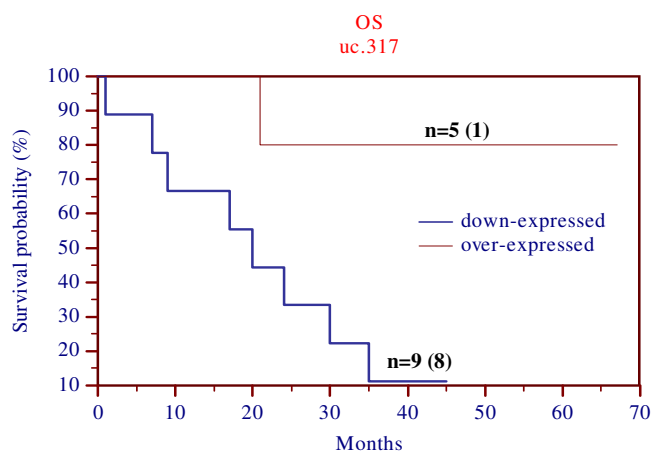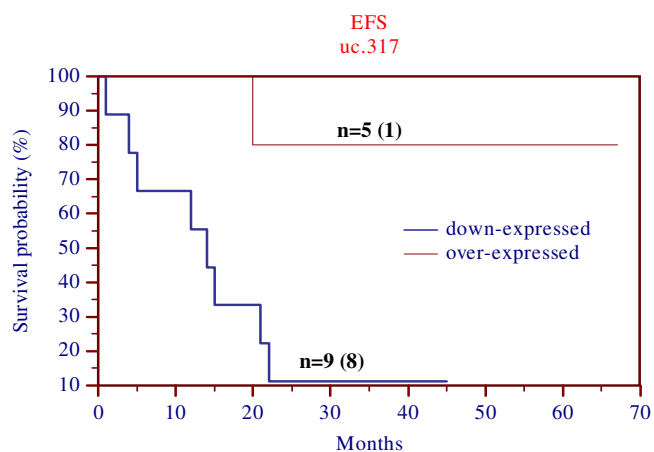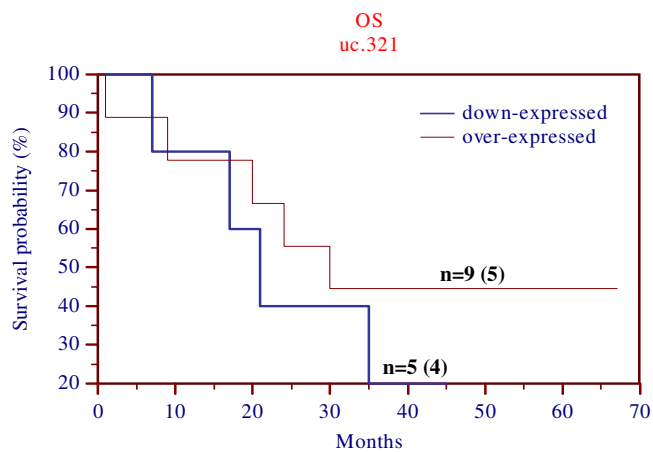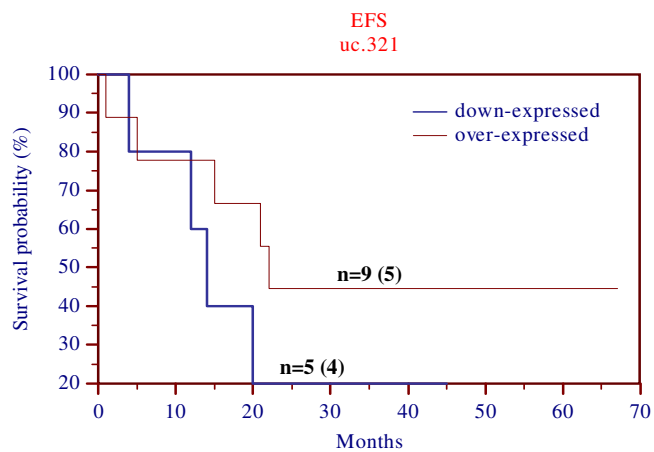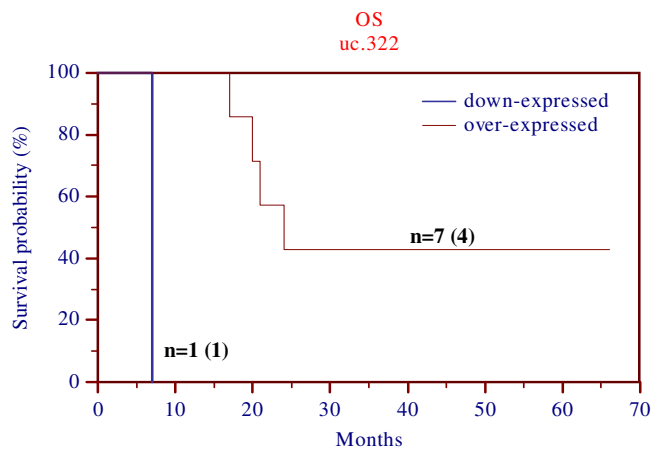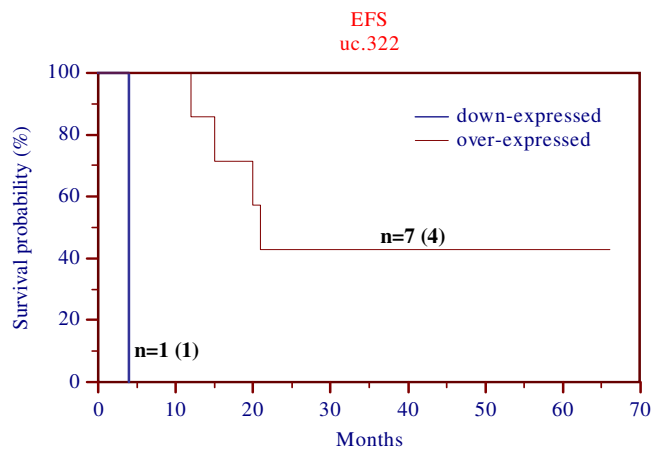

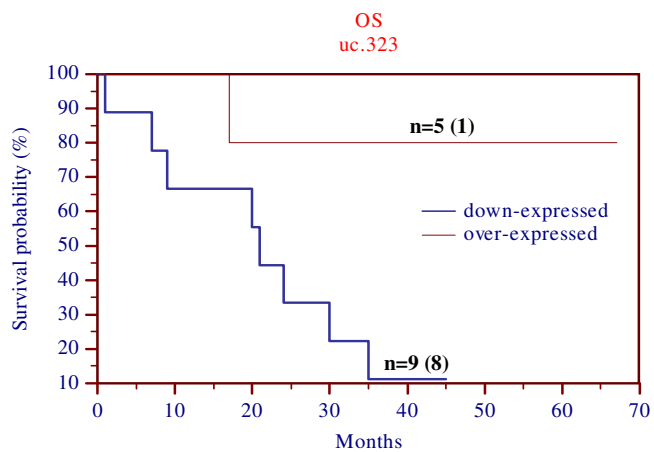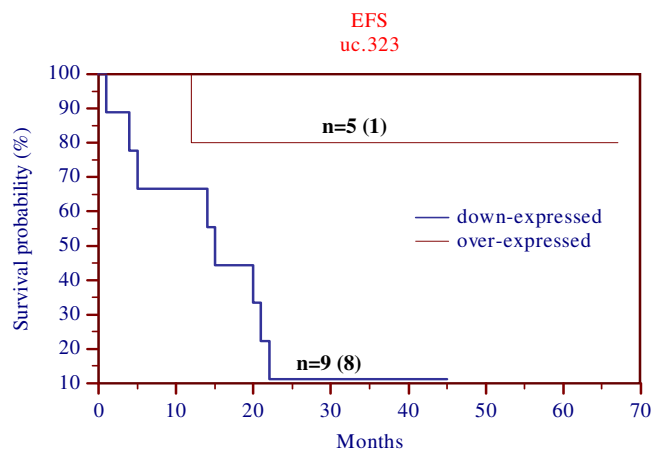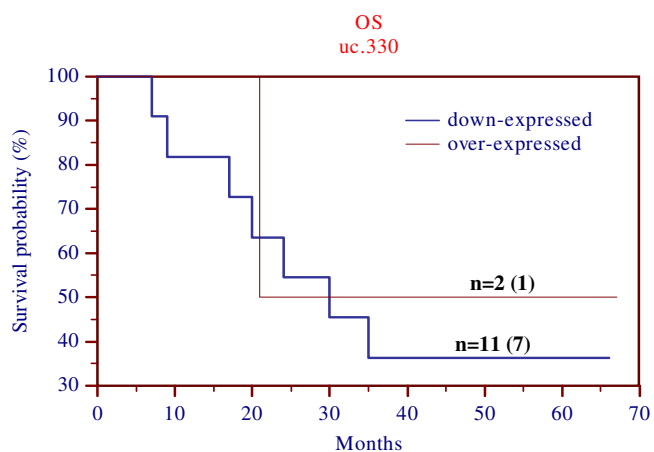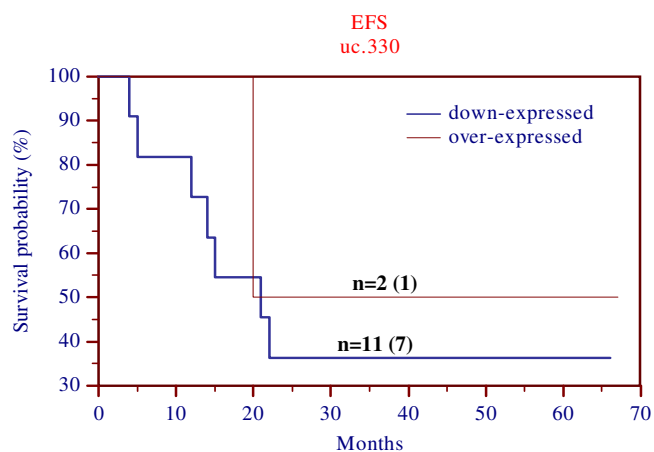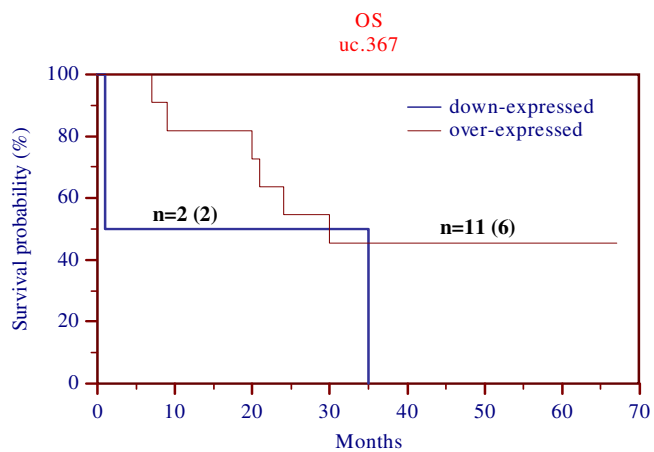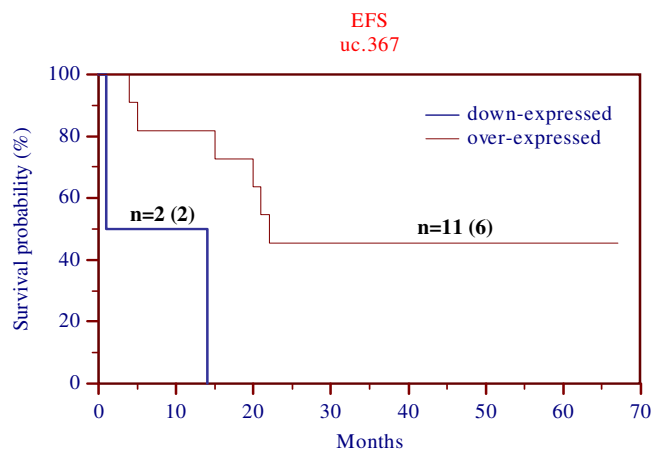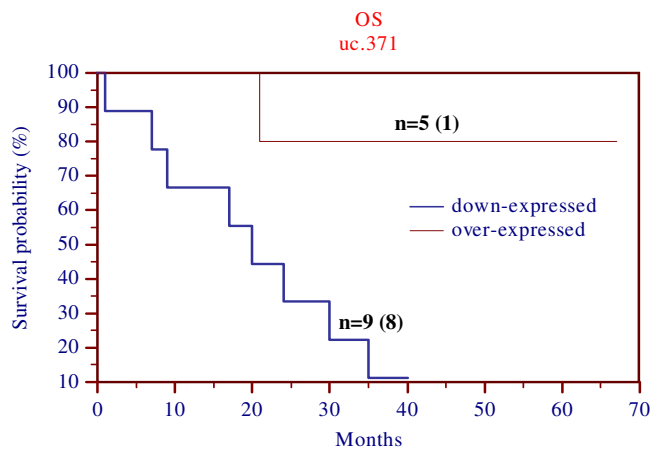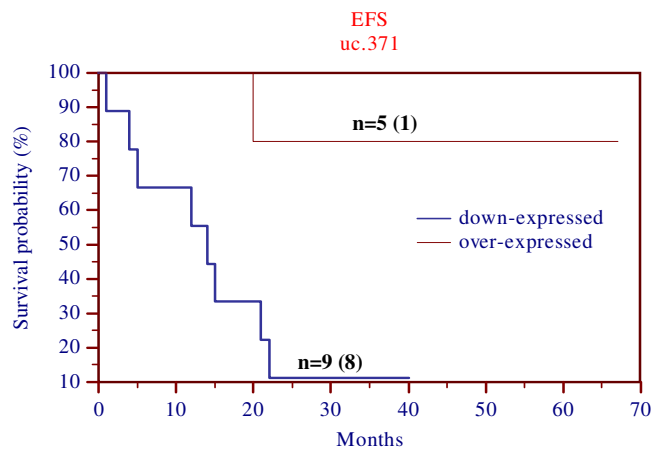

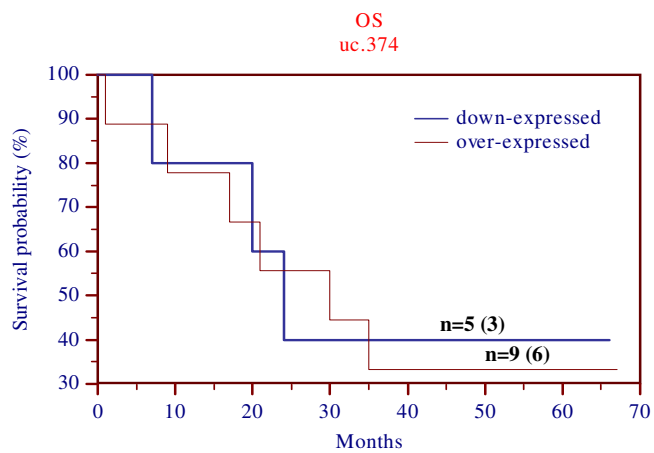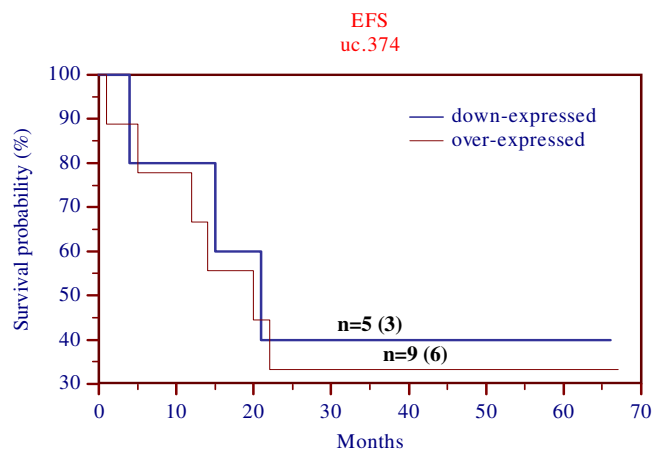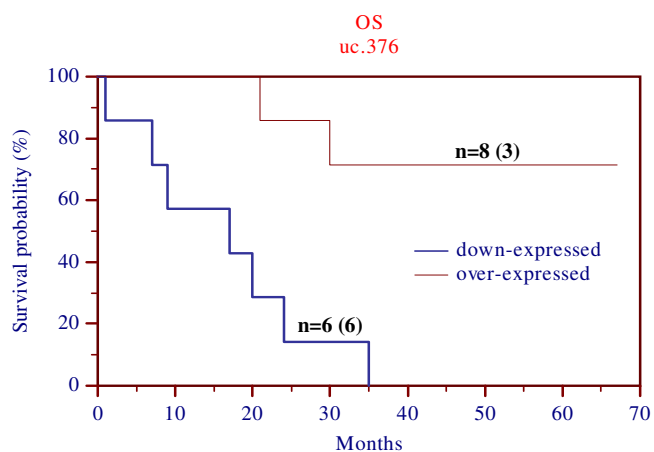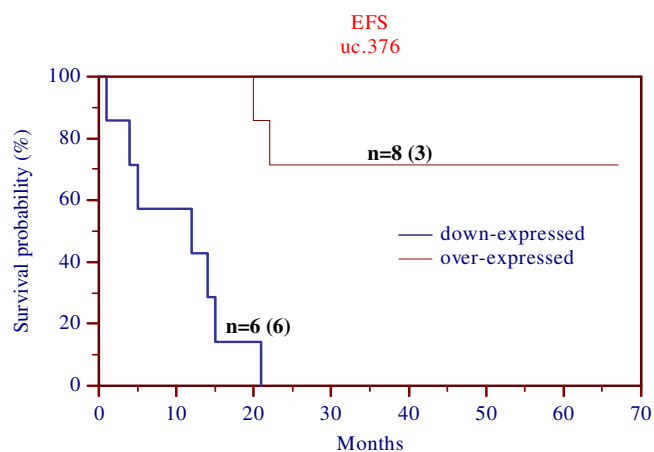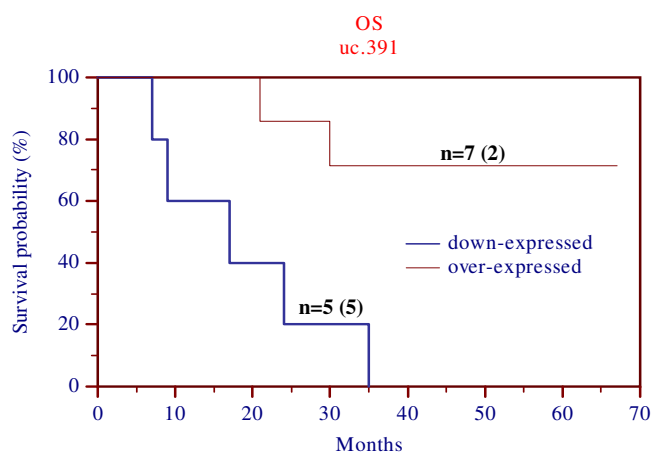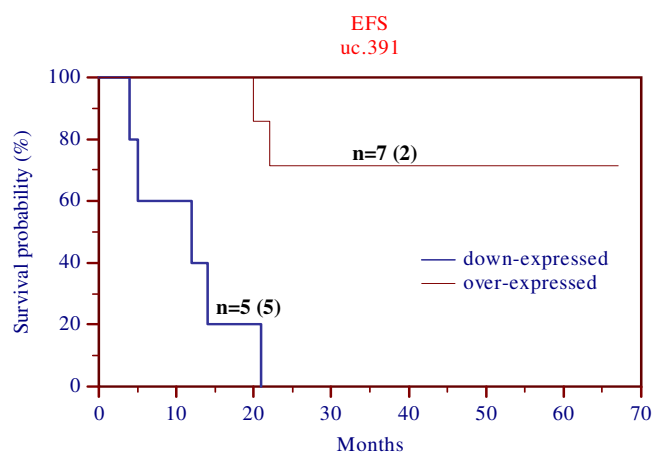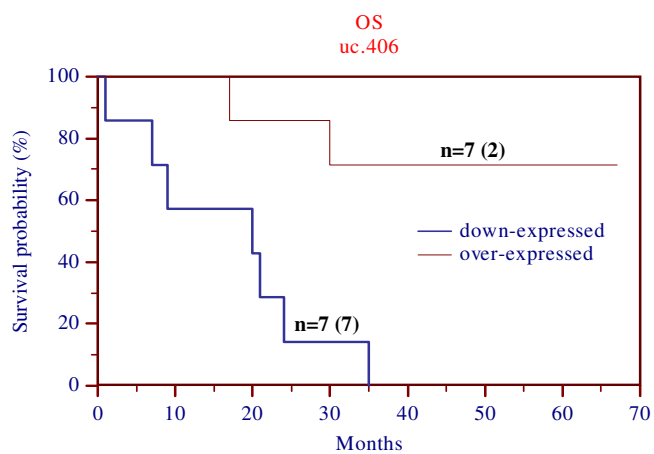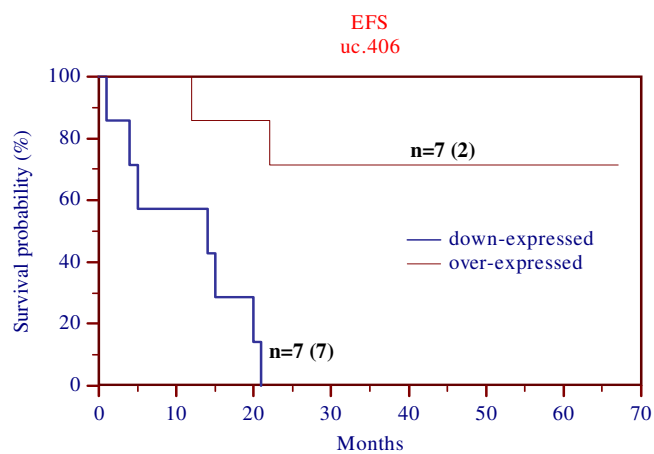

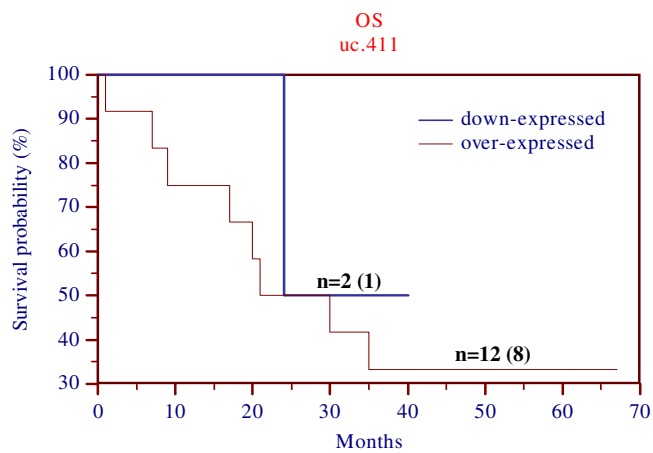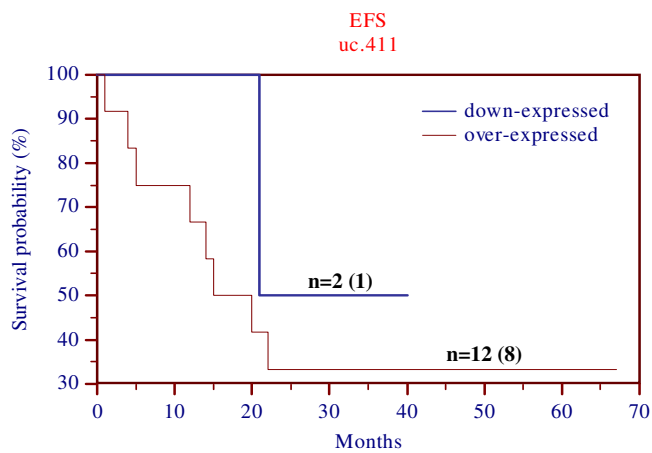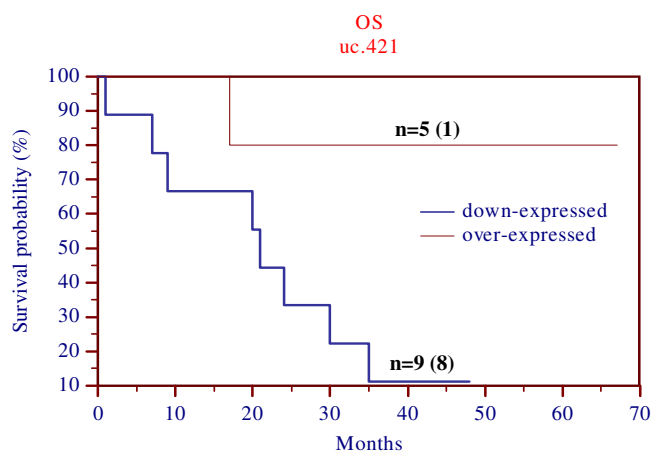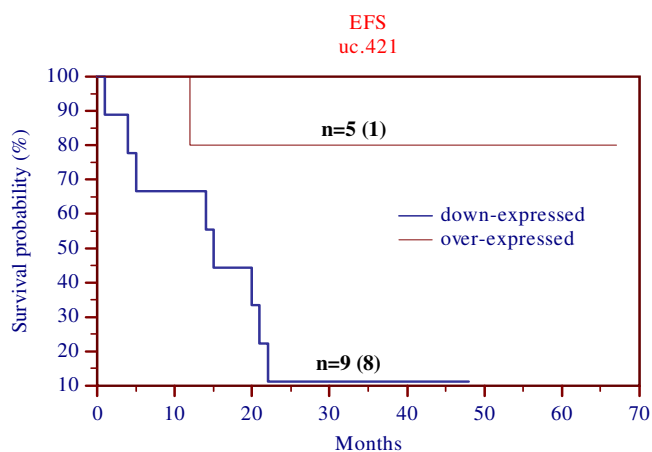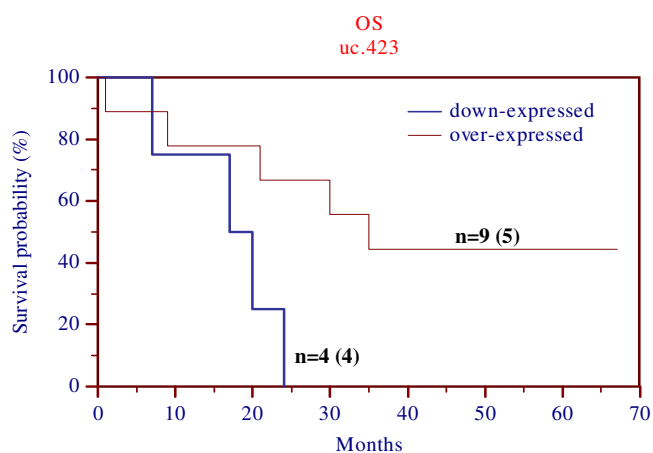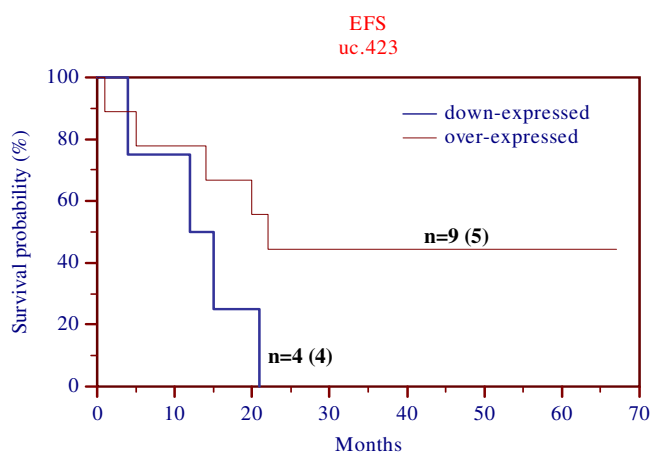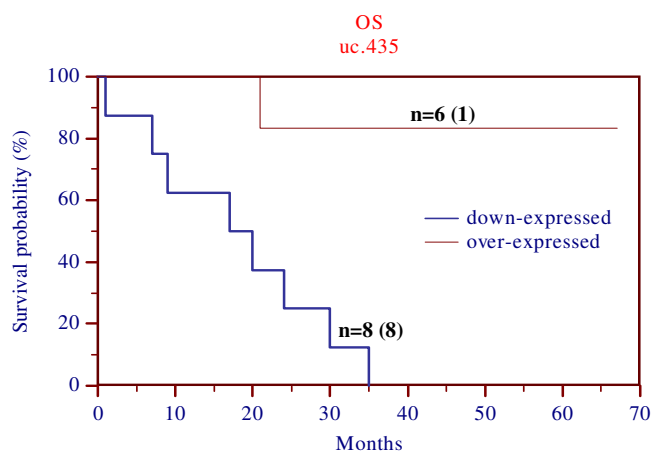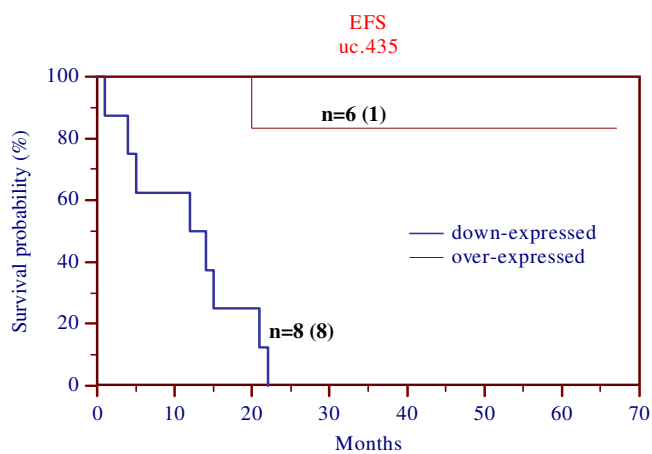

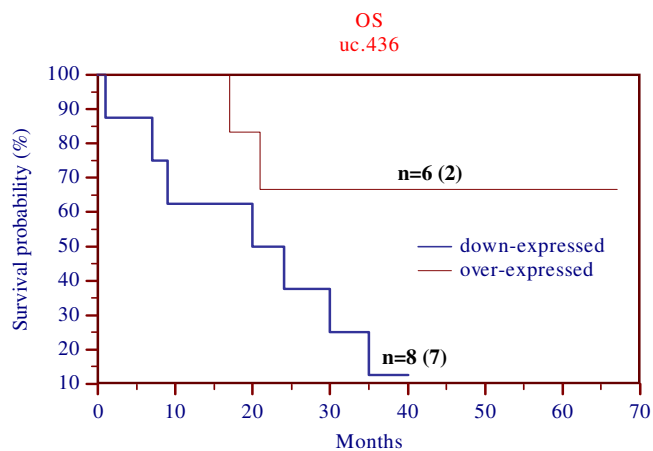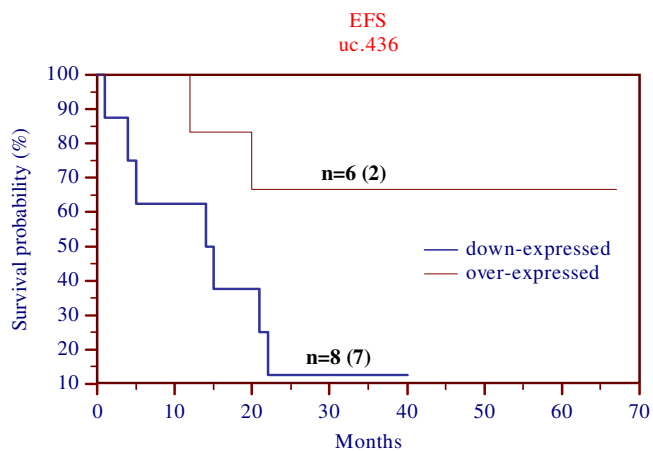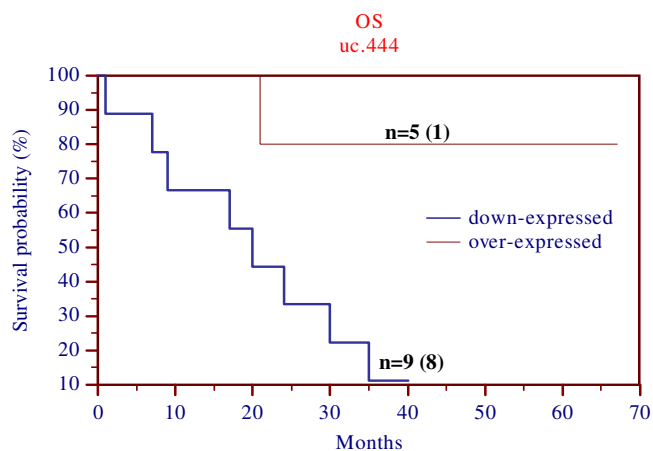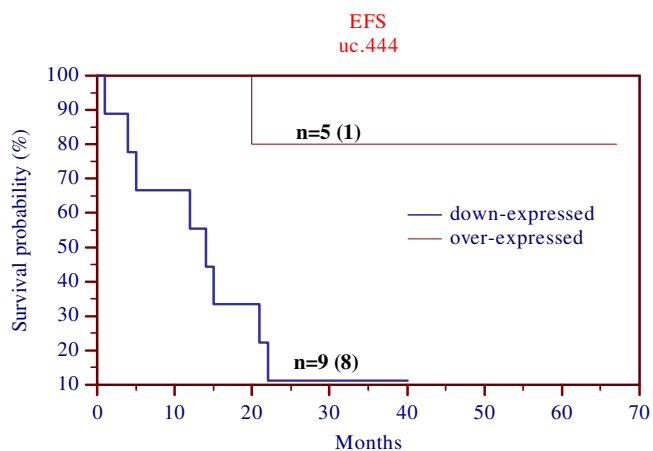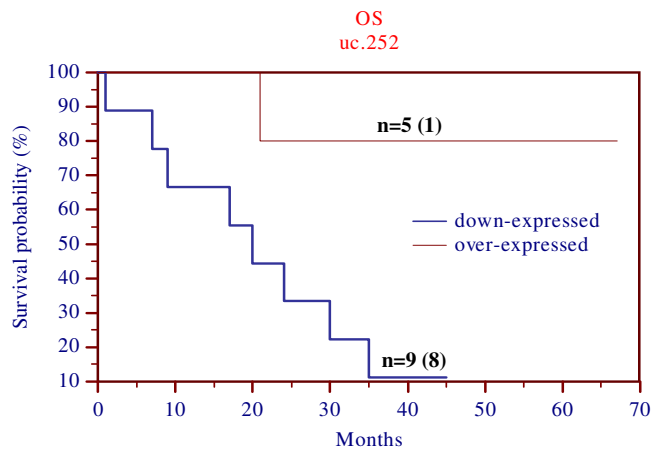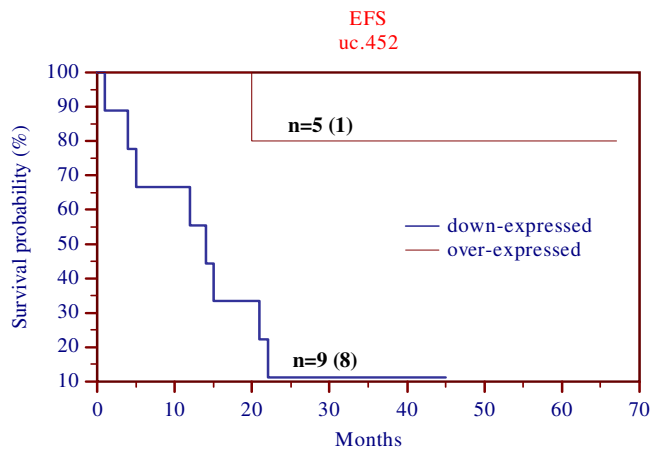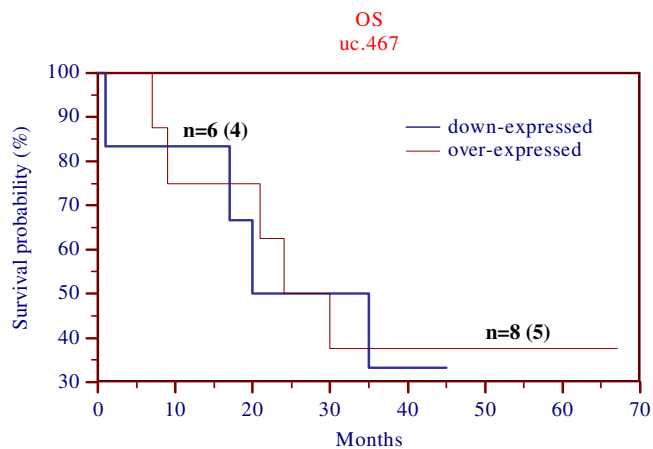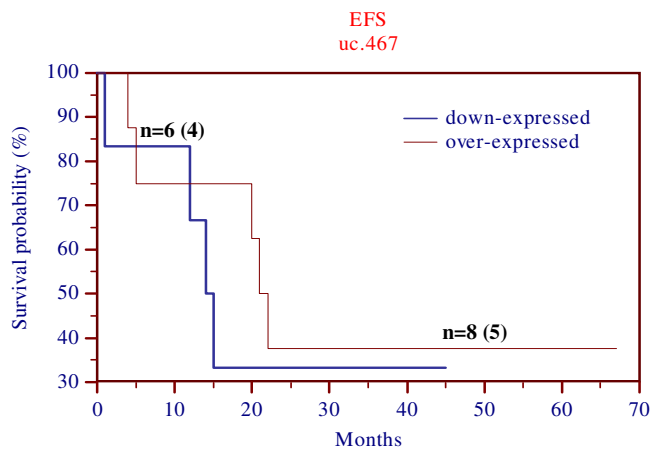

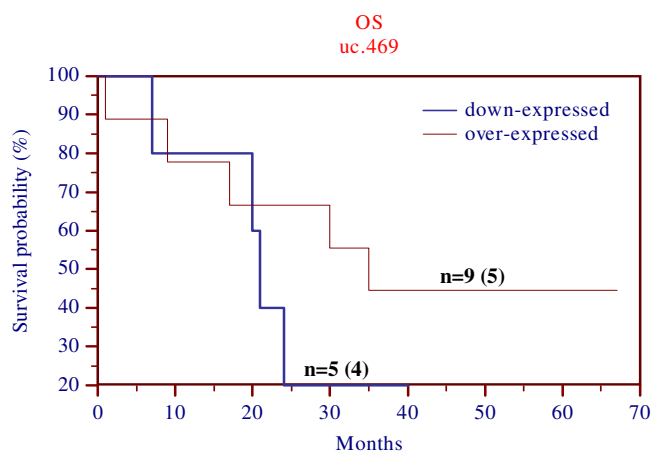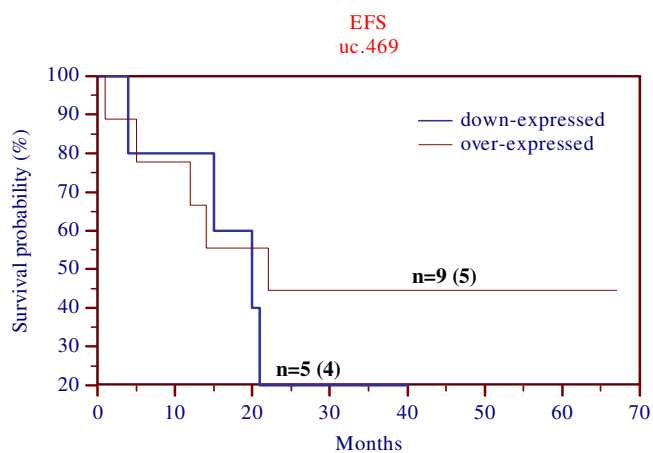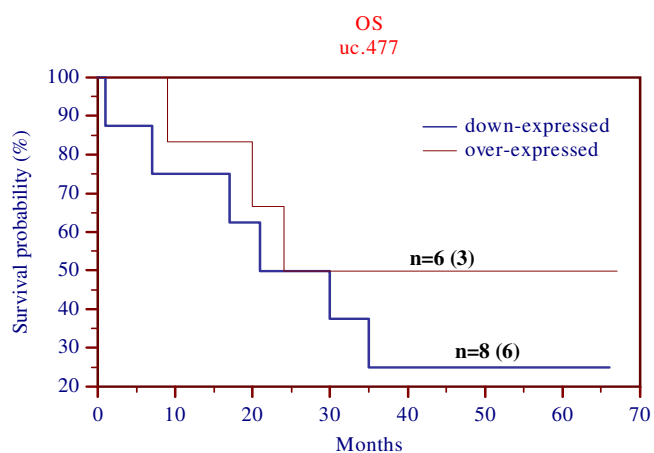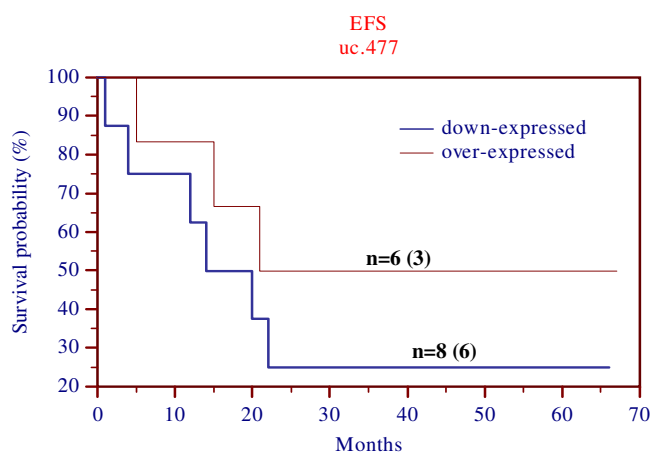

Supplement: Additional file 8 — Figure S4. Kaplan-Meier overall and event-free survival curves of the second set of 14 NB patients categorized according to T-UCR expression values (above or below the threshold defined in the first set of 20 NB samples by the respective ROC curve). In each graph are reported the number of patients in predicted subgroups and the number of patients with event (between brackets). [file 1471-2407-9-441-S8.PDF]

A

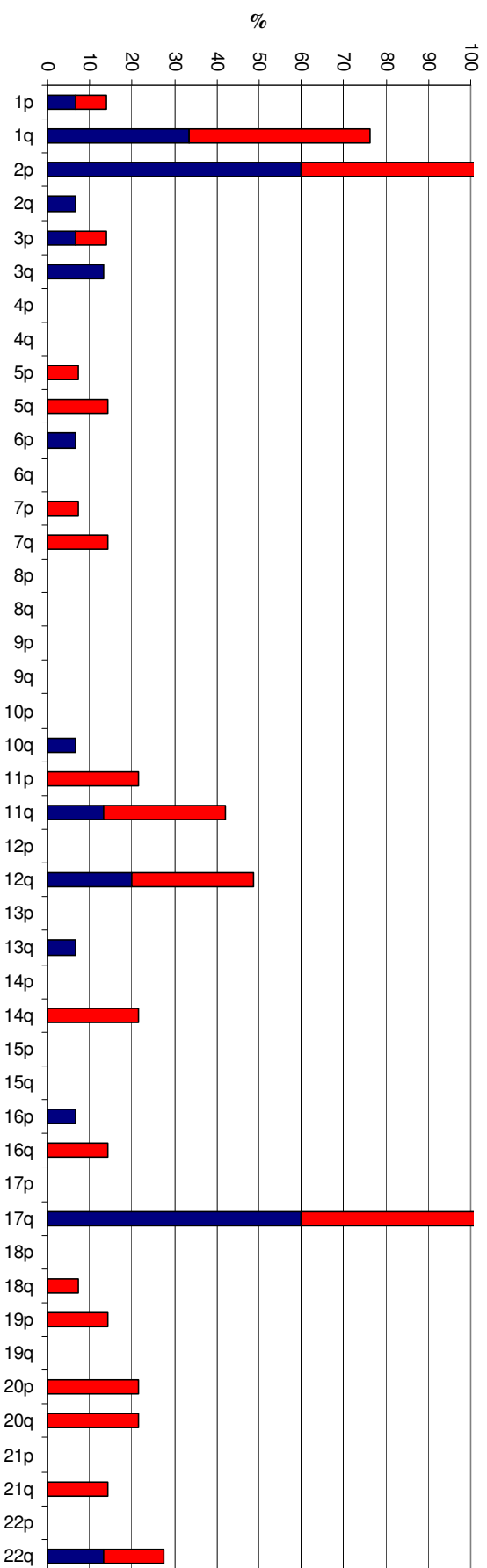

B

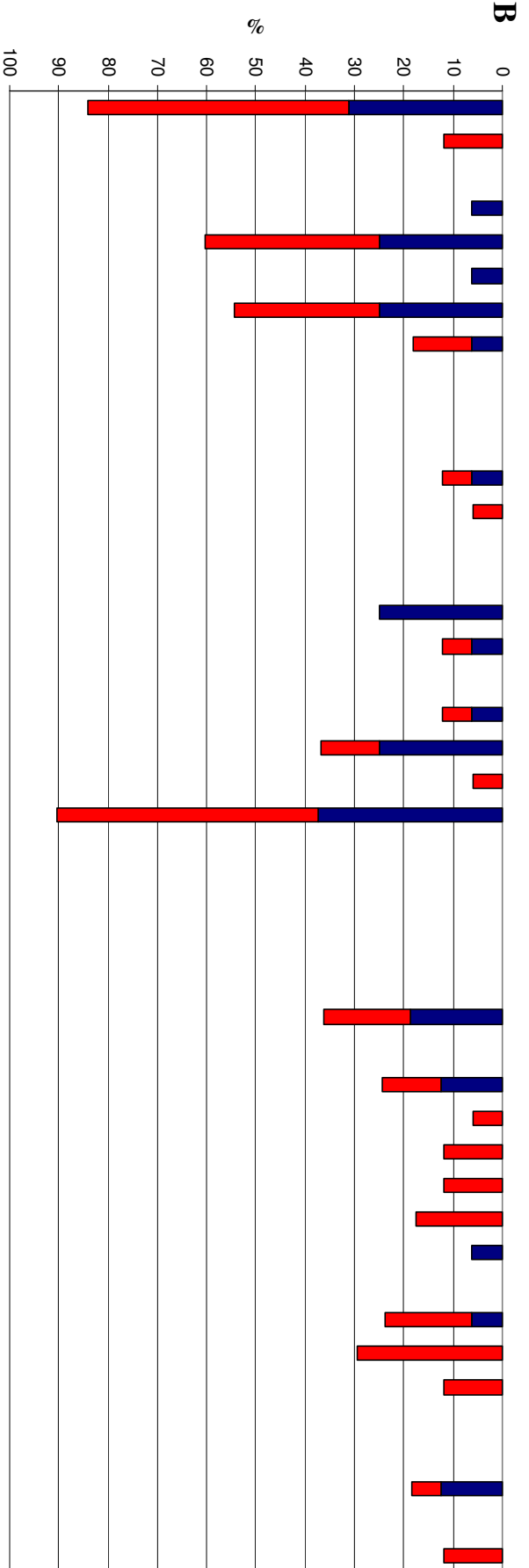

Supplement: Additional file 9 — Figure S5. Structural chromosomal aberrations. The Figure shows percentages of gains (A) and losses (B) for each of the autosomal chromosomes detected by array CGH in tumors of 14 short- and 15 long-survivors. [file 1471-2407-9-441-S9.PDF]
