# Supplementary material for: Transcribed-ultra conserved region expression is associated with outcome in high-risk neuroblastoma
Source: BMC Cancer. 2009 Dec 15;9:441. doi: 10.1186/1471-2407-9-441 (PMC2804711; doi:10.1186/1471-2407-9-441)
Supplement: Additional file 10 — Figure S6. Box plots of log2-transformed expression measures of the 13 microRNA differently expressed between long- and short-survivors. Each box represents the distribution of expression measured for "long-survivors" (blue, labeled by 0) and "short-survivors" (red, labeled by 1) tumor-category. [file 1471-2407-9-441-S10.PDF]

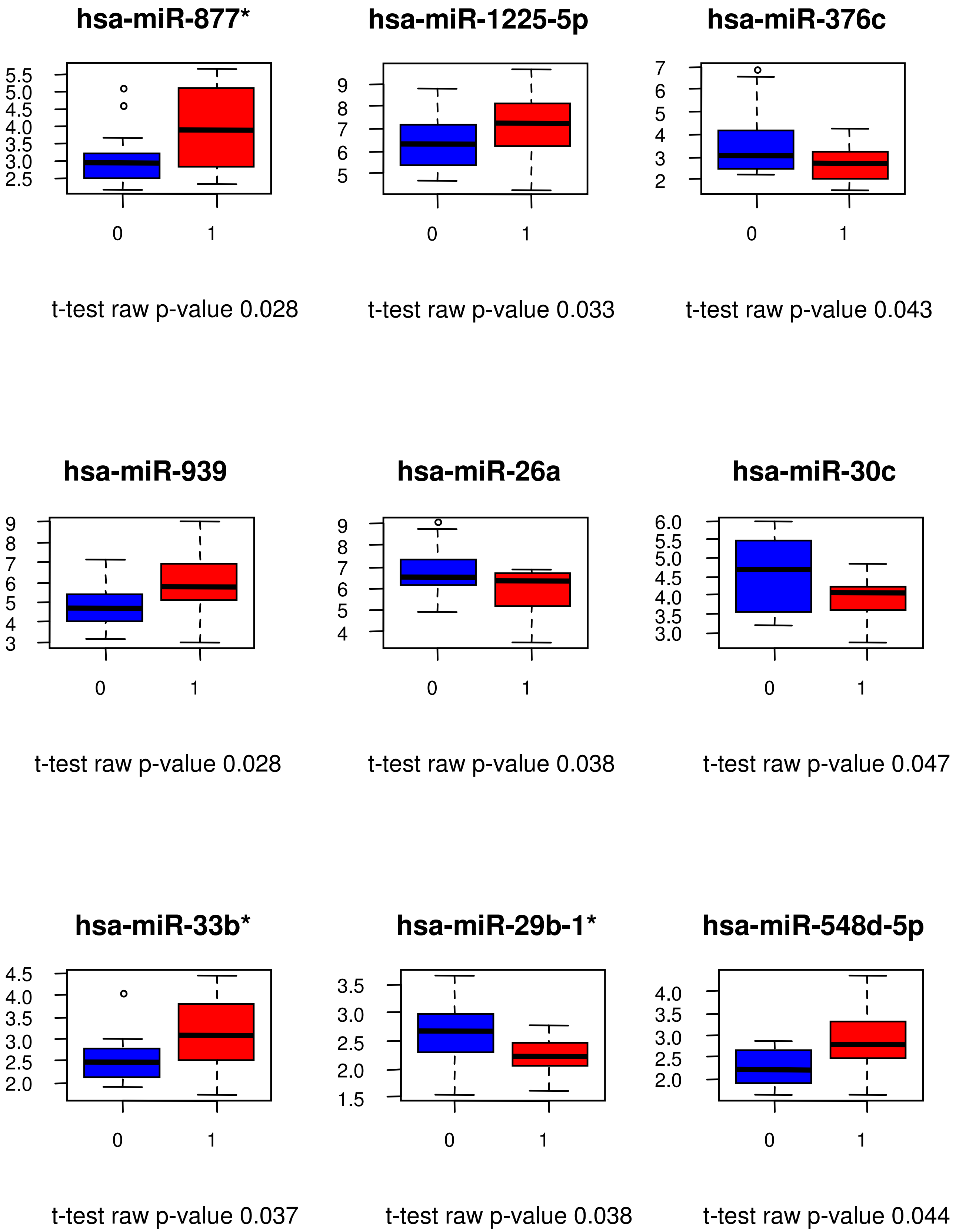
hsa-miR-877*
5.5
5.0
4.5
4.0
3.5
3.0
2.5
0
1
t-test raw p-value 0.028
hsa-miR-1225-5p
9
8
7
6
5
0
1
t-test raw p-value 0.033
hsa-miR-376c
7
6
5
4
3
2
0
1
t-test raw p-value 0.043
hsa-miR-939
9
8
7
6
5
4
3
0
1
t-test raw p-value 0.028
hsa-miR-26a
9
8
7
6
5
4
0
1
t-test raw p-value 0.038
hsa-miR-30c
6.0
5.5
5.0
4.5
4.0
3.5
3.0
0
1
t-test raw p-value 0.047
hsa-miR-33b*
4.5
4.0
3.5
3.0
2.5
2.0
0
1
t-test raw p-value 0.037
hsa-miR-29b-1*
3.5
3.0
2.5
2.0
1.5
0
1
t-test raw p-value 0.038
hsa-miR-548d-5p
4.0
3.5
3.0
2.5
2.0
0
1
t-test raw p-value 0.044

hsa-miR-1226

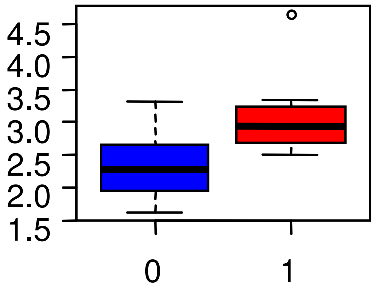

t-test raw p-value 0.035

hsa-miR-383

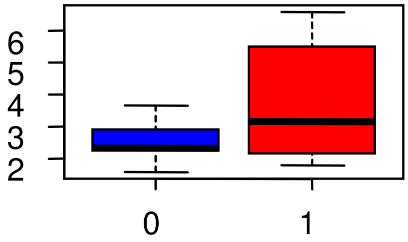

t-test raw p-value 0.02

hsa-miR-199b-5p

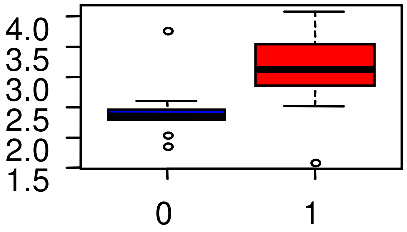

t-test raw p-value 0.025

hsa-miR-181a-2*

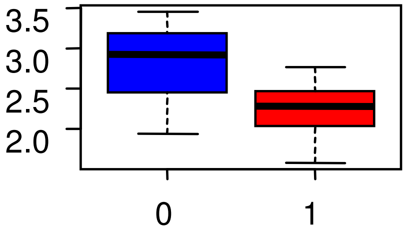

t-test raw p-value 0.014
