# Supplementary material for: Transcribed-ultra conserved region expression is associated with outcome in high-risk neuroblastoma
Source: BMC Cancer. 2009 Dec 15;9:441. doi: 10.1186/1471-2407-9-441 (PMC2804711; doi:10.1186/1471-2407-9-441)
Supplement: Additional file 11 — Table S3. T-UCRs associated with CpG islands in the promoter region of their own host genes. [file 1471-2407-9-441-S11.PDF]

Table S3. T-UCRs associated with CpG islands in the promoter region of their own host genes.

T-UCR	Within gene†	within or near CpG
uc.35	*DPYD*	Yes
uc.51	-	
uc.58	-	
uc.64	*NACSIN*	No
uc.70	*ARHGAP15*	No
uc.81	-	
uc.84	-	
uc.100	*ZAK*	Yes
uc.121	-	
uc.122	-	
uc.129	*MBNL1*	No
uc.165	-	
uc.167	-	
uc.185	*CLK4*	Yes
uc.189	*SFRS3*	Yes
uc.192	-	
uc.196	-	
uc.200	-	
uc.209	*TRA2A*	Yes
uc.210	-	
uc.211	*SKAP2*	Yes
uc.215	*GLI3*	No
uc.220	-	

uc.235	*DOCK5*	Yes
uc.236	-	
uc.254	-	
uc.265	*SLC44A1*	Yes
uc.269	*DENND1A*	Yes
uc.271	*MAPKAP1*	Yes
uc.290	*C10orf11*	No
uc.291	*C10orf11*	No
uc.307	*BTRC*	Yes
uc.312	*C10orf84*	Yes
uc.317	*MGMT*	Yes
uc.321	-	
uc.322	*SOX6*	No
uc.323	*SOX6*	No
uc.330	*RBM14*	Yes
uc.367	*NPAS3*	Yes
uc.371	*GARNL1*	Yes
uc.374	*MIPOL1*	Yes
uc.376	*PRPF39*	Yes
uc.391	*MAP2K5*	Yes
uc.406	*NFAT5*	Yes
uc.411	*AATF*	Yes
uc.421	*ZNF521*	Yes
uc.423	*ZNF521*	Yes
uc.435	*TCF4*	Yes

uc.436	*TCF4*	Yes
uc.444	*C19orf2*	Yes
uc.452	*TSHZ3*	Yes
uc.467	*POLA*	Yes
uc.469	-	
uc.477	*PLP1*	No

†Unigene ID
